# Dating the End of the Greek Bronze Age: A Robust Radiocarbon-Based Chronology from Assiros Toumba

**DOI:** 10.1371/journal.pone.0106672

**Published:** 2014-09-15

**Authors:** Kenneth Wardle, Thomas Higham, Bernd Kromer

**Affiliations:** 1 Department of Classics, Ancient History and Archaeology, University of Birmingham, Birmingham, United Kingdom; 2 Oxford Radiocarbon Accelerator Unit, Research Laboratory for Archaeology and the History of Art, University of Oxford, Oxford, United Kingdom; 3 Akademie der Wissenschaften Heidelberg, Heidelberg, Germany; New York State Museum, United States of America

## Abstract

Over 60 recent analyses of animal bones, plant remains, and building timbers from Assiros in northern Greece form an unique series from the 14^th^ to the 10^th^ century BC. With the exception of Thera, the number of ^14^C determinations from other Late Bronze Age sites in Greece has been small and their contribution to chronologies minimal. The absolute dates determined for Assiros through Bayesian modelling are both consistent and unexpected, since they are systematically earlier than the conventional chronologies of southern Greece by between 70 and 100 years. They have not been skewed by reference to assumed historical dates used as priors. They support high rather than low Iron Age chronologies from Spain to Israel where the merits of each are fiercely debated but remain unresolved.

## Introduction

Until very recently the chronology of the later part of the Aegean Bronze Age was entirely based on ‘historical’ dates derived from Egypt with the aid of exported or imported objects such as Minoan or Mycenaean pottery or Egyptian scarabs. Dates based on ^14^C dating methods have had wide error margins and the complexities of the calibration curve for the final centuries of the second millennium BC preclude the precise dating of a single sample using ^14^C techniques alone. Even where the samples and dating techniques are more varied, as in the case of the array of absolute dates determined for the Thera eruption, these have been viewed by some archaeologists with suspicion, particularly since they are offset from the conventional chronology by around 100 years and remain the subject of lively debate [1]. Recent analyses of material from Egypt have, however, confirmed that the Egyptian ^14^C and historical chronologies are compatible and strengthen our conviction that the Thera ^14^C dates are correct [2]–[5] Studies of material from Argos [6] and Aegina [7] in Greece and more widely in the Eastern Mediterranean [8] all lead to similar conclusions. In Greece substantial pieces of wood charcoal suitable for dendrochronological determination are exceptionally rare and it is not yet possible to link those available with the near-absolutely placed Anatolian conifer (core) sequence. Similarly, few sites have provided more than a handful of charcoal samples, usually far from ideal for dating purposes. Although the precision of ^14^C measurements has improved steadily and Bayesian modelling has provided a powerful tool for the analysis of the results, these results can be no better than the quality of the samples available.

**Figure 1:**
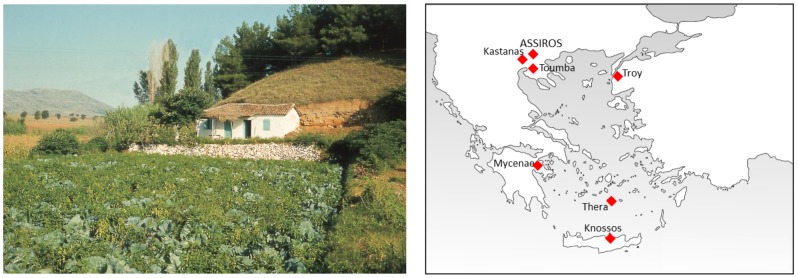
Assiros Toumba: the site and its location. The 14 m high tell is formed from the debris of an unbroken, thousand year long sequence of building levels dating between c 2000 and 1000 BC.

At Assiros in northern Greece [9] (Fig. 1), however, a combination of meticulous excavation, careful sample selection and good fortune has provided the first long, robust sequence of determinations from Greece for the later part of the Bronze Age and the start of the Iron Age. A large number of high precision ^14^C determinations have been obtained from samples of three different types: charred building timbers, charred seeds and the collagen extracted from a stratified sequence of domestic animal bones (Section A in File S1). An uninterrupted stratigraphic sequence of building levels covers more than 400 years, while the preservation of substantial charred structural timbers from four phases has enabled precise dates to be established for the cutting of these timbers, using the technique of dendrochronological wiggle-matching (DWM). Quantities of crop seeds from a series of granaries have also been closely dated. Determinations of well-stratified animal bone samples representing every phase allowed us to test, through the application of Bayesian modelling techniques, whether the ‘old wood effect’, often cited as the reason for preferring historical dates to scientific ones based on wood charcoals, could be ruled out for the timbers at Assiros.

**Figure 2:**
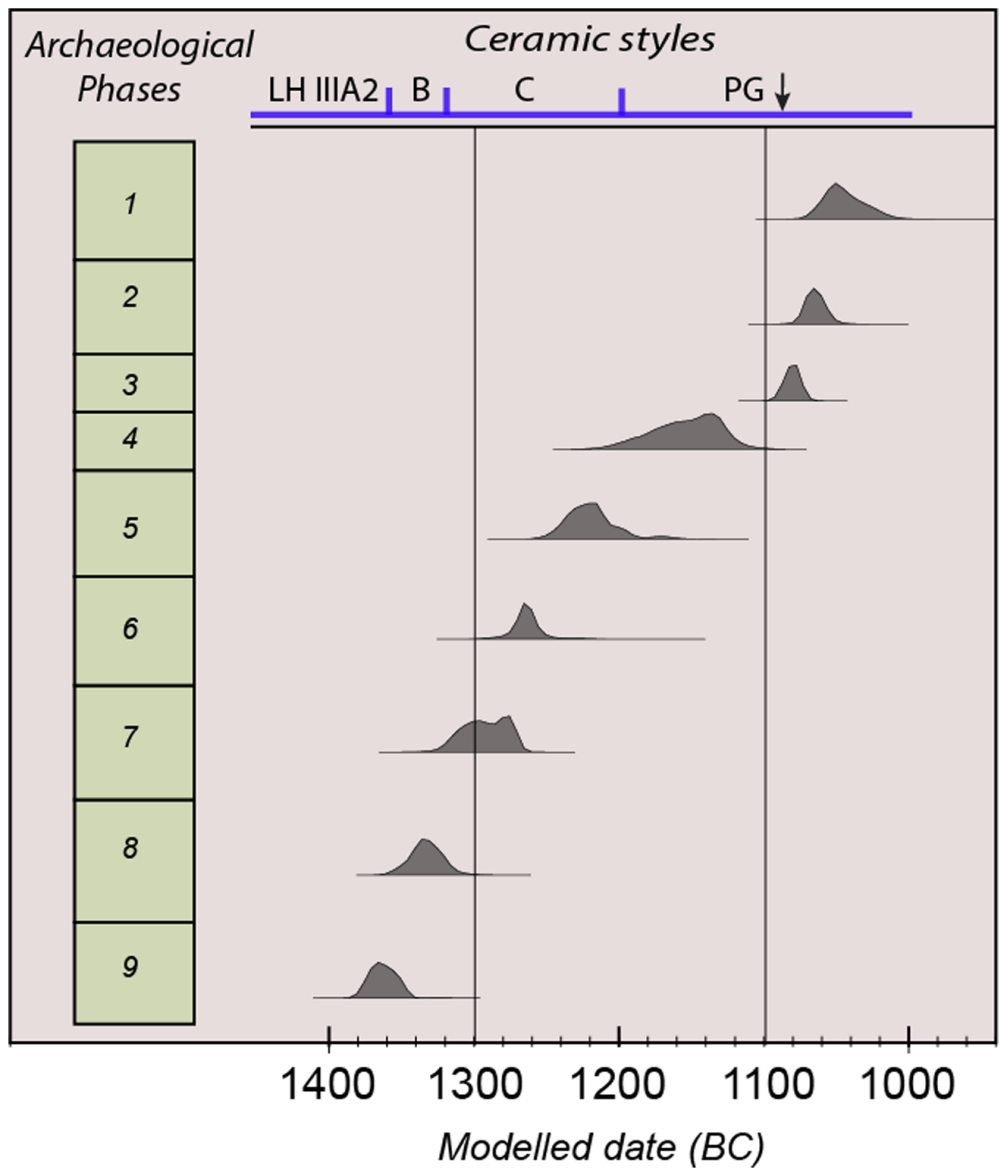
Modelled dates for each phase of the Assiros sequence. The date ranges are the summed probability distributions for each of the dated phases. These are shown for illustrative purposes only. See Figure S2 in File S1 for the full Bayesian model and Table S11 in File S1 for the model code.

This series of dates from well-stratified long- and short-life samples from a single site, is unique in the Eastern Mediterranean and has radically improved our picture of ^14^C-based chronology for the Greek Bronze Age. It is shown in Fig. 2 in diagrammatic form as summed probability distributions for each of the phases. It provides, for the first time, a sequence of absolute dates which are in no way mediated by reference to historical context or predicted duration of any phase. The robust nature of this sequence, offset from the existing conventional chronologies by between +100 and +70 years, requires us to reconsider dates based on tenuous links with distant historical chronologies, especially for the Mycenaean and Proto-Geometric sequences (Section C in File S1). As at Thera, they call into question traditional assumptions about historical chronologies. They are especially important at the end of the Greek Bronze Age since they impact upon the vigorous debates surrounding the absolute dates of developments in Israel, in the circum-Alpine region and the Iberian peninsula [10], [11] (Section E in File S1). In the same way a recently published Bayesian analysis of short life samples from Southern Italy helps to establish an absolute chronology for the Central Mediterranean Bronze Age independently of Aegean ceramic-based chronologies [12].

## Materials

### The site and the archaeological materials (Section B in File S1)

Assiros Toumba is a settlement mound situated at the NE end of the Langadas Basin, some 25 km inland from modern Thessaloniki. Its form and history is typical of the many such mounds to be found at intervals of 5–10 km in lowland Central Macedonia. Measuring 100×70 m at its base, it is of average size. The steep-sided profile, rising to 14 m above the surrounding area, is the result of the repeated re-construction of the substantial terrace banks around its perimeter which served both for defence and to support the buildings on the summit. First established c 2000 BC, the settlement appears to have been continuously occupied until early in the Iron Age (Phase 1.5) – perhaps until c 1000 BC on the basis of the ^14^C determinations presented in this paper.

The successive buildings were of mud-brick framed with oak and suffered (fortunately, from the perspective of the archaeological chronologist) regular destruction as the result of fire, earthquake or natural decay, and the immediate or almost immediate reconstruction on the debris of the preceding phases steadily increased the height of the mound and required, in consequence, the raising of the perimeter banks. Within the settlement, it was possible to distinguish between interiors which were kept more or less clean, unroofed yards where rubbish was allowed to accumulate and streets where gravel and broken pottery was regularly strewn in order to maintain a firm surface in all weathers.

Excavation was directed from 1975–1989 by K.A. Wardle on behalf of the University of Birmingham and the British School at Athens. Ten separate phases of occupation (Phases 9–2, 1.5 and 1, see Figs. S3–S5 in File S1) have been explored in the centre of the mound to reach a depth of c 4.0 m from the surface. Earlier levels have been tested at the edge of the mound but could not be continued further in for practical reasons. A series of preliminary reports and studies of different aspects of the discoveries have already been published, while a comprehensive illustrated overview was published in 2007.

The regular fires had left charred timbers *in situ*, as well as charred crop seeds in several granaries. Animal bones of the principal domesticated species were present in every level of occupation debris. Mycenaean pottery occurred in sufficient quantity to permit absolute dates obtained at Assiros to inform southern Greek Late Bronze Age chronologies, whilst a distinctive Proto-Geometric amphora enables precise dating of the beginning of the Greek Early Iron Age for the first time.

### Permits, sample identifiers and sample location

All necessary permits for excavation and permission to export samples for 14C determinations were obtained from the Greek Archaeological Service for the described study, which complied with all relevant regulations.

The index numbers of the archaeological samples which provide the basis for the determinations discussed in this paper are tabulated in Section A in File S1: the Ox-A numbers, analytical data, identification and context information for the animal bones in Table S1 in File S1; the sample numbers and context information for Dendrochronology and Dendrochronological wiggle-matching in Tables S2 and S3 in File S1; for crop seeds and their contexts in Table S4 in File S1.

All archaeological materials from the excavation at Assiros are housed in the Archaeological Museum of Thessaloniki, Greece (GR 54013). Dendrochronological samples are kept for reference in the Malcolm and Carolyn Wiener Laboratory for Aegean and Near Eastern Dendrochronology at Cornell University, Ithaca (NY 14853). ^14^C samples for dendrochronological wiggle-matching and of crop seeds sent to Heidelberg for high precision determinations have been consumed in the analytical process (other crop seeds from the same contexts are stored in Thessaloniki). Animal bones used partially for sampling are currently held at ORAU Oxford, UK (OX1 3QY) pending eventual return to the Archaeological Museum of Thessaloniki.

### The charred building timbers: Tables S2 and S3 in File S1

**Figure 3:**
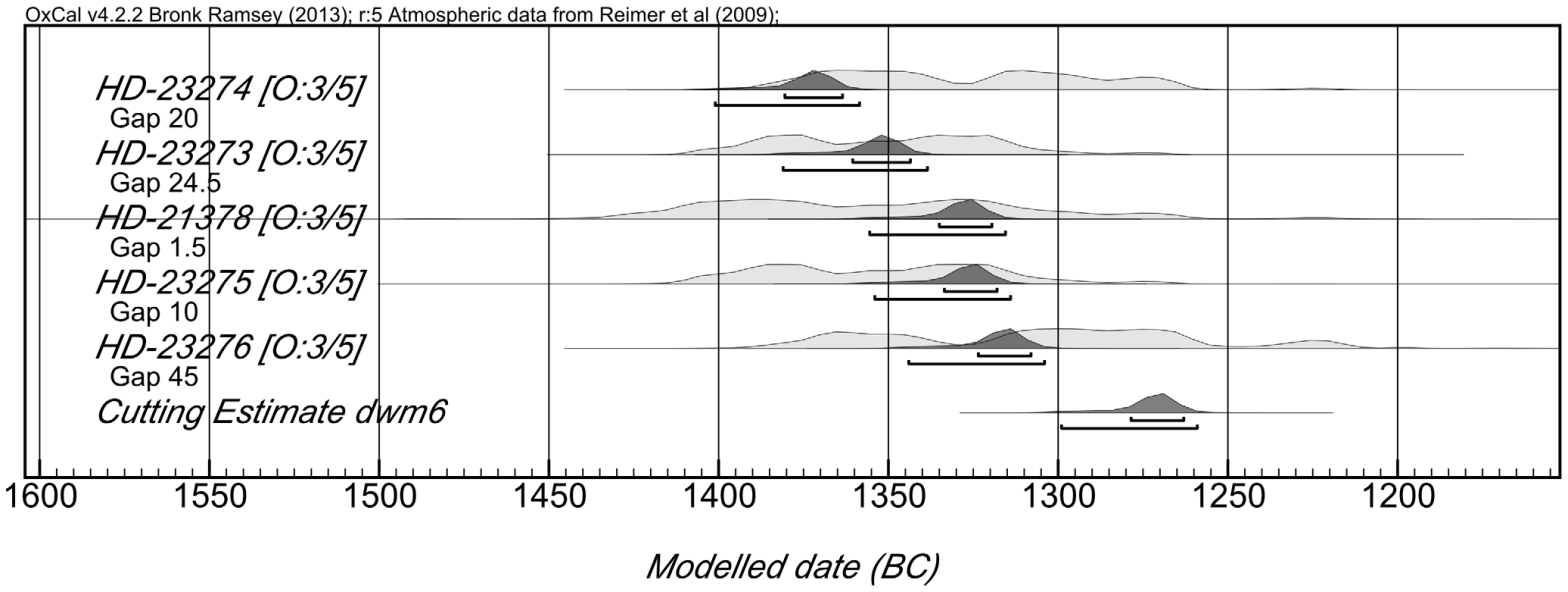
Dendro wiggle match diagram for Phase 6 timbers.

**Figure 4:**
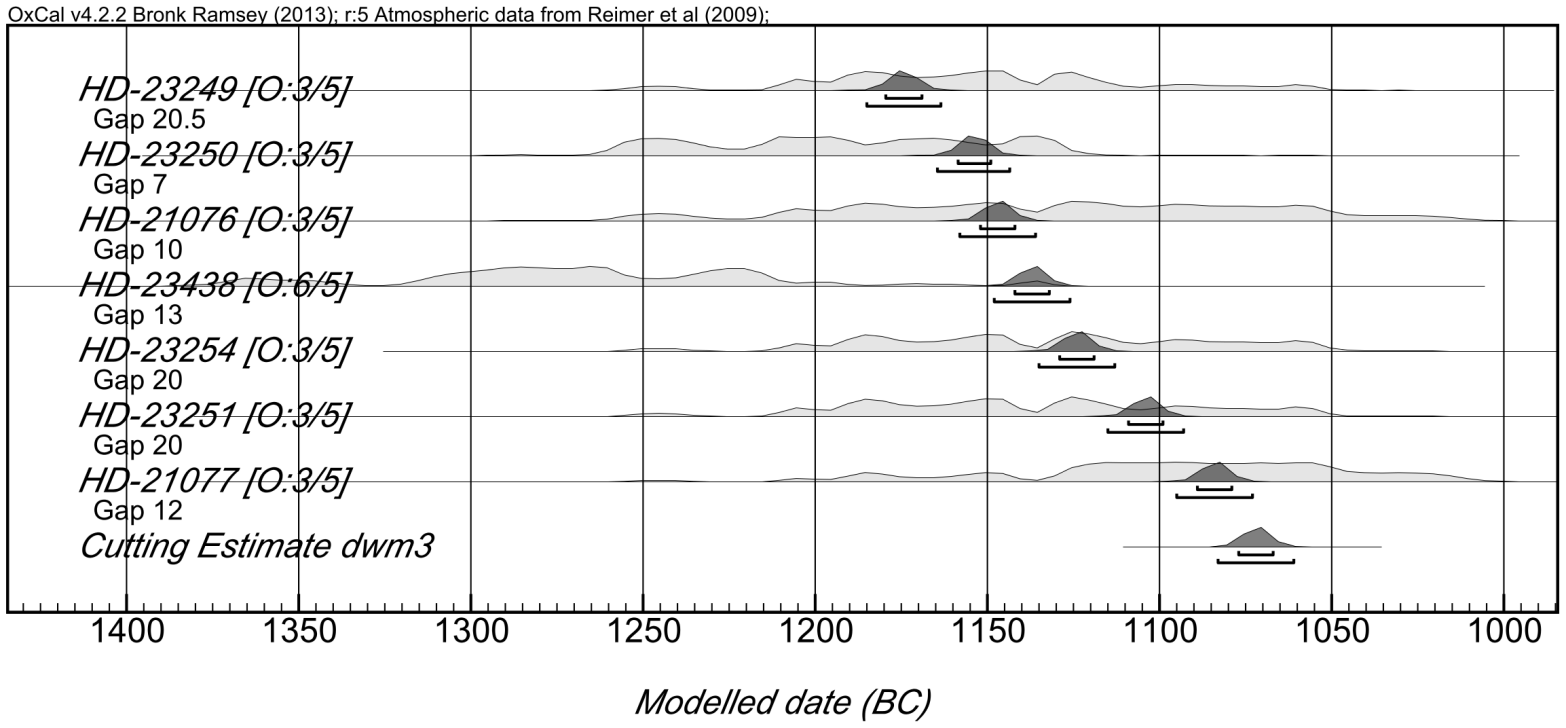
Dendro wiggle match diagram for Phase 3/2 timbers.

Charred timbers were preserved in the earliest level explored (Phase 9) and two ^14^C determinations taken at a 40 year interval on a single timber with waney edge preserved gave a cutting date of 1385–1347 BC (2σ, 95.4% probability) (Fig. S6 in File S1). Three timbers found in the destruction level of Phase 6 were shown, using dendrochronology, to have been growing at the same time and one retained traces of bark. A sequence of five ^14^C determinations covering a span of 50 years gave a cutting date of 1300–1260 BC once DWM was applied (Fig. 3). Four timbers growing at the same time and used in Phases 3 and 2 had been felled as two pairs ten years apart. Two retained their original circular cross section and are most unlikely to have been trimmed beyond the bark layer. A sequence of seven ^14^C determinations covering a span of 90 years gave a cutting date of 1083–1062 BC for those used in Phase 2 [13] (Fig. 4). With all these determinations, we can be confident that the ring sequence from which the individual samples were taken reflects the full life span of the tree concerned and that the dates established are true cutting dates.

In addition, dates obtained for the 104 year ring sequence of the samples from Phases 3 and 2, using dendrochronology cross-matching against the Anatolian master sequence, provided close agreement with the ^14^C dates but increased confidence depends on establishing Aegean oak-based sequences rather than Anatolian juniper-based ones [14].

All of these dates are between 100 and 70 years earlier than anticipated in terms of the accepted chronology. The archaeological evidence, especially in the case of the Phase 3 and Phase 2 samples, makes it unlikely that these are all reused timbers. The complete dating sequence, obtained by Bayesian modelling and including the animal bone determinations undertaken in 2011, enables us to rule out this possibility.

### The charred crop seeds: Table S4 in File S1

The granaries of Phase 9 yielded a rich harvest of different crop seeds – einkorn, emmer and spelt wheat, barley, vetch and millet. Given the likelihood that these had recently been harvested and probably originated from a single year's harvest, seven discrete samples of four species were analysed to clarify the cutting dates of the associated timbers. The tight series of determinations provided an earliest date for the destruction, in which they were charred, of 1378–1343 and a latest of 1370–1334 BC. We measured the span of time represented by the samples using OxCal's interval command and determined that it corresponded to a period of 0–28 years (at 2σ, 95.4% probability) (Table S4 in File S1). The same procedure has been used wherever an interval has been calculated.

### The animal bones and the Oxford Laboratory methods

42 animal bones (*Sus, Bos* and *Ovis*) were prepared for AMS dating at the ORAU, and 36 were successfully dated. All phases (apart from Phase 9), were represented by three or more samples. They were selected from occupation levels in every area of the site on the basis of their large size and the lack of post-discard gnawing. They had therefore been deposited while ‘fresh’ and are likely to be from animals raised for meat and under 5 years old.

Treatment was undertaken using the methods outlined by Brock *et al* [15]. Bone samples were dated with an additional ultrafiltration treatment using a pre-cleaned 30kD MWCO ultra-filter manufactured by Vivaspin™. The recovered filtered gelatin was freeze-dried ready for combustion in a CHN analyser. The sample CO_2_ was reduced over an iron catalyst in an excess H_2_ atmosphere at 560°C prior to AMS radiocarbon measurement using the ORAU 2.5MV HVEE accelerator. Radiocarbon dates of bone and their context information are reported in Table S1 in File S1. All bones were very well preserved in terms of collagen, with only one <than 1% wt. collagen (the effective threshold in the ORAU). All other analytical parameters measured, including the carbon to nitrogen atomic ratio, were acceptable.

**Figure 5:**
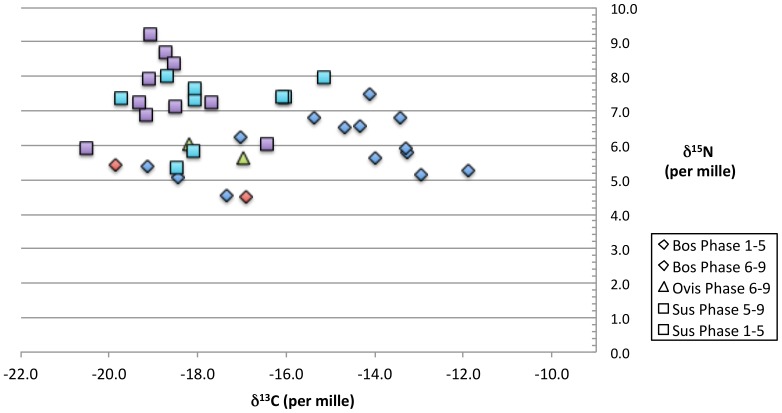
Stable isotope (C and N) values from Assiros. The values are divided between Phases 9–6 and 5–1 (see Table S1 in File S1 for values). Most cattle (*Bos*) from Phase 5 and later ate an atypical diet with high levels of C-4 plants. Errors for the isotope measurements are ±0.2 for Carbon and ±0.3 for Nitrogen. These are not shown on the figure.

δ^15^N and δ^13^C isotope values obtained during the preparation for ^14^C determinations showed that the pigs and sheep and some of the cattle were consuming a normal C3 plant-based diet. The cattle from later levels (Phase 5 onwards), however, were almost all consuming plants derived via a C4 rather than the normal C3 pathway (Fig. 5) but the δ^15^N values for these rule out grazing in a salt-marsh environment which was suggested for the animals sampled from the contemporary site of Kastanas 35 km away, and used *inter alia* to explain the unexpectedly ‘old’ dates at that site [16]. The most likely explanation seems to us to be that cattle at this period were being stall-fed with a crop such as millet, which is a C4 plant, on a very regular basis, since the natural grazing in the region could not have produced this effect. There is, moreover, no systematic discrepancy between the dates for the cattle which were eating a C4 plant diet and for the pigs in the same strata which were not, nor any evidence for reservoir offsets. Assiros is some 30 km from the nearest coast and salt-marsh grazing is, in any case, extremely unlikely.

## Method

### The stratigraphic sequence, the Bayesian model and absolute dates

The stratigraphic sequence at Assiros was continuous, at least from Phases 10–1.5. Each phase was defined as a closed sequence of construction, use and destruction and, as far as can be determined, reconstruction took place shortly after destruction in almost every instance. The only clear hiatus occurred between the end of Phase 1.5 and the start of Phase 1. Phase 9 (Fig. S3 in File S1), the earliest from which samples were available, is dated absolutely by construction timbers, by animal bones from its use and by crop seeds from its destruction fire. Phases 8 and 7 are dated by the animal bones from the periods of use. Phase 6 (Fig. S4 in File S1) is dated by construction timbers, by animal bones from its use and by a single sample of crop seeds from its destruction fire. Phases 5 and 4 are dated by animal bones from the period of use, whilst Phases 3 and 2 (Figs. S5 & S8 in File S1) are dated by both construction timbers and by animal bones from their use. No samples have been analysed from Phase 1.5, a short period of reoccupation after a fire, and the only samples from Phase 1 are bones. It is not possible to determine whether the animal bones date to the initial stage of use of any structure or indeed to its final phase of use. Given that the building phases at Assiros in most cases are of short duration this uncertainty normally falls within the range of the radiocarbon determinations.

**Figure 6:**
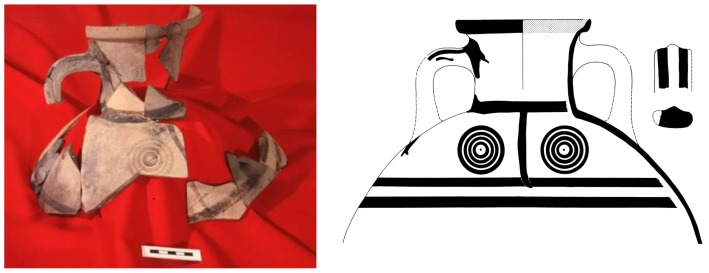
Assiros Proto-Geometric amphora from Phase 3. For find spots see Fig. S8 in File S1.

The local Macedonian pottery, which forms more than 90% of any assemblage, is hand-made and changes only slowly in terms of shape and decoration. The end of the *Macedonian* Bronze Age and start of the Iron Age at Assiros is marked by the introduction in Phase 4 of thinner, harder-fired pottery with rather more angular shapes and a characteristic incised or stamped decoration, but long-established local traditions clearly continue. Mycenaean pottery, which can be related to southern Greek sequences, is found in increasing quantities from Phase 10 onwards, with a maximum presence in Phase 6. At first the pottery was entirely imported to Macedonia but later ‘locally-made’ vessels became quite frequent. Phase 5 has far fewer pieces and no complete vessels, suggesting the possibility that much, if not all, the Mycenaean pottery from that phase is residual. Apart from a few worn Mycenaean sherds, there is no independently datable pottery in Phase 4. A single but highly significant Early Proto-Geometric amphora (Fig. 6) was broken in the Phase 3 destruction fire, whilst the pottery from Phases 2, 1.5 and 1 is local in character and not as yet precisely datable.

The end of the Bronze Age and start of the Iron Age in southern Greece is marked, among other characteristics, by the appearance of Proto-Geometric pottery, but cannot be directly related to the equivalent transition in Macedonia. At each of the sites where extensive excavation has taken place (Assiros, Kastanas and Toumba Thessalonikis) the local repertoire of fabrics and shapes is slightly different and direct comparisons between the assemblages at each of the sites are difficult. At Toumba Thessalonikis the distinctive features that make up the local Iron Age repertoire are introduced gradually from Level 3 onwards and found with Late Helladic IIIC late pottery, whilst the concentric circle decoration on wheel-made pottery, which is the hallmark of the Proto-Geometric style, does not appear until Level 2A [17]. The relative chronologies of key levels at Assiros and Kastanas are set out in Table S10 in File S1 together with those from Toumba Thessalonikis. At Kastanas, where the first local Iron Age pottery is found in a single level (Schicht 12) together with Mycenaean and Proto-Geometric pottery, it cannot be determined whether local EIA pottery came into use during the same period as Mycenaean or at the same time as Proto-Geometric.

At Assiros, as already noted, imported pottery is absent from the relatively long Phase 4 and we cannot, therefore, determine on the basis of the pottery whether this phase at Assiros should be considered contemporary with the final Bronze Age (Late Helladic IIIC late) in southern Greece or with the initial stage of the Iron Age (Early Proto-Geometric). The local EIA pottery style was already well established by the time the EPG amphora, which was shattered in the Phase 3 destruction fire, reached Assiros. The position of this phase in relation to southern Greek sequences is important in considering the impact of the absolute dates obtained on any estimation of the absolute start date of Proto-Geometric (see discussion of results below).

All dated samples, from timbers, crop seeds and animal bones have been included in a Bayesian chronometric model (Section A in File S1, Fig. S2 in File S1, Tables S5 & S11 (the model code) in File S1) based on the stratigraphic sequence information described above.

We used OxCal 4.2.2 [18] and the INTCAL13 calibration curve [19] to achieve this. Bayesian modelling allows the archaeological stratigraphic information to be incorporated in the chronometric modelling, along with the radiocarbon likelihoods [20], [21]. The model framework reflects the series of phases, destruction layers and archaeological strata excavated through the sequence of the Assiros tell. For each model a start and end boundary is included to bracket each archaeological phase throughout the sequence. Two are included where we need to account for the hiatus between Phases 1.5 and 1, otherwise they simply demarcate the separation of one phase from another. The boundary posterior distributions allow us to determine probability distribution functions (PDF) for the beginning and ending of these phases and also to interrogate information about the span of time that has elapsed between them. Within the sequence of phases we incorporated the dating evidence derived from high precision wiggle-matched radiocarbon sequences, dendro-dated calendar estimates, high precision radiocarbon dates of cereal grains and the AMS dates from identified domestic animal bones. The Bayesian model permits us to estimate the duration of each phase (Fig. S1 in File S1). This suggests that each building phase is marked by the passage of one or perhaps two human generations. The full model plot is shown in Fig. S2 in File S1.

We ran the model multiple times at several million iterations to assess reproducibility. Initial runs of the model were either slow or the MCMC stopped. To solve this problem we included several uniform boundaries with specific ranges, reducing the runtime for the algorithm to find a proper fit over a wider temporal range. The posterior data were very reproducible. Low convergence occurs when MCMC algorithms experience problems in calculating the solution, and incompatible solutions mean the algorithm is slow to calculate, or converge. The convergence values for Assiros models were consistently high and averaged 99.5%.

The Bayesian modelling shows that the animal bone determinations are consistent in terms of matching the cutting dates for the timbers from the same building phase (within the standard error values). The timbers must therefore have been freshly cut, not reused. The assertion by Weninger and Jung [22] ‘that the beams do not originally stem from the architectural phases in which they were found stratified’ is therefore entirely unfounded and can be definitively rejected. The modelled sequence permits a series of key transitions to be established.

In consequence, a long sequence of absolute dates has been obtained without any reference to ‘historical’ dates or any attempt to predict the length of any phase to constrain the modelling. In particular, the 36 domestic animal bone samples, formed a consistent sequence with only two outliers [23] (Table S6 in File S1). This pattern is statistically robust and is not the product of bones which had been moved around at random during successive phases of construction.

## Results and Discussion of the Chronological Significance for Southern Greece and the Wider Mediterranean Region

Phase 9, with extensive granaries and imported pottery of the Mycenaean late LH IIIA 2 period, starts between 1395–1346 BC and ends with destruction by fire 1378–1343 BC, a period of 0–19 years (at 2σ, 95.4% probability). Phase 7, with LH IIIC style pottery which should correspond to the period immediately after the destruction of palatial Mycenaean society in southern Greece, starts 1341–1282 BC and ends 1312–1264 BC with a span of 0–46 years (at 2σ, 95.4%). Phase 3, the second phase of the Iron Age at Assiros including a Proto-Geometric amphora (Fig. 6) starts 1096–1074 BC and ends 1087–1064 BC with an overall span of time of 0–15 years (at 2σ, 95.4%). Each of these dates is considerably older than expected on conventional grounds as shown in Table 1. There is no argument for rejecting this sequence in terms of old wood, old bones or special diets.

**Table 1:** Modelled dates from Assiros: offsets from conventional dates.

Phase	Pottery Period	Expected/conventional date	Absolute/^14^C date BC (at 2σ, 95.4%)	
			Start	End
1		?? 750–650	1072–1024	1067–1004
Hiatus				
2		950–900	1081–1056	1072–1024
3	Early Proto-Geometric	1000–950	1096–1074	1087–1065
4	**	1050–1000	1232–1145	1140–1078
5	Late Helladic IIIC	1100–1050	1265–1203	1232–1145
6	Late Helladic IIIC	1150–1100	1300–1253	1265–1203
7	Late Helladic IIIC	1200–1150	1341–1282	1312–1264
8	Late Helladic IIIB	1300–1200	1370–1334	1341–1282
9	Late Helladic IIIA2	1350–1300	1395–1346	1378–1343

**See discussion above for the position of Assiros Phase 4 relative to southern Greek sequences.

Given the clear sequence of absolute dates from Assiros and their importance, our next step was to compare them with the determinations obtained from Kastanas which have been used to support the conventional chronology (Section D in File S1, Figs.S9 & S10 in File S1). We reassessed both the stratigraphic evidence and the pattern of dates with a similar Bayesian model without any input from historical dates or hypothetical phase durations (Figs. S11 & S12 in File S1, Tables S9 & S12 (the model code) in File S1). The sequences resulting from modelling the ^14^C data sets at both Assiros and Kastanas are reasonably consistent with each other, allowing for the difficulties in matching, through ceramic parallels, building levels at two sites excavated with different methods. Previous attempts to reject the self-evident offset at Kastanas from the expected historical dates rely on special pleading about the character of the Kastanas sample base rather than firm evidence [16] and, moreover, ignore some of the pottery evidence from each stratum (Section D in File S1, Tables S7 & S8 in File S1).

The ceramic parallels from Assiros provide relative chronological links between the ceramic phases in northern and southern Greece. In consequence, the absolute dates from the Assiros contexts can be transferred to equivalent contexts in southern Greece, and used to re-date the successive pottery phases there. Their significance is most readily seen in respect of the start date for the Proto-Geometric period, for which there is no evidence-based historical chronology but rather a ‘best guess’ of 1050 or 1025 BC. This has been determined on the hypothetical duration of the pottery styles of the final Mycenaean period and on the occurrence of Proto-Geometric pottery in the coastal regions of Syria, Lebanon and Israel (Fig. S7 in File S1). There is, however, no agreement about the dating of the levels where this pottery has been found but rather a vigorous debate about the dates in relation to Biblical history. The discussion of a high or low chronology in this region has recently had particular prominence [10], [11]. Indeed the presence of Proto-Geometric pottery has often been used to support dates in both schemes without regard for circularity of argument.

The Assiros Proto-Geometric amphora (Fig. 6) belongs to a category discovered at other Macedonian sites, at Troy, in central Greece and at Lefkandi on Euboea (Fig. S7 in File S1). This category is generally assumed to have derived from Attic prototypes after the passage of some years [24]. There is surely no doubt that the Proto-Geometric style had evolved before the amphora at Assiros was manufactured, unless the style originated with this vessel, which would be surprising. Our model provides an estimate for the age of the amphora in Phase 3, and consequently a minimum age for the start of the Proto-Geometric style, of 1095–1070 BC (at 2σ, 95.4% probability). Given this estimate for the Assiros example, it should follow that Attic Proto-Geometric, as the archetype of the whole style, originated before then, perhaps by several decades. This might indicate a date nearer 1100 BC for the origin of the style if, as is normally accepted, the style was first developed in Athens and was then spread by exports and imitation to the northern Aegean and to Cyprus and the Near East to reach that region at approximately the same time as it reached Macedonia.

Although Weninger and Jung question the attribution of the Proto-Geometric vessel to Assiros Phase 3 and accordingly the date deduced for its manufacture, the majority of the fragments came from Phase 3 floors and a single piece was incorporated into a wall when the reconstruction defined as Phase 2 took place (Fig. S8 in File S1). The assertion by Weninger and Jung that these circumstances result from complex reverse taphonomic processes is therefore untenable [22]. Since the amphora must have been broken in the Phase 3 destruction, before the Phase 2 structures were erected, the *terminus ante quem* of 1083–1062 BC for its manufacture, given by the DMW date for the Phase 2 construction timbers, remains valid.

The absolute date for the start of the preceding Phase 4 (Fig. S13 in File S1), which has some of the deepest deposits of occupation debris at the site, cannot be directly related to the southern Greek sequences since the level contains neither imports nor imitations of southern Greek wheel-made pottery (Mycenaean or Proto-Geometric) as noted above. There is, as yet, no equivalent absolute date from a southern Greek site to which it (or, indeed, any of the other absolute dates from Assiros) can be correlated. If, as seems most probable, Assiros Phase 4 is contemporary with the final Bronze Age of southern Greece, then the absolute date for the start of Early Proto-Geometric should be based on the date obtained for Phase 3, ie earlier than c 1080 BC. If, however, Phase 4 is contemporary with the early stages of Proto-Geometric, an earlier, perhaps much earlier, start date would be indicated, at least c 1120 BC.

The date for the introduction of Proto-Geometric derived from the finds at Assiros fits well with the high Levantine chronology whilst the conventional duration of Attic Proto-Geometric between 1025 and 900 BC, is regularly used as support for the low chronology. Although numerous ^14^C samples from Israel have now been processed with the goal of establishing an absolute chronology for that region, their significance is hotly debated [10], [11].

We learnt, in the final stages of preparation of this paper, of 15 new radiocarbon determinations from Lefkandi, Kalapodi and Corinth [25] which have been modelled using Bayesian analysis and purport to show that they are compatible with the conventional ‘low’ chronology for LH IIIC - Proto-Geometric. There are, however, several grounds for not accepting this conclusion which we will explore in detail in a later paper. In summary: a) only a small number of samples have been measured from each of the three sites; b) these samples are related stylistically not stratigraphically and it would therefore have been a better approach to model the data from each site separately before combining the results; c) the error margins of the determinations are around 50 yrs +/-, and the 95.4% probability spans range from 117 yrs to 352 yrs with a mean of 251 yrs; d) some of the samples show such poor agreement that the authors have ‘moved’ two samples to where they fit better; e) they reject the dates provided by ‘only two’ samples for the early stages of Geometric as too early but accept the single ‘late’ date for the Early – Middle stage of LH IIIC without comment. Close examination of their data suggests that their assertion that the results support the conventional chronology for the LH IIIC to Proto-Geometric periods is over-optimistic.

## Conclusions

Although the rationale of the conventional dates currently used for the later phases of the Greek Bronze Age has been set out in detail by Warren and Hankey [26], Weninger and Jung [16] and others, the fact remains that the dates from Assiros and Kastanas are systematically offset from these to approximately the same value as those from Thera at the beginning of the Late Bronze Age. It may reasonably be asked how these discrepancies arise. Can the Assiros dates be reconciled with historical dates by careful re-examination of the links between different areas at different periods? If not, which chronology should be preferred and why? To give priority to the historical dates is to challenge 60 years of research into, and improvement of, the ^14^C methodology and the development of a series of accurate calibration curves. To give priority to the ^14^C dates calls into question much of the conventional historical chronology for this period in the Eastern Mediterranean.

Recent studies of the dating of the Egyptian Old to New Kingdoms have demonstrated that historical dates and ^14^C-derived dates are compatible where reliable samples are selected and the correct methodologies applied [2]–[4]. The consistency of the results relating to the Thera eruption demonstrates the importance of a range of different sample types, although ideally they would have had chronological depth as well as geographical breadth. The exceptionally robust character of the Assiros sequence is based on 1) the length of the stratified sequence, 2) the number and variety of samples, 3) the accuracy of DWM as applied to building timbers and 4) on the confirmation from the animal bones that the timbers are not reused. It thus provides a series of anchor points for future ^14^C and dendrochronological studies in the Aegean area and challenges long-established assumptions about historical chronology in the region.

## Supporting Information

File S1 **Supporting figures and tables. Figure S1.** Intervals calculated for each Assiros phase. **Figure S2.** Bayesian model for Assiros; *left* Phases 9–6, *right* Phases 5–1. **Figure S3.** Assiros Phase 9 with granaries. **Figure S4.** Assiros Phase 6 plan. **Figure S5.** Plan of village in Phase 2. **Figure S6.** Dendro wiggle match diagram for Phase 9 timber. **Figure S7.** *Left:* Distribution of Group 1 Proto-Geometric amphorae *after* Catling 1998, 156. *Right:* Distribution of Proto-Geometric pottery in the Levant *after* Lemos 2002, 229, Map 8. **Figure S8.** Find spots diagram for pieces of the PG amphora (green) and the 4 timbers used for dendrochronology and the DWM (red). **Figure S9.** In the Kastanas plot preferred by Weninger and Jung, the determinations are given their expected historical ages – resulting in a systematic offset from the calibration curve of around 100 years. **Figure S10.** In the Kastanas plot rejected by Weninger and Jung, the dates fit with reasonable conformity to the calibration curve. **Figure S11.** Bayesian model of Kastanas ^14^C data, without prior assumptions about period length or assumed ‘historical’ date. **Figure S12.** Kastanas: The start and end dates for stratum/Schicht 14b as derived from the Bayesian model. **Figure S13.** The start dates of Assiros Phase 4 and Kastanas Schicht 12 compared. **Table S1.** Radiocarbon AMS dates from Assiros obtained from bones with associated analytical and context data. **Table S2.** Oak timbers from Assiros used for dendrochronology. **Table S3.** Oak timbers from Assiros used for dendrochronological wiggle-matching (DWM). **Table S4.** Crop seeds from Assiros Phase 9 Granary Room 9, and pithos in Phase 6 Room 20. **Table S5.** Assiros: Results of the Bayesian modelling. **Table S6.** Assiros: Results of the Bayesian outlier modelling. **Table S7.** Kastanas: summary of date on basis of Jung's rejection of the LH IIIC Late parallels for catalogue number 91. **Table S8.** Kastanas: summary of date if LH IIIC Late parallels for catalogue number 91 are accepted. **Table S9.** Kastanas: The ^14^C derived date ranges for the samples from each stratum/Schicht plotted against the destruction dates for each level. **Table S10.** The relative chronology of key building levels at Assiros, Kastanas and Toumba Thessalonikis. **Table S11.** Appendix 1: Model code for the Bayesian age model of Assiros. **Table S12.** Appendix 2: Model code for the Bayesian age model of Kastanas.
(PDF)

## References

[1] Cherubini P, Humbel T, Beeckman H, Gärtner H, Mannes D, et al (2014) Bronze Age catastrophe and modern controversy: dating the Santorini eruption. Antiquity 88: 267–291.

[2] Friedrich WL, Kromer B, Friedrich M, Heinemeier J, Pfeiffer T, et al (2006) Santorini Eruption Radiocarbon Dated to 1627–1600 BC. Science 312 548. 10.1126/science.1125087 16645088

[3] Manning SW, Bronk Ramsey C, Kutschera W, Higham T, Kromer B, et al (2006) Chronology for the Aegean Late Bronze Age 1700–1400 BC. Science 312: 565–569. 16645092 10.1126/science.1125682

[4] Bronk Ramsey C, Dee MW, Rowland J, Higham T, Harris SA, et al (2010) Radiocarbon-Based Chronology for Dynastic Egypt. Science 328: 1554–1557. 20558717 10.1126/science.1189395

[5] Dee MW, Brock F, Harris SA, Bronk Ramsey C, Shortland AJ, et al (2010) Investigating the likelihood of a reservoir offset in the radiocarbon record for ancient Egypt. Journal of Archaeological Science 37: 687–693.

[6] Voutsaki S, Nijboer AJ, Philippa-Touchais A, Touchais G, Triantaphyllou S (2006) Analyses of Middle Helladic Skeletal Material from Aspis, Argos. Bulletin de Correspondance Hellénique 130: 613–625.

[7] Wild EM, Gauß W, Forstenpointner G, Lindblom M, Smetana R, et al (2010) ^14^C dating of the Early to Late Bronze Age stratigraphic sequence of Aegina Kolonna, Greece. Nuclear Instruments and Methods in Physics Research B 268: 1013–1021.

[8] Manning SW, Kromer B (2011) Radiocarbon dating archaeological samples in the Eastern Mediterranean, 1730 to 1480 BC: further exploring the atmospheric radiocarbon calibration record and the archaeological implications. Archaeometry 2: 413–439.

[9] Wardle KA, Wardle D (2007) Assiros Toumba: A brief history of the settlement, in The Struma/Strymon River Valley in Prehistory. Proceedings of the International Symposium Strymon Praehistoricus, Kjustendil–Blagoevgrad (Bulgaria), Serres–Amphipolis (Greece) 27.09–01.10.2004. (In The Steps of James Harvey Gaul Volume 2) eds. Todorova H, Stefanovich M, Ivanov G, Sofia, 451–479.

[10] Bruins HJ, Nijboer A, Van der Plicht J (2011) Iron Age Mediterranean Chronology: A Reply. Radiocarbon 53 ((1)) 199–220.

[11] Fantalkin A, Finkelstein I, Piasetzky E (2011) Iron Age Mediterranean Chronology: A Rejoinder. Radiocarbon 53 ((1)) 179–198.

[12] Alberti G (2013) Issues in the absolute chronology of the Early-Middle Bronze Age transition in Sicily and southern Italy: a Bayesian radiocarbon view. Journal of Quaternary Science 28 ((6)) 630–640.

[13] Newton MW, Wardle KA, Kuniholm PI (2005) A Dendrochronological ^14^C Wiggle-Match for the Early Iron Age of north Greece: A contribution to the debate about this period in the Southern Levant, in Levy and Higham eds. The Bible and Radiocarbon Dating: Archaeology, Text and Science, 104–113.

[14] Newton MW, Wardle KA, Kuniholm PI (2005) (2003) Dendrochronology and Radiocarbon determinations from Assiros and the beginning of the Greek Iron Age. Archaiologikon Ergon Makedonias kai Thrakis 17 173–190.

[15] Brock F, Higham T, Ditchfield P, Bronk Ramsey C (2010) Current Pretreatment Methods for AMS Radiocarbon Dating at the Oxford Radiocarbon Accelerator Unit (ORAU). Radiocarbon 52 ((1)) 103–112.

[16] Weninger B, Jung R (2009) Absolute chronology of the end of the Aegean Bronze Age, LH IIIC Chronology and Synchronisms III: LH IIIC Late and the Transition to the Early Iron Age. Proceedings of the International Workshop at the Austrian Academy of Sciences at Vienna, February 23rd and 24th, 2007, Veröffentlichungen der Mykenischen Kommission, Band 30, eds. Deger-Jalkotzy S. and Baechle AE, Vienna, 373–416.

[17] Andreou S (2014) Personal communication.

[18] Bronk Ramsey C (2001) Development of the radiocarbon calibration program OxCal. Radiocarbon 43: 355–363.

[19] Reimer PJ, Bard E, Bayliss A, Beck JW, Blackwell PG, et al (2013) IntCal13 and Marine13 Radiocarbon Age Calibration Curves 0–50,000 Years cal BP. Radiocarbon 55 ((4)) 1869–1887.

[20] Bronk Ramsey C (2009) Bayesian analysis of radiocarbon dates. Radiocarbon 51 ((1)) 337–360.

[21] Buck CE, Cavanagh WG, Litton CD (1996) Bayesian approach to interpreting archaeological data. John Wiley and Sons, Chichester.

[22] Weninger B, Jung R (2009) Absolute chronology of the end of the Aegean Bronze Age, LH IIIC Chronology and Synchronisms III: LH IIIC Late and the Transition to the Early Iron Age. Proceedings of the International Workshop at the Austrian Academy of Sciences at Vienna, February 23rd and 24th, 2007, Veröffentlichungen der Mykenischen Kommission, Band 30, eds. Deger-Jalkotzy S. and Baechle AE, Vienna, 388.

[23] Bronk Ramsey C (2009) Dealing with outliers and offsets in radiocarbon dating. Radiocarbon 51 ((3)) 1023–1045.

[24] Catling RWV (1998) The Typology of the Protogeometric and Subgeometric pottery from Troia and its Aegean context. Studia Troica 8: 153–164.

[25] Toffolo MB, Fantalkin A, Lemos IS, Felsch RCS, Niemeier W-D, et al. (2013) Towards an Absolute Chronology for the Aegean Iron Age: New Radiocarbon Dates from Lefkandi, Kalapodi and Corinth, www.plosone.org/article/authors/info%3Adoi%2F10.1371%2Fjournal.pone.0083117; 10.1371/journal.pone.0083117 PMC3873300 24386150

[26] Warren PM, Hankey V (1989) Aegean Bronze Age Chronology, Bristol.

