# Supplementary material for: Dating the End of the Greek Bronze Age: A Robust Radiocarbon-Based Chronology from Assiros Toumba
Source: PLoS One. 2014 Sep 15;9(9):e106672. doi: 10.1371/journal.pone.0106672 (PMC4164355; doi:10.1371/journal.pone.0106672)
Supplement: File S1 — Supporting figures and tables. Figure S1. Intervals calculated for each Assiros phase. Figure S2. Bayesian model for Assiros; left Phases 9–6, right Phases 5–1. Figure S3. Assiros Phase 9 with granaries. Figure S4. Assiros Phase 6 plan. Figure S5. Plan of village in Phase 2. Figure S6. Dendro wiggle match diagram for Phase 9 timber. Figure S7. Left: Distribution of Group 1 Proto-Geometric amphorae after Catling 1998, 156. Right: Distribution of Proto-Geometric pottery in the Levant after Lemos 2002, 229, Map 8. Figure S8. Find spots diagram for pieces of the PG amphora (green) and the 4 timbers used for dendrochronology and the DWM (red). Figure S9. In the Kastanas plot preferred by Weninger and Jung, the determinations are given their expected historical ages – resulting in a systematic offset from the calibration curve of around 100 years. Figure S10. In the Kastanas plot rejected by Weninger and Jung, the dates fit with reasonable conformity to the calibration curve. Figure S11. Bayesian model of Kastanas 14C data, without prior assumptions about period length or assumed ‘historical’ date. Figure S12. Kastanas: The start and end dates for stratum/Schicht 14b as derived from the Bayesian model. Figure S13. The start dates of Assiros Phase 4 and Kastanas Schicht 12 compared. Table S1. Radiocarbon AMS dates from Assiros obtained from bones with associated analytical and context data. Table S2. Oak timbers from Assiros used for dendrochronology. Table S3. Oak timbers from Assiros used for dendrochronological wiggle-matching (DWM). Table S4. Crop seeds from Assiros Phase 9 Granary Room 9, and pithos in Phase 6 Room 20. Table S5. Assiros: Results of the Bayesian modelling. Table S6. Assiros: Results of the Bayesian outlier modelling. Table S7. Kastanas: summary of date on basis of Jung's rejection of the LH IIIC Late parallels for catalogue number 91. Table S8. Kastanas: summary of date if LH IIIC Late parallels for catalogue number 91 are accepted. Table S9. Kastanas: [file pone.0106672.s001.pdf]

Supporting information for

# Dating the end of the Greek Bronze Age: a robust radiocarbon-based chronology from Assiros Toumba

K.A. Wardle,* T. Higham, B. Kromer,

*Corresponding author.

E-mail: k.a.wardle@bham.ac.uk

## Section A: Radiocarbon dating methodologies, determinations and modelling

The radiocarbon dates from Assiros have been obtained from two laboratories; the Oxford Radiocarbon Accelerator Unit (ORAU) at the University of Oxford and the Radiocarbon Laboratory at the University of Heidelberg.

### *Oxford Laboratory methods used for treatment of bone*

The ORAU undertook radiocarbon dating of bone. Treatment was undertaken using the methods outlined by Brock *et al*.[1]

Combusted gelatin samples were analysed using a Europa Scientific ANCA-MS system consisting of a 20-20 IR mass spectrometer interfaced to a Roboprep CHN sample converter unit operating in continuous flow mode using an He carrier gas. This enables the stepwise measurement of $\delta^{15}N$ and $\delta^{13}C$, nitrogen and carbon content and calculation of C:N atomic ratios. $\delta^{13}C$ values for radiocarbon measurements cited in this section are reported with reference to VPDB.

Radiocarbon dates of bone and their context information are reported in Table S1. All bones were very well preserved in terms of collagen, with only one <than 1% wt. collagen (the effective threshold in the ORAU). All other analytical parameters measured, including the carbon to nitrogen atomic ratio, were acceptable.

*Heidelberg Laboratory methods used for seeds*

In the Heidelberg laboratory the seed samples were pre-treated using the ABA sequence, i.e. they were kept for 1 hour at 80° in a 4% HCl solution, followed by 4% NaOH at room temperature overnight, and 1 hour at 80° in a 4% HCl solution. This procedure resulted in a loss of between 20 and 70% of the sample (average 43%). The samples were combusted in a modified deVries combustion system. $CO_2$ was purified over activated charcoal. The samples were counted in low-level proportional counters for up to 10 days. The measurements were evaluated following Kromer and Münnich.[2]

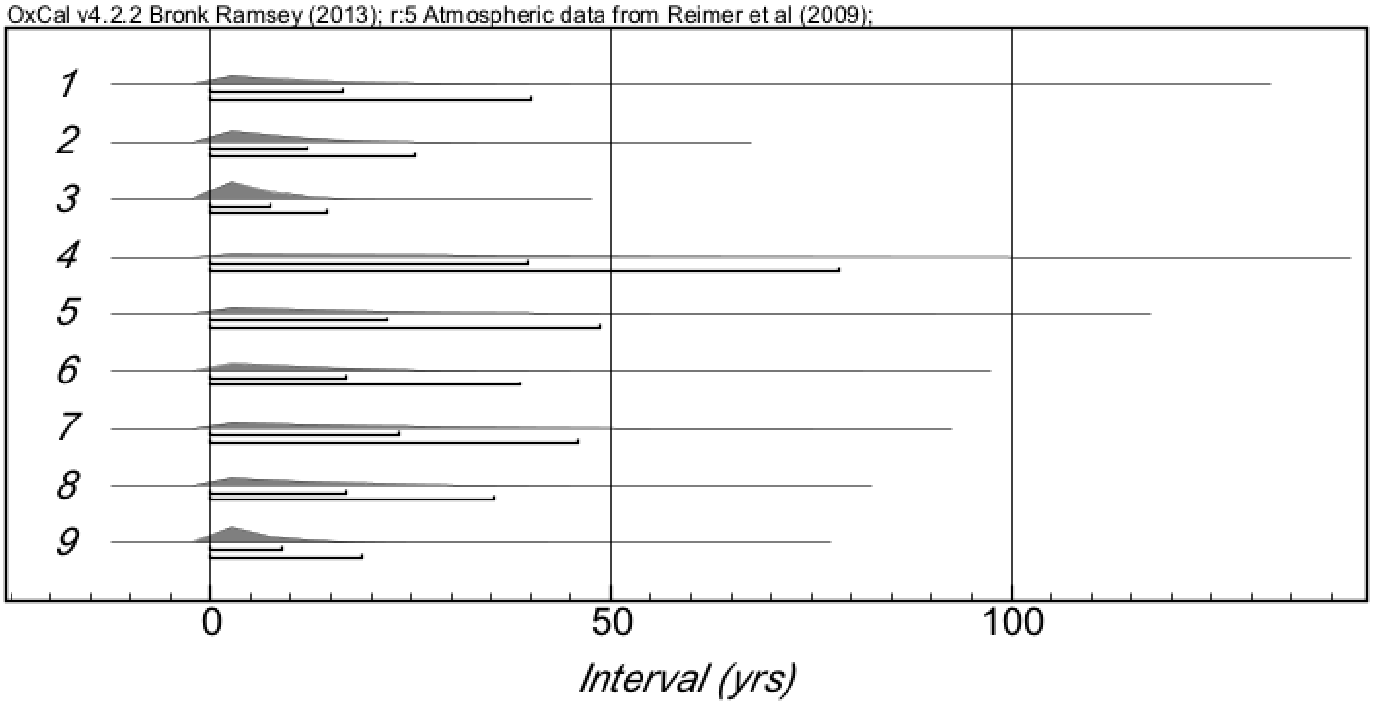


**Figure S1: Intervals calculated for each Assiros phase.**

These reflect the overall span for each of the successive phases, as determined by the Bayesian model *without* the incorporation of any assumptions about the length of each phase on the basis of depth or character of the deposits.

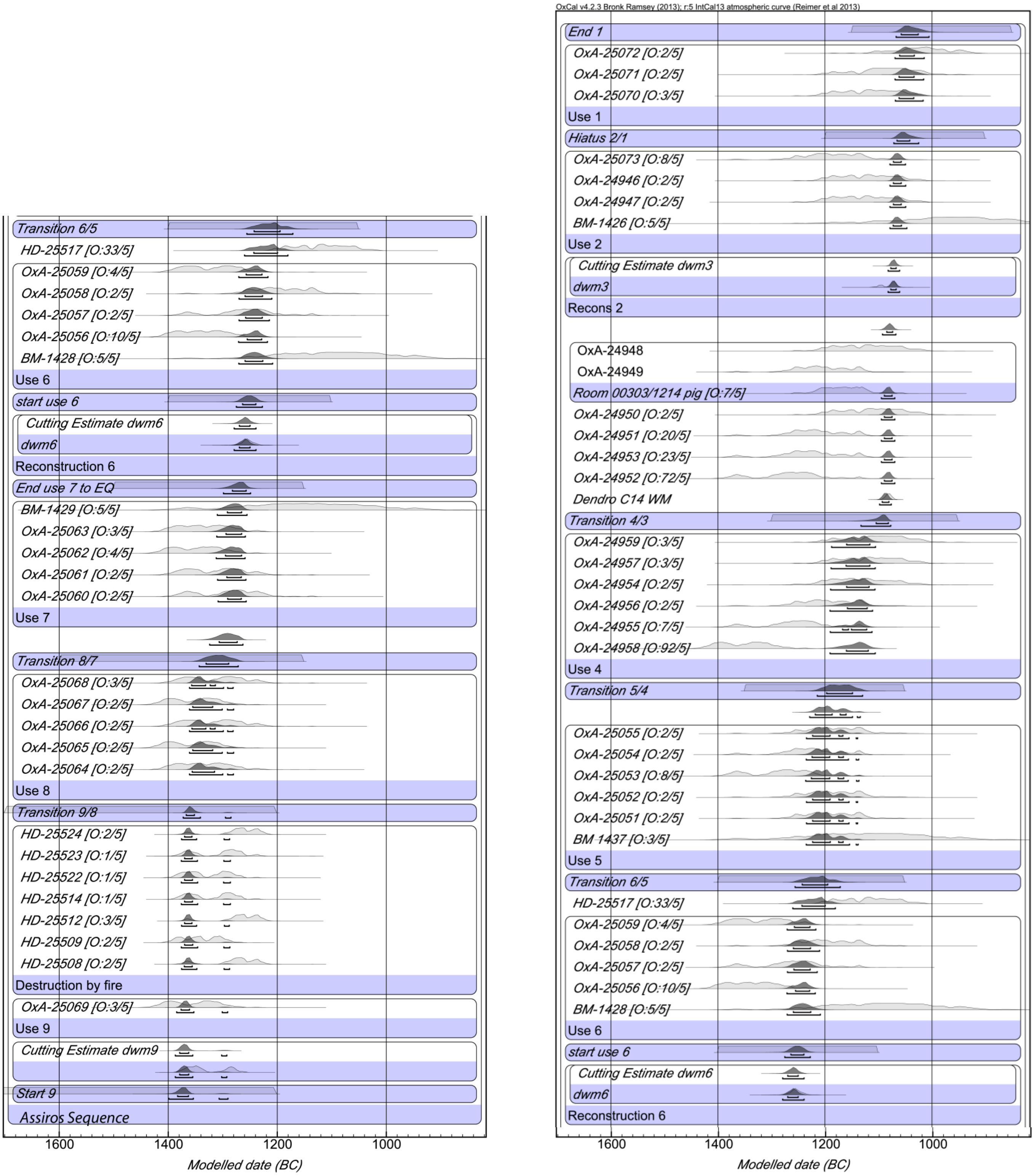


**Figure S2: Bayesian model for Assiros: *left* Phases 9-6, *right* Phases 5-1**

see Table S11, appendix 1 for the model code

and www.artsweb.bham.ac.uk/aha/kaw/assiros/plos/assirosmodel.jpg for the unified plot

**Table S1: Radiocarbon AMS dates from Assiros obtained from bones, associated analytical data and context information**.

With one exception, collagen yields are greater than 1% weight (the ORAU threshold limit) and all C/N atomic ratios are within the acceptable range of 2.9-3.5 employed at the Oxford laboratory. All other measurement parameters are acceptable.

| OxA- | Radio carbon age BP | +/- | Species/ element | Used (mg) | Yield (mg) | %Yld | %C | $\delta^{13}C$ (‰) | $\delta^{15}N$ (‰) | C:N atomic ratio | Zembil/ context number | Phase | Room/ area | Context type |
|---|---|---|---|---|---|---|---|---|---|---|---|---|---|---|
| 24946 | 2924 | 29 | *Bos* humerus r | 640 | 54.57 | 8.5 | 42.3 | -11.9 | 5.3 | 3.2 | 16034 | 2 | 22 | street |
| 24947 | 2928 | 30 | *Bos* humerus l | 630 | 76.14 | 12.1 | 44.1 | -14.1 | 7.5 | 3.2 | 32018 | 2 | 22 | street |
| 24948 | 2924 | 31 | *Sus* femur l | 560 | 16.43 | 2.9 | 43.8 | -18.1 | 7.3 | 3.2 | 1214 | 3 | 03 | room |
| 24949 | 2982 | 30 | *Sus* femur l | 590 | 23.38 | 4 | 43.7 | -18.1 | 7.7 | 3.2 | 1214 | 3 | 03 | room |
| 24950 | 2916 | 31 | *Bos* femur r | 640 | 55.9 | 8.7 | 43.3 | -13.4 | 6.8 | 3.2 | 1217 | 3 | 03 | room |
| 24951 | 2989 | 31 | *Bos* femur left | 600 | 49.7 | 8.3 | 43.8 | -17 | 6.2 | 3.2 | 21028 | 3 | 87 | yard |
| 24952 | 3018 | 30 | *Bos* femur l | 710 | 66.47 | 9.4 | 43.1 | -13.3 | 5.8 | 3.2 | 33068 | 3 | 34 | yard |
| 24953 | 2995 | 32 | *Bos* femur r | 590 | 21.59 | 3.7 | 44.7 | -17.3 | 4.5 | 3.2 | 33074 | 3 | 34 | yard |
| 24954 | 2933 | 32 | *Sus* humerus r | 600 | 31.16 | 5.2 | 43.3 | -18.5 | 5.3 | 3.2 | 2308.1 | 4 | 80 | yard |
| 24955 | 3010 | 32 | *Sus* humerus r | 620 | 49.73 | 8 | 44.2 | -18.7 | 8 | 3.2 | 2308.2 | 4 | 80 | yard |
| 24956 | 2976 | 31 | *Sus* humerus r | 650 | 48.29 | 7.4 | 44.5 | -19.7 | 7.4 | 3.2 | 6443 | 4 | 98 | yard |
| 24957 | 2920 | 31 | *Sus* humerus left | 610 | 25.59 | 4.2 | 43.7 | -16 | 7.4 | 3.2 | 7415 | 4 | 94 | street |
| 24958 | 3094 | 33 | *Sus* humerus r | 600 | 38.58 | 6.4 | 44.1 | -16.1 | 7.4 | 3.2 | 21052 | 4 | 24 | street |
| 24959 | 2911 | 31 | *Bos* femur l | 540 | 34.41 | 6.4 | 43.9 | -14.3 | 6.5 | 3.2 | 36105 | 4 | 92 | street |
| 25051 | 2974 | 29 | *Bos* humerus l | 550 | 17.54 | 3.2 | 45.1 | -13.3 | 5.9 | 3.2 | 2318 | 5 | 04 | - |
| 25052 | 2971 | 30 | *Bos* | 580 | 21.64 | 3.7 | 43.9 | -18.5 | 5.1 | 3.2 | 2407 | 5 | 02 | - |
| 25053 | 3027 | 30 | *Bos* humerus r | 620 | 12.38 | 2 | 43.7 | -15.4 | 6.8 | 3.2 | 6058 | 5 | 50 | - |
| 25054 | 2995 | 30 | *Sus* humerus r | 560 | 9.85 | 1.8 | 42.8 | -15.1 | 8 | 3.3 | 27070 | 5 | 28 | - |
| 25055 | 2969 | 30 | *Bos* tibia l | 500 | 21.1 | 4.2 | 43.4 | -14 | 5.6 | 3.2 | 33167 | 5 | 41 | - |
| 25056 | 3074 | 30 | *Sus* humerus r | 580 | 18.86 | 3.3 | 44.7 | -19.2 | 6.9 | 3.2 | 14096 | 6 | 22 | street |
| 25057 | 3015 | 30 | *Sus* humerus r | 600 | 15.13 | 2.5 | 43.4 | -20.5 | 5.9 | 3.2 | 26087 | 6 | 58 | yard |

| | | | | | | | | | | | | | | |
|---|---|---|---|---|---|---|---|---|---|---|---|---|---|---|
| 25058 | 2976 | 31 | *Sus* humerus r | 550 | 10.35 | 1.9 | 43 | -18.7 | 8.7 | 3.2 | 26087 | 6 | 58 | yard |
| 25059 | 3052 | 30 | *Sus* humerus l | 600 | 8.12 | 1.4 | 42.2 | -18.6 | 8.4 | 3.2 | 37099 | 6 | 53 | room |
| 25060 | 3032 | 29 | *Sus* humerus r | 570 | 20.02 | 3.5 | 43.9 | -18.5 | 7.1 | 3.2 | 2551 | 7 | 10 | street |
| 25061 | 3048 | 30 | *Ovis* humerus l | 480 | 16.2 | 3.4 | 42.8 | -18.2 | 6 | 3.2 | 2646 | 7 | 08 | room |
| 25062 | 3087 | 31 | *Bos* tibia l | 740 | 44.9 | 6.1 | 42.3 | -16.9 | 4.5 | 3.2 | 14047 | 7 | 28 | yard |
| 25063 | 3070 | 31 | *Ovis* tibia r | 720 | 23.3 | 3.2 | 41.8 | -17 | 5.7 | 3.2 | 24060 | 7 | 22 | street |
| 25064 | 3071 | 30 | *Bos* femur r | 740 | 24.45 | 3.3 | 43.1 | -19.9 | 5.4 | 3.2 | 15004 | 8 | 09 | room |
| 25065 | 3098 | 30 | *Sus* femur l | 580 | 15 | 2.6 | 41.4 | -19.1 | 9.2 | 3.2 | 24031 | 8 | 24 | yard |
| 25066 | 3053 | 30 | *Sus* femur l | 720 | 23.2 | 3.2 | 44.5 | -17.7 | 7.2 | 3.2 | 24040 | 8 | 24 | yard |
| 25067 | 3094 | 30 | *Sus* femur l | 530 | 14.6 | 2.8 | 42.3 | -19.3 | 7.2 | 3.2 | 25057 | 8 | 05 | room |
| 25068 | 3048 | 29 | *Sus* femur l | 590 | 6 | 1 | 40.5 | -19.1 | 7.9 | 3.2 | 47016 | 8 | 53 | room |
| 25069 | 3096 | 30 | *Sus* humerus l | 690 | 10 | 1.4 | 42.1 | -16.4 | 6 | 3.2 | 6914 | 9 | 10 | street |
| 25070 | 2920 | 29 | *Sus* humerus l | 540 | 15.87 | 2.9 | 41.8 | -18.1 | 5.8 | 3.2 | 3425 | 1 | 01 | room |
| 25071 | 2900 | 31 | *Bos* humerus r | 790 | 9.8 | 1.2 | 40.7 | -19.1 | 5.4 | 3.2 | 30030 | 1 | 82 | yard |
| 25072 | 2855 | 30 | *Bos* humerus r | 630 | 11.2 | 1.8 | 41.1 | -14.7 | 6.5 | 3.2 | 36045 | 1 | 30 | wall |
| 25073 | 2969 | 32 | *Bos* humerus l | 670 | 5.9 | 0.9 | 40.5 | -12.9 | 5.1 | 3.3 | 16032 | 2 | 63 | street |
| OxA- | Radio carbon age BP | +/- | Species/ element | Used (mg) | Yield (mg) | %Yld | %C | $\delta^{13}C$ (‰) | $\delta^{15}N$ (‰) | C:N atomic ratio | Zembil/ context number | Phase | Room/ area | Context type |

**Two samples, OxA-24948 & OxA-24949, were taken from the same bone found on the floor of Phase 3 Room 3 and the results were combined within the Bayesian model.

**Table S2: Oak timbers used for dendrochronology.**

| | Sample code | Relative Years | Used for DWM | Zembil/ Context number | Room/ area | Context type | Timber characteristics |
|---|---|---|---|---|---|---|---|
| **Phases 2 and 3** | | | | | | | |
| Phase 2 | ASR-5 | 1025-1091 | N | 1471 | 14 | in situ against W wall of Room 14 | 0.10m radius |
| | ASR-6&7 | 991-1094 | Y | 32049 | 14 | central roof support Room 14 | 0.14m radius |
| Phase 3: | ASR-15 | 1008-1084 | Y | 32058 | 13 | against west wall of Room 13 | |
| | ASR-16 | 1011-1077 | Y | 33068 | 34 | in debris to east of Room 13 | |
| **Phase 6+5** | | | | | | | |
| Phase 6 | ASR-3 | 1016-1089 | Y | 43011 | 20 | fallen beam/post close to W wall on destruction floor | 0.08m radius |
| | ASR-4 | 999-1085 | N | 43011 | 20 | post in W wall of room 20 | |
| | ASR-13 | 1011-1080 | Y | 33143 | 10 | fallen beam/post in street | traces of bark |
| Phase 5 | ASR-17 | 998-1080 | Y | 33117 | 30 | post in wall | |
| **Phase 9** | ASR-2&18 | 1000-1056 | Y | - | 09 | central post/roof support | 0.09m radius, 1/4 segment with pith and waney edge |

**Table S3: Oak timbers used for Dendrochronological wiggle-matching (DWM)**

| | Sample id | Phase from which recovered | Ring nos | Midpoint | Gap | Radio carbon age BP | +/- | δ13 | Zembil/ context number |
|---|---|---|---|---|---|---|---|---|---|
| | | | | | | | | | |
| DWM 3 | *Three timbers with matching ring patterns were used to provide the individual samples* | | | | | | | | |
| HD-23249 | ASS 6&7 | 2 | 991-1000 | 995.5 | | 2935 | 14 | -25.1 | 32049 |
| HD-23250 | ASS 6&7 | 2 | 1010-1020 | 1015 | 20.5 | 2962 | 16 | -25.9 | 32049 |
| HD-21076 | ASS 16 | 3 | 1016-1028 | 1022 | 7 | 2925 | 29 | | 33068 |
| HD-23438 | ASR 16 | 3 | 1026-1038 | 1032 | 10 | 3009 | 22 | | 33068 |
| HD-23254 | ASS 6&7 | 2 | 1040-1050 | 1045 | 13 | 2927 | 16 | -26.5 | 32049 |
| HD-23251 | ASS 6&7 | 2 | 1060-1070 | 1065 | 20 | 2929 | 16 | -26.1 | 32049 |
| HD-21077 | ASS 6 | 2 | 1079-1091 | 1085 | 20 | 2906 | 23 | | 32049 |
| | Total rings | 106 | Last ring | 1097 | 12 | | | | |
| | | | | | | | | | |
| DWM 6 | *Three timbers with matching ring patterns were used to provide the individual samples* | | | | | | | | |
| HD-23274 | ASS 13&17 | 6 | 1000-1010 | 1005.5 | | 3045 | 17 | -27 | 33143/33117 |
| HD-23273 | ASS 13&17 | 6 | 1020-1030 | 1019 | 20 | 3071 | 15 | -24.9 | 33143/33118 |
| HD-21378 | ASS 3 | 6 | 1034-1053 | 1043.5 | 24.5 | 3085 | 37 | | 43011 |
| HD-23275 | ASS 13&17 | 6 | 1040-1050 | 1045 | 1.5 | 3074 | 19 | -25.1 | 33143/33117 |
| HD-23276 | ASS 13&17 | 6 | 1050-1060 | 1055 | 10 | 3030 | 22 | -24.9 | 33143/33118 |

| | | | | | | | | | |
|---|---|---|---|---|---|---|---|---|---|
| | Total rings | 100 | Last ring | 1100 | 45 | | | | |
| | | | | | | | | | |
| DWM 9 | | | | | | | | | |
| HD-24539 | ASS 18B | 9 | 1005-1014 | 1009.5 | | 3109 | 22 | | - |
| HD-24538 | ASS 18F | 9 | 1045-1054 | 1049.5 | 40 | 3063 | 15 | | - |
| | Total rings | 56 | Last ring | 1056 | 6.5 | | | | |

**Table S4: Crop seeds from Phase 9 Granary Room 9, and pithos in Phase 6 Room 20.**

*Nb δ13 value for Panicum milicaeum, a C4 plant.*

| | Sample no | Phase | Species | Original | +/- | Final | δ13 | Zembile/context number | Room / area | Sample location |
|---|---|---|---|---|---|---|---|---|---|---|
| HD-25508 | ASS 2006-101 | 9 | Triticum monococcum | 3046 | 14 | 3027 | -24 | 15085 | 09 | On floor in N |
| HD-25509 | ASS 2006-102 | 9 | Panicum milicaeum | 3071 | 12 | 3061 | -11.7 | 15055 | 09 | Pithos pit Q |
| HD-25512 | ASS 2006-103 | 9 | Triticum monococcum | 3033 | 12 | 3022 | -24.3 | 15055 | 09 | Bin E |
| HD-25514 | ASS 2006-104 | 9 | Vicia ervilia | 3061 | 13 | 3050 | -26.1 | 15055 | 09 | Pithos O |
| HD-25522 | ASS 2006-105 | 9 | Vicia ervilia | 3057 | 14 | 3049 | -26.3 | 15055 | 09 | On floor in S |
| HD-25523 | ASS 2006-106 | 9 | Triticum monococcum | 3047 | 14 | 3043 | -24.1 | 15055 | 09 | Against S wall |
| HD-25524 | ASS 2006-107 | 9 | Triticum dicoccum | 3036 | 13 | 3023 | -23.6 | - | 09 | On floor |
| HD-22517 | ASS 2006-108 | 6 | Triticum dicoccum | 2937 | 23 | 2922 | -23 | 43011 | 20 | From base of pithos |

**Table S5: Results of the Bayesian modelling** (see Table 11, Appendix 1 for the model code).

The modelled results are given in italics, the calibrated ages prior to modelling are in the non italicised column. Convergence values are also listed. In Bayesian MCMC models these values indicate the time taken for the data to calculate or converge. They are acceptably high in the case of the Assiros model. See Figure S2 for the model itself.

| | Unmodelled (BC/AD) | | | | Modelled (BC/AD) | | | | |
|---|---|---|---|---|---|---|---|---|---|
| | 68.2% prob. from | to | 95.4% prob. from | to | 68.2% prob. from | to | 95.4% prob. from | to | Convergence value (%) |
| End 1 | -1152 | -852 | -1152 | -852 | *-1058* | *-1026* | *-1067* | *-1004* | 97 |
| OxA-25072 | -1056 | -940 | -1122 | -926 | *-1061* | *-1034* | *-1070* | *-1014* | 99 |
| OxA-25071 | -1127 | -1022 | -1213 | -999 | *-1062* | *-1034* | *-1070* | *-1015* | 99.2 |
| OxA-25070 | -1192 | -1052 | -1254 | -1016 | *-1062* | *-1035* | *-1070* | *-1016* | 99.2 |
| Use 1 | | | | | | | | | |
| Hiatus 2/1 | -1202 | -902 | -1202 | -902 | *-1066* | *-1042* | *-1072* | *-1024* | 99.3 |
| Destruction Fire 2 | | | | | | | | | |
| | | | | | *-1070* | *-1055* | *-1078* | *-1042* | 99.6 |
| OxA-25073 | -1261 | -1130 | -1310 | -1056 | *-1072* | *-1059* | *-1079* | *-1050* | 99.7 |
| OxA-24946 | -1193 | -1054 | -1256 | -1019 | *-1073* | *-1058* | *-1079* | *-1050* | 99.7 |
| OxA-24947 | -1194 | -1056 | -1258 | -1022 | *-1073* | *-1058* | *-1080* | *-1050* | 99.7 |
| BM-1426 | -1045 | -844 | -1192 | -808 | *-1073* | *-1058* | *-1079* | *-1048* | 99.7 |
| Use 2 | | | | | | | | | |
| | | | | | *-1075* | *-1065* | *-1081* | *-1056* | 99.8 |
| Cutting Estimate dwm3 | | | | | *-1078* | *-1068* | *-1083* | *-1062* | 99.9 |
| HD-21077 | -1129 | -1042 | -1207 | -1011 | *-1090* | *-1080* | *-1095* | *-1074* | 99.9 |
| HD-23251 | -1195 | -1058 | -1251 | -1051 | *-1110* | *-1100* | *-1115* | *-1094* | 99.9 |
| HD-23254 | -1193 | -1057 | -1212 | -1050 | *-1130* | *-1120* | *-1135* | *-1114* | 99.9 |
| HD-23438 | -1312 | -1213 | -1377 | -1132 | *-1143* | *-1133* | *-1148* | *-1127* | 99.9 |
| HD-21076 | -1193 | -1055 | -1256 | -1021 | *-1153* | *-1143* | *-1158* | *-1137* | 99.9 |
| HD-23250 | -1256 | -1130 | -1261 | -1126 | *-1159* | *-1150* | *-1165* | *-1144* | 99.9 |
| HD-23249 | -1208 | -1117 | -1251 | -1056 | *-1180* | *-1170* | *-1186* | *-1164* | 99.9 |
| dwm3 | -1100 | -1066 | -1114 | -1061 | *-1078* | *-1068* | *-1083* | *-1062* | 99.9 |
| Reconstr 2 | | | | | | | | | |
| Transition 3/2 | -1202 | -952 | -1202 | -952 | *-1080* | *-1070* | *-1087* | *-1065* | 99.9 |
| Destruction 3 Fire | | | | | | | | | |
| | | | | | *-1086* | *-1074* | *-1093* | *-1069* | 99.7 |
| OxA-24948* | -1193 | -1054 | -1258 | -1016 | | | | | |
| OxA-24949* | -1268 | -1131 | -1371 | -1119 | | | | | |
| *combined | -1250 | -1129 | -1265 | -1057 | *-1090* | *-1076* | *-1094* | *-1071* | 99.7 |
| OxA-24950 | -1192 | -1049 | -1255 | -1011 | *-1090* | *-1076* | *-1094* | *-1071* | 99.6 |
| OxA-24951 | -1295 | -1132 | -1371 | -1125 | *-1090* | *-1076* | *-1094* | *-1071* | 99.7 |
| OxA-24953 | -1306 | -1133 | -1375 | -1126 | *-1090* | *-1076* | *-1094* | *-1071* | 99.7 |
| OxA-24952 | -1371 | -1216 | -1389 | -1132 | *-1090* | *-1076* | *-1094* | *-1071* | 99.7 |
| Use 3 | | | | | | | | | |
| | | | | | *-1090* | *-1080* | *-1096* | *-1074* | 99.6 |
| Dendro C14 WM | -1089 | -1074 | -1094 | -1070 | *-1094* | *-1082* | *-1099* | *-1077* | 99.7 |

| | | | | | | | | | |
|---|---|---|---|---|---|---|---|---|---|
| Rebuilding 3 | | | | | | | | | |
| | -1302 | -952 | -1302 | -952 | | | | | |
| Transition 4/3 | -1302 | -952 | -1302 | -952 | *-1107* | *-1082* | *-1140* | *-1078* | 99.9 |
| | | | | | *-1161* | *-1111* | *-1190* | *-1093* | 99.3 |
| OxA-24959 | -1191 | -1042 | -1252 | -1009 | *-1174* | *-1123* | *-1201* | *-1110* | 99.4 |
| OxA-24957 | -1193 | -1051 | -1256 | -1014 | *-1173* | *-1124* | *-1202* | *-1112* | 99.4 |
| OxA-24954 | -1211 | -1056 | -1261 | -1026 | *-1171* | *-1126* | *-1202* | *-1114* | 99.5 |
| OxA-24956 | -1265 | -1130 | -1370 | -1059 | *-1172* | *-1129* | *-1202* | *-1120* | 99.4 |
| OxA-24955 | -1370 | -1212 | -1385 | -1129 | *-1174* | *-1129* | *-1205* | *-1122* | 99.5 |
| OxA-24958 | -1416 | -1316 | -1434 | -1271 | *-1176* | *-1128* | *-1206* | *-1113* | 99.4 |
| Use 4 | | | | | | | | | |
| | | | | | *-1188* | *-1139* | *-1214* | *-1129* | 99.3 |
| Construction 4 | | | | | | | | | |
| | -1352 | -1052 | -1352 | -1052 | | | | | |
| Transition 5/4 | -1352 | -1052 | -1352 | -1052 | *-1217* | *-1169* | *-1232* | *-1145* | 99.6 |
| | | | | | *-1232* | *-1197* | *-1246* | *-1166* | 99.6 |
| OxA-25055 | -1261 | -1130 | -1309 | -1057 | *-1241* | *-1207* | *-1253* | *-1171* | 99.7 |
| OxA-25054 | -1304 | -1134 | -1372 | -1128 | *-1239* | *-1209* | *-1253* | *-1171* | 99.8 |
| OxA-25053 | -1374 | -1223 | -1395 | -1133 | *-1238* | *-1211* | *-1253* | *-1189* | 99.8 |
| OxA-25052 | -1261 | -1130 | -1312 | -1057 | *-1240* | *-1208* | *-1253* | *-1171* | 99.7 |
| OxA-25051 | -1262 | -1131 | -1314 | -1091 | *-1240* | *-1208* | *-1253* | *-1171* | 99.7 |
| BM 1437 | -1257 | -1012 | -1373 | -921 | *-1240* | *-1207* | *-1253* | *-1185* | 99.7 |
| Use 5 | | | | | | | | | |
| | | | | | *-1245* | *-1216* | *-1258* | *-1194* | 99.7 |
| Construction 5 | | | | | | | | | |
| | -1402 | -1052 | -1402 | -1052 | | | | | |
| Transition 6/5 | -1402 | -1052 | -1402 | -1052 | *-1255* | *-1234* | *-1265* | *-1203* | 99.8 |
| HD-25517 | -1191 | -1054 | -1251 | -1026 | *-1259* | *-1242* | *-1270* | *-1203* | 99.9 |
| Destruction Fire | | | | | | | | | |
| | | | | | *-1268* | *-1251* | *-1283* | *-1215* | 99.8 |
| OxA-25059 | -1386 | -1294 | -1410 | -1219 | *-1274* | *-1258* | *-1293* | *-1244* | 99.9 |
| OxA-25058 | -1265 | -1130 | -1370 | -1059 | *-1272* | *-1255* | *-1292* | *-1238* | 99.9 |
| OxA-25057 | -1371 | -1214 | -1386 | -1132 | *-1273* | *-1257* | *-1292* | *-1241* | 99.9 |
| OxA-25056 | -1400 | -1313 | -1416 | -1266 | *-1274* | *-1258* | *-1293* | *-1226* | 99.9 |
| BM-1428 | -1212 | -1000 | -1310 | -907 | *-1272* | *-1255* | *-1292* | *-1238* | 99.9 |
| Use 6 | | | | | | | | | |
| start use 6 | -1402 | -1102 | -1402 | -1102 | *-1276* | *-1261* | *-1300* | *-1253* | 99.8 |
| Cutting Estimate dwm6 | | | | | *-1280* | *-1263* | *-1300* | *-1260* | 99.8 |
| HD-23276 | -1371 | -1264 | -1386 | -1215 | *-1325* | *-1308* | *-1345* | *-1305* | 99.8 |
| HD-23275 | -1395 | -1316 | -1411 | -1296 | *-1335* | *-1318* | *-1355* | *-1315* | 99.8 |
| HD-21378 | -1411 | -1315 | -1431 | -1266 | *-1336* | *-1320* | *-1357* | *-1316* | 99.8 |
| HD-23273 | -1391 | -1316 | -1408 | -1301 | *-1361* | *-1344* | *-1382* | *-1339* | 99.7 |
| HD-23274 | -1378 | -1272 | -1386 | -1265 | *-1381* | *-1364* | *-1402* | *-1359* | 99.7 |

| | | | | | | | | | |
|---|---|---|---|---|---|---|---|---|---|
| dwm6 | -1275 | -1255 | -1285 | -1238 | *-1280* | *-1263* | *-1300* | *-1260* | 99.8 |
| Reconstruction 6 | | | | | | | | | |
| | -1502 | -1152 | -1502 | -1152 | | | | | |
| End use 7 to ?earthquake | -1502 | -1152 | -1502 | -1152 | *-1293* | *-1266* | *-1312* | *-1264* | 99.4 |
| BM-1429 | -1265 | -1024 | -1386 | -933 | *-1306* | *-1273* | *-1320* | *-1267* | 99 |
| OxA-25063 | -1396 | -1311 | -1415 | -1265 | *-1309* | *-1272* | *-1322* | *-1267* | 98.9 |
| OxA-25062 | -1412 | -1316 | -1429 | -1271 | *-1310* | *-1272* | *-1323* | *-1268* | 98.8 |
| OxA-25061 | -1383 | -1271 | -1409 | -1216 | *-1307* | *-1273* | *-1321* | *-1267* | 99 |
| OxA-25060 | -1377 | -1261 | -1396 | -1210 | *-1306* | *-1273* | *-1320* | *-1267* | 99 |
| Use 7 | | | | | | | | | |
| | -1602 | -1152 | -1602 | -1152 | | | | | |
| Transition 8/7 | -1602 | -1152 | -1602 | -1152 | *-1332* | *-1300* | *-1341* | *-1282* | 99.3 |
| Destruction 8 | | | | | | | | | |
| | | | | | *-1340* | *-1315* | *-1353* | *-1302* | 99.2 |
| OxA-25068 | -1383 | -1271 | -1407 | -1219 | *-1345* | *-1321* | *-1357* | *-1311* | 99.3 |
| OxA-25067 | -1416 | -1316 | -1433 | -1294 | *-1343* | *-1322* | *-1357* | *-1312* | 99.3 |
| OxA-25066 | -1386 | -1296 | -1410 | -1220 | *-1345* | *-1321* | *-1357* | *-1311* | 99.3 |
| OxA-25065 | -1419 | -1318 | -1435 | -1296 | *-1343* | *-1322* | *-1357* | *-1312* | 99.3 |
| OxA-25064 | -1396 | -1312 | -1414 | -1266 | *-1344* | *-1322* | *-1357* | *-1312* | 99.3 |
| Use 8 | | | | | | | | | |
| | | | | | *-1352* | *-1327* | *-1362* | *-1318* | 99.2 |
| Reconstr 8 | | | | | | | | | |
| | -1702 | -1202 | -1702 | -1202 | | | | | |
| Transition 9/8 | -1702 | -1202 | -1702 | -1202 | *-1361* | *-1341* | *-1370* | *-1334* | 98.9 |
| destruction event | | | | | *0* | *13* | *0* | *28* | 99.9 |
| HD-25524 | -1368 | -1262 | -1376 | -1216 | *-1365* | *-1346* | *-1372* | *-1341* | 98.9 |
| HD-25523 | -1375 | -1271 | -1386 | -1263 | *-1365* | *-1346* | *-1372* | *-1340* | 98.9 |
| HD-25522 | -1382 | -1310 | -1393 | -1270 | *-1365* | *-1346* | *-1373* | *-1340* | 99 |
| HD-25514 | -1379 | -1301 | -1387 | -1268 | *-1365* | *-1346* | *-1372* | *-1340* | 98.9 |
| HD-25512 | -1367 | -1261 | -1375 | -1216 | *-1365* | *-1346* | *-1372* | *-1341* | 98.9 |
| HD-25509 | -1391 | -1316 | -1406 | -1305 | *-1365* | *-1346* | *-1374* | *-1339* | 98.9 |
| HD-25508 | -1369 | -1263 | -1379 | -1218 | *-1365* | *-1346* | *-1372* | *-1340* | 98.9 |
| Destruction by fire | | | | | | | | | |
| End use 9 | | | | | *-1370* | *-1351* | *-1378* | *-1343* | 98.9 |
| OxA-25069 | -1418 | -1317 | -1434 | -1295 | *-1374* | *-1354* | *-1381* | *-1345* | 99 |
| Use 9 | | | | | | | | | |
| Cutting Estimate dwm9 | | | | | *-1377* | *-1357* | *-1385* | *-1347* | 99 |
| HD-24538 | -1386 | -1313 | -1402 | -1271 | *-1383* | *-1363* | *-1391* | *-1353* | 99 |
| HD-24539 | -1422 | -1324 | -1434 | -1316 | *-1423* | *-1403* | *-1431* | *-1393* | 99 |
| | -1374 | -1336 | -1386 | -1281 | *-1377* | *-1357* | *-1385* | *-1347* | 99 |
| | -1802 | -1202 | -1802 | -1202 | | | | | |
| Start 9 | -1802 | -1202 | -1802 | -1202 | *-1381* | *-1356* | *-1395* | *-1346* | 98.9 |

**Table S6: Results of the Bayesian outlier modelling.**

The prior outlier probability was set at 0.05. The table lists the posterior outlier results, it can be seen that there are only two outliers of significance (OxA-24952 and -24958). 'Model' denotes the different outlier models used (see Bronk Ramsey 2009).

| Element | Prior | Posterior | Model | Type | | Element | Prior | Posterior | Model | Type |
|---|---|---|---|---|---|---|---|---|---|---|
| HD-24539 | 5 | 3 | RScaled | R | | OxA-25051 | 5 | 2 | General | t |
| HD-24538 | 5 | 3 | RScaled | R | | OxA-25052 | 5 | 2 | General | t |
| OxA-25069 | 5 | 3 | General | T | | OxA-25053 | 5 | 4 | General | t |
| HD-25508 | 5 | 2 | General | T | | OxA-25054 | 5 | 2 | General | t |
| HD-25509 | 5 | 2 | General | T | | OxA-25055 | 5 | 2 | General | t |
| HD-25512 | 5 | 3 | General | T | | OxA-24958 | 5 | 83 | General | t |
| HD-25514 | 5 | 1 | General | T | | OxA-24955 | 5 | 5 | General | t |
| HD-25522 | 5 | 1 | General | T | | OxA-24956 | 5 | 3 | General | t |
| HD-25523 | 5 | 1 | General | T | | OxA-24954 | 5 | 2 | General | t |
| HD-25524 | 5 | 2 | General | T | | OxA-24957 | 5 | 3 | General | t |
| OxA-25064 | 5 | 2 | General | T | | OxA-24959 | 5 | 3 | General | t |
| OxA-25065 | 5 | 2 | General | T | | OxA-24952 | 5 | 68 | General | t |
| OxA-25066 | 5 | 2 | General | t | | OxA-24953 | 5 | 23 | General | t |
| OxA-25067 | 5 | 2 | General | t | | OxA-24951 | 5 | 20 | General | t |
| OxA-25068 | 5 | 2 | General | t | | OxA-24950 | 5 | 2 | General | t |
| OxA-25060 | 5 | 2 | General | t | | (24948 and 24949 | 5 | 7 | General | t |
| OxA-25061 | 5 | 2 | General | t | | OxA-24949 | 5 | 15 | SSimple | s |
| OxA-25062 | 5 | 5 | General | t | | OxA-24948 | 5 | 2 | SSimple | s |
| OxA-25063 | 5 | 3 | General | t | | HD-23249 | 5 | 3 | RScaled | r |
| BM-1429 | 5 | 4 | General | t | | HD-23250 | 5 | 3 | RScaled | r |
| HD-23274 | 5 | 3 | RScaled | r | | HD-21076 | 5 | 3 | RScaled | r |
| HD-23273 | 5 | 3 | RScaled | r | | HD-23438 | 5 | 5 | RScaled | r |
| HD-21378 | 5 | 3 | RScaled | r | | HD-23254 | 5 | 3 | RScaled | r |
| HD-23275 | 5 | 3 | RScaled | r | | HD-23251 | 5 | 3 | RScaled | r |
| HD-23276 | 5 | 3 | RScaled | r | | HD-21077 | 5 | 3 | RScaled | r |
| BM-1428 | 5 | 5 | General | t | | BM-1426 | 5 | 5 | General | t |
| OxA-25056 | 5 | 10 | General | t | | OxA-24947 | 5 | 2 | General | t |
| OxA-25057 | 5 | 2 | General | t | | OxA-24946 | 5 | 2 | General | t |
| OxA-25058 | 5 | 3 | General | t | | OxA-25073 | 5 | 7 | General | t |
| OxA-25059 | 5 | 4 | General | t | | OxA-25070 | 5 | 3 | General | t |
| HD-25517 | 5 | 9 | General | t | | OxA-25071 | 5 | 2 | General | t |
| BM 1437 | 5 | 3 | General | t | | OxA-25072 | 5 | 2 | General | t |

**Section B) Assiros Toumba: the sequence of building levels and the DWM samples**[3,4]

The dates given in this account of each phase are those based on the *conventional* historical chronology, as suggested at the time of excavation. The new absolute dates resulting from the Bayesian analysis of the $^{14}C$ determinations are tabulated and the offsets discussed in the main paper (Table 1) with additional information about contexts and significance below.

*PHASES 9 & 8: Assiros as a 'central place'*

The deepest levels excavated over a substantial area are dominated by granaries, obvious in the burnt destruction level of Phase 9 but clearly repeated in the rebuild which followed that fire. The predominance of storage over living space suggests that Assiros had, at this period, a distinctive role in providing storage facilities for a much larger community than could have been accommodated on the summit of the Toumba, perhaps as an administrative centre.

PHASE 9 LH (Late Helladic ie Mycenaean) IIIA 2/B, *conventionally* 1350-1300 BC (Fig. S3)

The heavy burnt destruction debris of Phase 9, with its granaries and abundant charred seeds, was the excavation limit for work at Assiros and no significant tests were made below it in the centre of the mound. Architecturally the most important discovery was of the existence of three parallel streets running E–W across the mound about 9m apart, which remained in use with only minor alterations until the end of the BA, and were retained as the street alignments into the IA. At least six of the rooms excavated at this level were granaries. The crops had been stored in large wicker baskets plastered over with clay, in smaller freestanding 'bins' of chaff mixed with clay and in the large pointed-based pithoi set into the floor whose sherds were familiar in every level excavated. Room 9, for example, must have held around 18 tons of stored crops. The contents of this room alone, is enough to feed about 50 people for a year. When the other storerooms discovered are taken into account, the quantity of foodstuff stored is clearly far too great for the number of people who could have been accommodated on the summit of the Toumba.[5] The small quantities of Mycenaean pottery from this destruction level suggest a date, for Phase 9, around the transition from LH IIIA 2 to LH IIIB, i.e. *conventionally* in the latter part of the 14th or very beginning of the 13th century.[6] Most of the pottery found at this level was the typical Central Macedonian hand-made burnished ware, with four handled jars, cut-away-neck jugs and wishbone-handled bowls as the most frequent shapes.

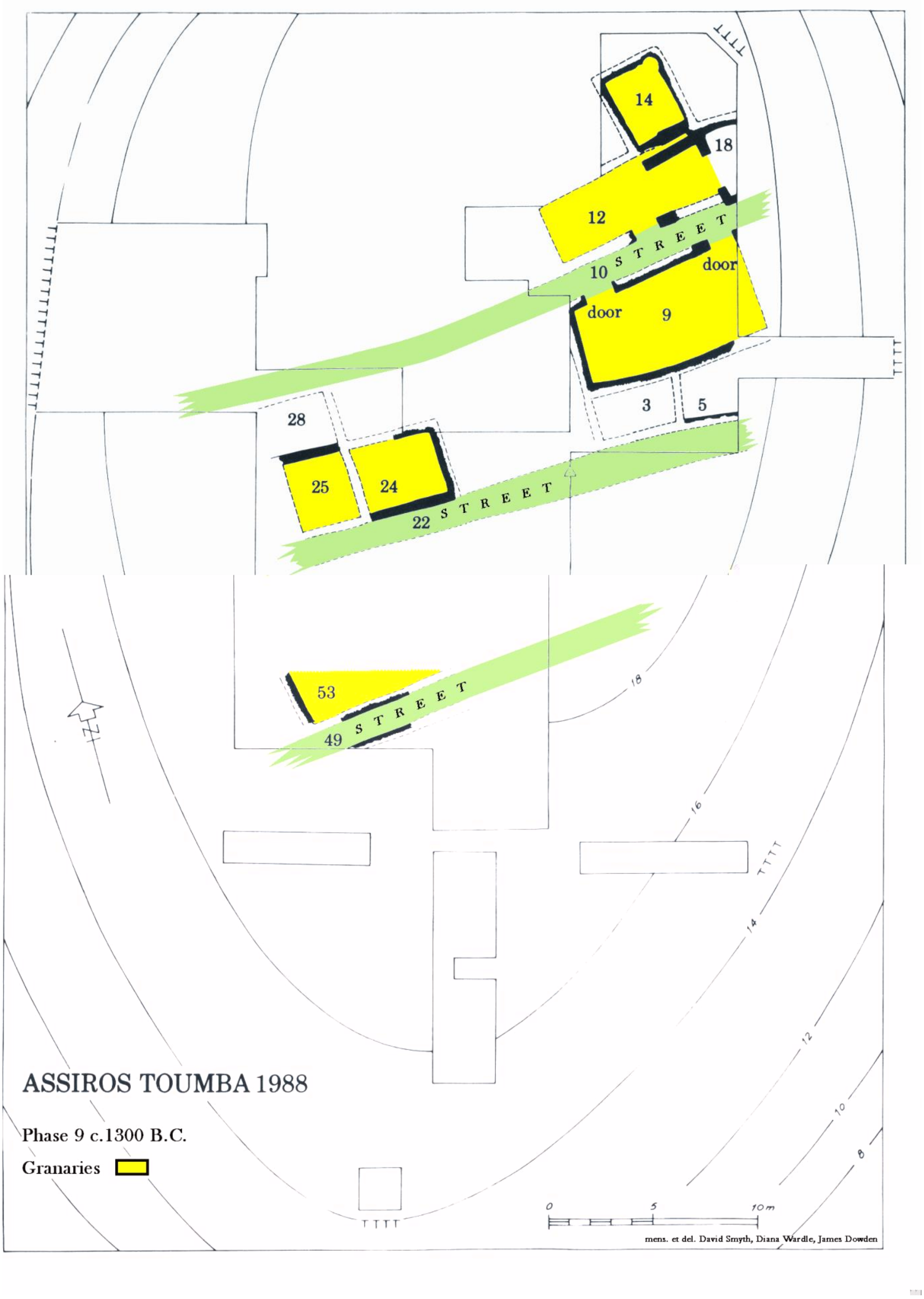


**Figure S3: Assiros Phase 9 with granaries**

PHASE 8 LH IIIB, *conventionally* 1300–1200 BC

The rebuilding of Phase 8 followed hard on salvage operations. The Phase 9 burnt walls were cut back to provide a firm footing for the new brickwork, which followed the old plan almost exactly. Several of the crop storerooms were rebuilt and reused for storage, while other rooms were used for storage for the first

time. Pits for supporting pithoi were cut through the new floors and into the burnt debris below, and the faint circles of clay bins and baskets could be traced on the floor. Elsewhere the occupation debris in this phase included enormous numbers of grape pips, preserved as silica skeletons, which perhaps indicate vinification on a large scale.[7] This phase lasted for some years since a considerable deposit of occupation debris built up in the streets and yards, reaching as much as 0.75m in places. In several places there were signs of alterations and rebuilding. The imported Mycenaean pottery can all be placed within the LH IIIB period. The area excavated at this depth is only a little larger than that of the underlying level, but the impression of large rooms and bulk storage remains the same. It seems likely that Assiros Toumba played the same role in this phase, as in the previous one, as an administrative centre. There is no clear evidence as to what caused the end of this phase — unlike Phase 9 fire is certainly not to blame — and it is possible that the whole complex was so damaged by an earthquake that widespread rebuilding was necessary.

*PHASES 7, 6 & 5: Assiros as a village community.*

With this rebuilding, the character of the complex changed. Centralised storage is replaced by scattered storage on a household scale and large rooms were subdivided to create smaller ones. This seems to indicate some major change in social structure. Three building levels, which followed this change, were excavated over an area twice as large as those underneath and a very clear idea of the settlement plan and use of the space is possible for the first two of these (Phases 7 and 6). These give a different impression, that of a village community, rather than one with a central function. The second (Phase 6) was of shorter duration (cf. Figure S1) and destroyed by fire. The third of these phases (Phase 5, the last in the Bronze Age) was too poorly preserved to determine whether it had the same character but it too was destroyed by fire.

PHASE 7 LH IIIC, *conventionally* 1200–1150 BC

The area of Phase 7 which has been excavated is considerable, (*c.* 500m$^2$) and even where it has not been possible to reach the floors, it is clear that the alignments of Phase 7 walls were retained almost exactly in Phase 6. The main features of the settlement plan had already been established, since, as usual, the old walls of Phase 8 formed the foundations for the reconstruction of Phase 7. The defensive walls on top of the terrace banks were renewed and have been located on both sides of the mound but no new terrace bank was constructed. The three streets were maintained but all the former large areas were subdivided, in some cases into much smaller rooms. It was usually possible to distinguish interiors with their clean floors from yard areas where occupation debris, including pithos sherds, animal bone and charcoal, was allowed to accumulate. The most striking change from the preceding period is the dispersal of storage so that there were now smaller quantities in almost every room. Single pithoi were found in many rooms, while clay-covered wicker baskets could be detected in several. The picture suggested by this pattern of storage is that the

settlement now comprised a number of households, each with its own roofed area and open yards, and each with its own individual provision for storage of the foodstuffs needed for immediate use. The structures probably stood for several decades to judge by the depth of the deposits in the streets and yards. The phase ended, as had the previous one, without sign of fire, but with a deeper fill of clean mud-brick debris from the fallen walls over the interior floors, which is an even stronger sign of earthquake damage. Mycenaean pottery was found in considerable amounts, but now the majority was of local Macedonian manufacture but is clearly LH IIIC in character.[8] The introduction and use of this pottery style on the basis of *conventional* chronology would normally be dated between 1200 and 1150 BC.[9]

PHASE 6 LH IIIC *conventionally* 1150–1100 BC (Fig. S4)

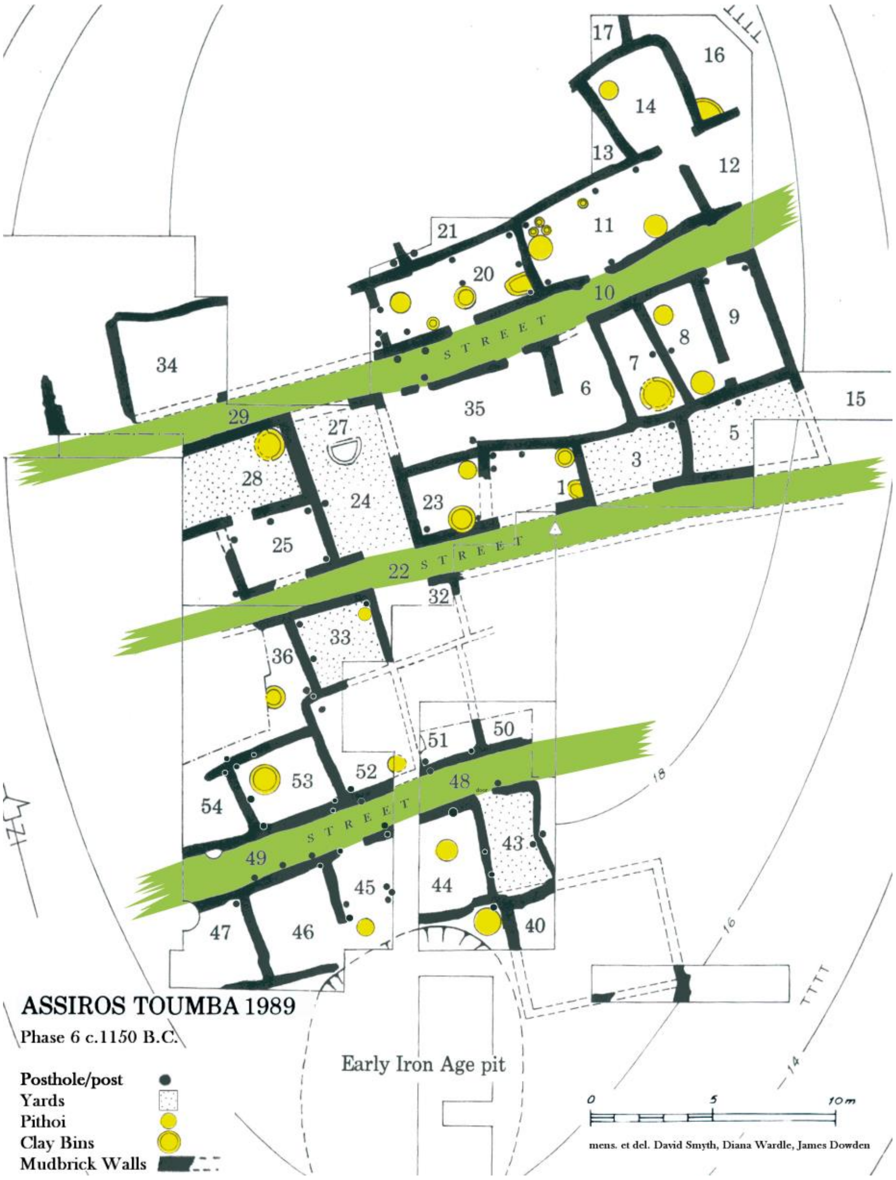


**Figure S4: Assiros Phase 6 plan**

Rebuilding took place immediately after the suggested Phase 7 earthquake but very few of the rooms changed their size or function. This settlement was in turn destroyed by fire and the floors and internal features were thus often well preserved. The evidence for dispersed storage was even clearer in this phase than in the preceding one. Over 600m$^2$ of this settlement has been uncovered and 17 'rooms' have been excavated fully, together with large parts of another 14, as well as the three streets which continued in use in this phase. The duration of this period is harder to assess since the interior floors were kept clean with no build-up of detritus and therefore remained at the same level. The majority of the Mycenaean pottery of this phase is locally made. There is little obvious change from that found in the Phase 7 levels either in the range of shapes or the simple linear and wavy decoration. It does not seem appropriate at Assiros to attempt to define the LH IIIC pottery from Assiros in the terms established for the finer subdivisions possible in southern Greece.[10] None of the diagnostic LH IIIC Late features have been identified, but it seems likely that the destruction fire took place in the later part of the LH IIIC period.

PHASE 5 LH IIIC *conventionally* 1100–1050 BC

In the rebuild of this phase many of the walls of the preceding one were ignored and substantial new stone foundations were set into the destruction debris, in other places Phase 6 walls were reused. The realignments meant that the new S and central streets, although in the same place as the old, had been shifted by 5° clockwise. The line of the north street was not identified, but was certainly *not* above the north street of Phase 6. After the eventual destruction by fire of the buildings of this phase, the walls had fallen or been overturned and in many places lay almost intact with the mud-brick on edge in clear rows. In the following IA phases these fallen walls formed, in many areas, the floor surfaces. This phase was perhaps not of very long duration, despite the effort put into rebuilding the settlement, since little occupation debris was found. Distinctive Macedonian Iron Age features, such as channelled ware or twisted handles, were not found in this phase, and there seems little doubt that it is still of Bronze Age date, although the small sherds of Mycenaean pottery found are probably largely residual.

*PHASES 4, 3, 2 & 1.5: The Iron Age Village*

Excavation of more than 700m$^2$, the greater part of the area of the Toumba summit, revealed no less than four closely superimposed Iron Age building levels (Phases 4-1.5). One level, (Phase 2), demonstrated very clearly the organised character of an IA village at the end of the second and beginning of the first millennium BC. As excavation of the earliest Iron Age levels proceeded, it became clearer that there was no hiatus between the Bronze Age and Iron Age levels and that continuity of occupation and culture were both readily demonstrable. The character and significance of the changes, which did take place at this period at Assiros, have already been outlined in a previous study[11] and are part of the much larger questions of culture

process and cultural change which affect SE Europe late in the second millennium BC. These have been to some extent obscured by the largely arbitrary division between Bronze and Iron Ages especially in a region such as Macedonia where iron working remains exceptional for perhaps another two centuries.

PHASE 4 *conventionally 1050–1000 BC but see discussion in main text for reassessment.*

Phase 4 was characterised by rather haphazard rebuilding with substantial stone foundations immediately over the debris of Phase 5 without any intervening level of debris. Continuity is strongly suggested by the reuse of the S street, with an unbroken sequence of successive scatters of sherds and other debris with each remake of the street surface. The depth of the deposits associated with this phase indicate the passage of quite a lengthy period of time but it is impossible to tell from their depth and character whether this was as much as fifty years or even longer. The first of the new Macedonian IA types of pottery appeared regularly in this level including hand-made ware with angular features such as twisted handles and rare examples of channelled ware.[12] The distinctive Macedonian wheel-turned grey ware with well-defined grooving also first appears in these levels. At Toumba Thessalonikis, this wheel-turned grey ware is found, together with the first *Macedonian* Iron Age features and Late Helladic IIIC pottery in levels 3 and 2B.[13] This may indicate, contrary to our previous evaluations, that Assiros Phase 4 is contemporary with the final stages of the *southern Greek* Late Bronze Age rather than the Early Iron Age.

PHASE 3, PG *conventionally* 1000-950 BC

This phase was best preserved in the N central part of the site where yards and two complete rectangular rooms were found measuring c. 6 x 4m (Rooms 13 and 14). Here, the traces of a destruction fire were very clear and some of the internal features were well preserved. In the West a length of rough stone wall curved round the scarp of the Toumba. This wall was perhaps the only defensive wall built in the IA. A small number of pottery vessels on the room floors, including a fine hand-made amphora with twisted handles show that the character of the local pottery is very similar to that of the next phase, (Phase 2). Most of the sherds of the very fine wheel-made amphora decorated with concentric circles in the Attic Proto-Geometric style were found on the Phase 3 floors of Rooms 13 and 14 (Main text Figure 6 and find spots diagram Figure S8). No great depth of occupation debris could be associated with this phase of construction. After the destruction, the walls of these rooms were rebuilt (Phase 2) and several new rooms added to the complex.

PHASE 2, (PG) *conventionally* 950-900 BC (Fig. S5)

Reconstruction followed immediately upon the fire which destroyed the Phase 3 buildings, but on a larger scale and with a more orderly plan. The stone perimeter wall in the NW still served to protect the settlement and the street alignments were maintained. This phase also ended with a severe destruction fire, which helped preserve many internal structures. It was also remarkable for the large number of pots found smashed on the destruction floors and for the large size of many of the pithoi and fine ware vessels. Around 120 vessels were in use at the time of the destruction and the majority of these were found complete, if broken. They provide an excellent illustration of the large range of pottery of the period. Loom weights, both purpose-made, and those formed by trimming the broken handles of large jars, were especially common in this phase. One large group from Room 10 had clearly fallen from a standing loom during the destruction fire. The duration of the phase is uncertain. There are no deep deposits of occupation debris from the streets or open areas that can be associated with this phase which would indicate a long period of use before the fire which destroyed this settlement also.

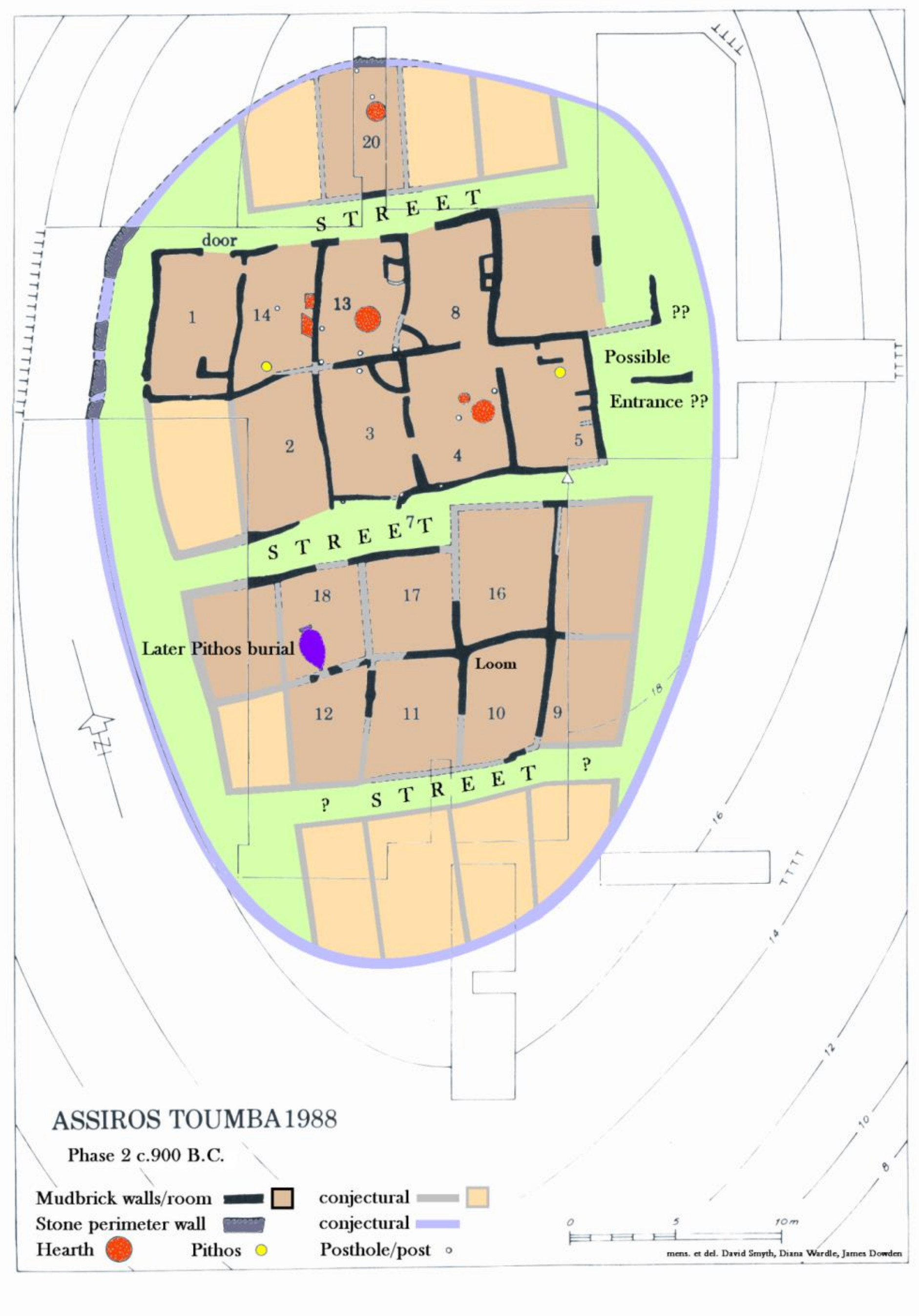


**Figure S5: Plan of village in Phase 2: as found and conjectural**

PHASE 1.5 *conventionally* 900 BC

Signs of reconstruction were found above the Phase 2 debris in several places, including lengths of un-burnt mud-brick wall, alignments of burnt mud-bricks reused in makeshift structures, and a horseshoe hearth built over burnt debris. No levels of occupation debris could be associated with these buildings and these scanty remains represent the last habitation in an unbroken sequence stretching back approximately a thousand years. There is nothing to show why the site should have been abandoned.

*Abandonment and reoccupation: Phase 1*

After the abandonment of the Phase 1.5 settlement, the Toumba remained unoccupied for a short period. During this time a single pithos burial was set into the ruins on the summit of the mound. After another interval, a pair of apsidal buildings were constructed which demonstrate another type of activity at the Toumba.

PHASE 1 (insufficient evidence to date closely on conventional grounds).[14]

The Toumba was briefly reoccupied without any regard for the former plan of the village settlement, whose ruins were by now too dilapidated to provide more than a quarry for building stone. The remains of this final phase lay immediately under the surface of the Toumba with only a slight covering of humus and hard packed clay building debris. Only in the central part of the site was the preservation good enough to make sense of the buildings which once existed. A large apsidal building lay across the centre of the mound with its apse to the E.  It was later replaced on a slightly different alignment. At the same time as this rebuilding took place, a second, larger, apsidal building was added to the S of it, but so close that only a narrow path and drainage channel was left between the two. Both buildings were destroyed in some catastrophe when they were still in use. Several pithoi were found in association with other walls in the NE of the summit, while the NW appeared to have been an open yard area. Pottery of this phase is different in quality from its predecessors though the repertoire of shapes does not alter much. The pottery is now thinner in section, and harder fired with a matt surface rather than the fine burnish of earlier phases. The character of this phase of occupation on the mound is quite different from any that preceded it. The large buildings belong to a type which is now well known in the IA from Nichoria in Messenia to Thasos and Lemnos, a type which has in the past been variously interpreted as a chief's residence or an early temple. It is, however, clear that it is an architectural form typical of large buildings of the period and that its function varies from site to site.[15] It is possible that at Assiros these buildings represent a chief's residence. The fine pottery is predominantly of shapes which could belong to drinking sets - large mixing bowls, small jars, jugs and small bowls. The largest of these is a wishbone-handled bowl with horse-head protomes for the handle ends which has a capacity not far short of 40 litres. This, one of the largest and most imposing of its type, was perhaps to be

used in a more mundane version of the heroic feasts of the Homeric epic. Alternatively, these sets of drinking vessels could indicate the existence, then as now, at a key point on the main route to the north and north east, of a ‘Hani’ (Inn) which may have served to provide shelter and refreshment to travellers or local farmers.

*Archaeological and historical significance*

The results of the excavation at Assiros are of significance in a wide range of aspects of prehistoric society. The large area excavated and the variety of different structures exposed have enabled the changing social organisation at Assiros to be explored, from the centralised storage of Phases 9 & 8, to the ‘village’ style organisation of Phases 7-5, which was repeated in a slightly different form early in the Iron Age. The abundant and often restorable local pottery allows Assiros to be placed accurately in the context of other excavated sites in the wider region, such as Kastanas and Toumba Thessalonikis. The imported or locally imitated Mycenaean and Proto-Geometric pottery enables relative chronologies to be established with southern Greece for most of the phases of construction – and thus the transfer of the absolute dates from Assiros to other regions. In view of the continuing discussions of the nature of the transition from Bronze to Iron Age in Greece, the demonstration of continuity of both occupation and cultural characteristics at Assiros tends to refute the theories of large scale population movements into Greece at this period. The crop seeds from the Phase 9 granaries have enabled a range of specialised studies - of agricultural practices on the basis of the accompanying weed seeds,[16] of the recovered DNA from the wheat seeds demonstrating its potential for making risen bread,[17] or the stable isotopes which indicate that the crops from the granaries were the produce of a single year.[18] Unexpectedly, and perhaps of most significance, the range and quantity of datable material from secure deposits underpins the first long sequence of absolute dates from the Aegean region, as discussed here, which provides a firm foundation for further studies of Aegean LBA chronology.

*The charred samples and their contexts* (Tables S2-S4)

The fierce destruction of Phase 9 preserved large quantities of charred material. These included a substantial post from the centre of the largest of the granaries (Room 9) which preserved enough rings for DWM (dendro wiggle matching) determinations (Figure S6).[19] This roof support clearly belonged to the structure of the building, and its cutting date is likely to provide the date for the construction of this room. The timber was used round and had not been trimmed beyond the bark layer so that it is unlikely that there was any significant ring loss between cutting and recovery some 3300 years later. The fact that the animal bone determinations from this phase and the immediately succeeding Phase 8, are not separated by a long interval indicates first, that this timber was not reused from an earlier context and, second, that the phase was not of any long duration. Equally it (and the other oak timbers from the site) cannot have been cut and seasoned for

several years before use. Anyone who has worked with oak for construction will know how unlikely this is in any case: green (freshly cut) oak can be cut and shaped quite readily which outweighs the probability of warping as it dries out and seasons. Once seasoned, it requires much sharper tools and greater effort to achieve the desired dimensions.

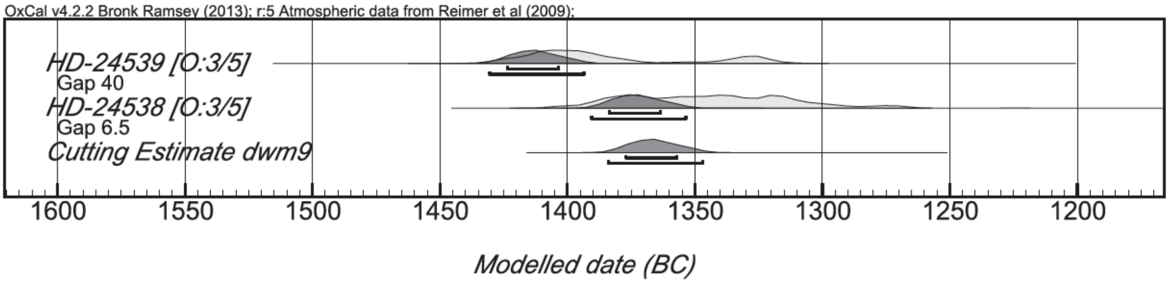


**Figure S6: Dendro wiggle match diagram for Phase 9**.

As already mentioned, large quantities of crop seeds were found in the granaries and the $^{14}$C determinations from these (Table S4) are likely to be accurate and unaffected by any inbuilt age. *A priori*, crop seeds are unlikely to have been retained in quantity for any length of time and, moreover, those from this level at Assiros probably represent only a single year's crop.[20] The quantities enabled the selection of seven discrete samples from separate storage containers representing four varieties of crop – two kinds of wheat (three samples of Triticum monococcum and one of Triticum dicoccum), bitter vetch (two samples of Vicia ervilia), and millet (one sample of Panicum milicaeum). Including dated samples such as these within our Bayesian model is crucial to the generation of robust results.

The Phase 6 destruction level provided three large pieces of burnt oak timbers from the central area of the site and a fourth was recovered in the same vicinity from the destruction fire in the immediately following Phase 5. Dendrochronological cross-matching showed that all had been growing at the same time[21] but it remains uncertain whether the timber from Phase 5 was reused, or whether the construction of Phase 5 followed so closely on that of Phase 6 that the different cutting dates cannot be distinguished. The long ring sequence identified in Cornell (106 years) offers the opportunity to cross match it with other Aegean sequences when these become available as an independent corroboration of the absolute date determined.

In earlier reports we suggested that it was possible that these timbers had been reused or survived in position from the first construction of the series of buildings in Phase 7. While we were not entirely convinced this was the correct interpretation,[22] it did serve to accommodate the DWM date better to the anticipated historical chronology for the start of the LH IIIC period. The animal bone dates, however, indicate that this premise was erroneous and that the DWM (Main text, Figure 3) gives a real date for the construction of the Phase 6 buildings.

Four further substantial pieces of charred oak were recovered from the destruction levels of Phases 3 and 2. In Phase 3 one was part of the wall framing and the other had fallen, perhaps from the roof. In Phase 2 one was part of the wall framing between Rooms 14 and 1 and the other was the central roof support in Room 14. Dendrochronological cross-matching showed that all four timbers had been growing at the same time and that those from the Phase 3 contexts had been cut ~10 years before those from the Phase 2 contexts. As with the timbers from the earlier phases, there was no indication of ring loss through trimming or shaping and the central roof support still preserved its original circularity. The obvious interpretation of this discovery is that the timbers were cut and used ten years apart – ie the interval between the construction of Phase 3 and that of Phase 2, immediately following. Even if some of the timbers of Phase 3 had survived in situ and un-burnt, the close match between the $^{14}C$ dates for the animal bones from *both* levels and to the DWM determinations (Main text, Figure 4) indicates that the cutting date for Phase 3 cannot be earlier than the date of the associated Phase 3 deposits *or those of Phase 2* by more than a handful of years and certainly not the 70 years required to align the absolute dates with the conventional historical dates.

**Section C): The current dating of end of the LBA in Greece and the new date for the start of the Proto-Geometric period and the Greek Iron Age.**

Although the historical chronology for the start of the PG period relies on a series of assumptions and constructions, scholars have been very reluctant to accept an upward revision of these dates since they were first published in 2005. It will therefore be useful to examine the strength of the evidence which supports the conventional date and to rebut the criticisms made (apart from the argument for *reused* timbers already addressed) by a number of commentators. The conventional chronology for the end of the LH IIIC period at 1050/1025 BC depends on one allegedly fixed point c 150 years earlier, assumptions about the duration of pottery phases and the presence of PG pottery in contexts in Israel whose absolute date is a matter of considerable discussion and dispute The rationale of the current conventional date for the end of LH IIIC/Sub-Mycenaean and start of PG was set out some 60 years ago by Vincent Desborough in a carefully argued series of assumptions.[23] The starting point is the incursions of the Sea Peoples in the Levant and the campaigns of Ramesses III c 1180 BC in response. Destruction levels observed at several cities including Ras Shamra (Ugarit) and Tel Kazel[24] have been associated with the invasion and the military actions. It is notoriously difficult to relate historical events and archaeological strata, though that does not usually deter the eager chronologist. These destruction levels contain derivative pottery of early LH IIIC style[25] and it is therefore assumed that LH IIIC pottery came into fashion in mainland Greece a little before this, say around 1200 BC. (We leave aside for the moment the evidence of the absolute dates from Assiros set out here that this pottery style should already be in use in the first half of the 13$^{th}$ Century BC.) Desborough identified six stages of LH IIIC pottery development with the addition of Sub-Mycenaean as a final stage – making 7 in all and suggested an end date of 1025 BC to accommodate this sequence. In his *The last Mycenaeans and their*

*successors*,[26] he preferred a date of 1050 based on additional evidence from Cyprus - which was itself dependent on dates in the Levant. Warren and Hankey took this argument a stage further and proposed that the seven distinct styles of pottery represented seven generations of potters between the start of LH IIIC and the end of Sub-Mycenaean. Allowing 25 years per generation, they calculated a Proto-Geometric start date of 1025 BC.[27] Similar evidence has been used by Lemos in her recent study of the Proto-Geometric period.[28] Some support for these dates was provided by examples of Proto-Geometric pottery in contexts in the Levant associated with the campaigns of King David as reported in the Old Testament, which are considered by some to be of early 10th C. date. Coldstream, reviewing Greek imports in the Levant, presented two schemes for understanding these centuries from the perspective of an Aegeanist working with Greek ceramics. He preferred a "low" chronological scheme, but the primary reason he offered was that the "high" chronology would present "an uncomfortable congestion" for the long development of LH IIIC and Sub-Mycenaean before the appearance of Proto-Geometric.[29] The absolute dates upon which the chronological schemes presented by Coldstream are based, come from finds of Greek imports at sites in the Levant, but these dates have been the subject of much discussion recently and this not the place for a full review of the arguments and counter arguments which affect the whole of Levantine chronology.[30]

The significance of this early date for the amphora from Assiros has been questioned by Sherratt[31] on the grounds that an Attic origin for the style as whole is still uncertain. She notes that an alternative model has been proposed by Jacob-Felsch,[32] Maier *et al.*[33] etc in which examples from Central and Northern Greece are the earliest and the style then spread from there to Athens and beyond. This, however, has won little support among the experts in Proto-Geometric pottery and on present evidence seems improbable.

Recent studies of the wheel-made and linear decorated pottery from Kastanas[34] and from Toumba Thessalonikis[35] have shown that the Macedonian PG style is developed in part from the preceding LH IIIC styles and in part from the PG of southern Greece including, presumably, the use of the characteristic compass-drawn circles and semicircles. The position is complicated at Kastanas since the key relevant stratum (Schicht 12 – see below) is reported to contain mid and late LH IIIC pottery alongside early Proto-Geometric. This phenomenon is not reported in the sequence at Toumba Thessalonikis where pottery of both classes is as abundant as at Kastanas and there is no gap in the stratigraphy to accommodate such an overlap.[36] Whilst an overlap with the latest Mycenaean/Sub-Mycenaean at Kastanas could be seen as support for Sherratt's position, it seems extremely improbable that the style could have originated in northern Greece as early as the middle (in ceramic terms) of the LH IIIC period – conventionally around 1125 BC.[37] The result would be an even earlier origin for the PG style if this were to be the correct interpretation of the Kastanas finds.

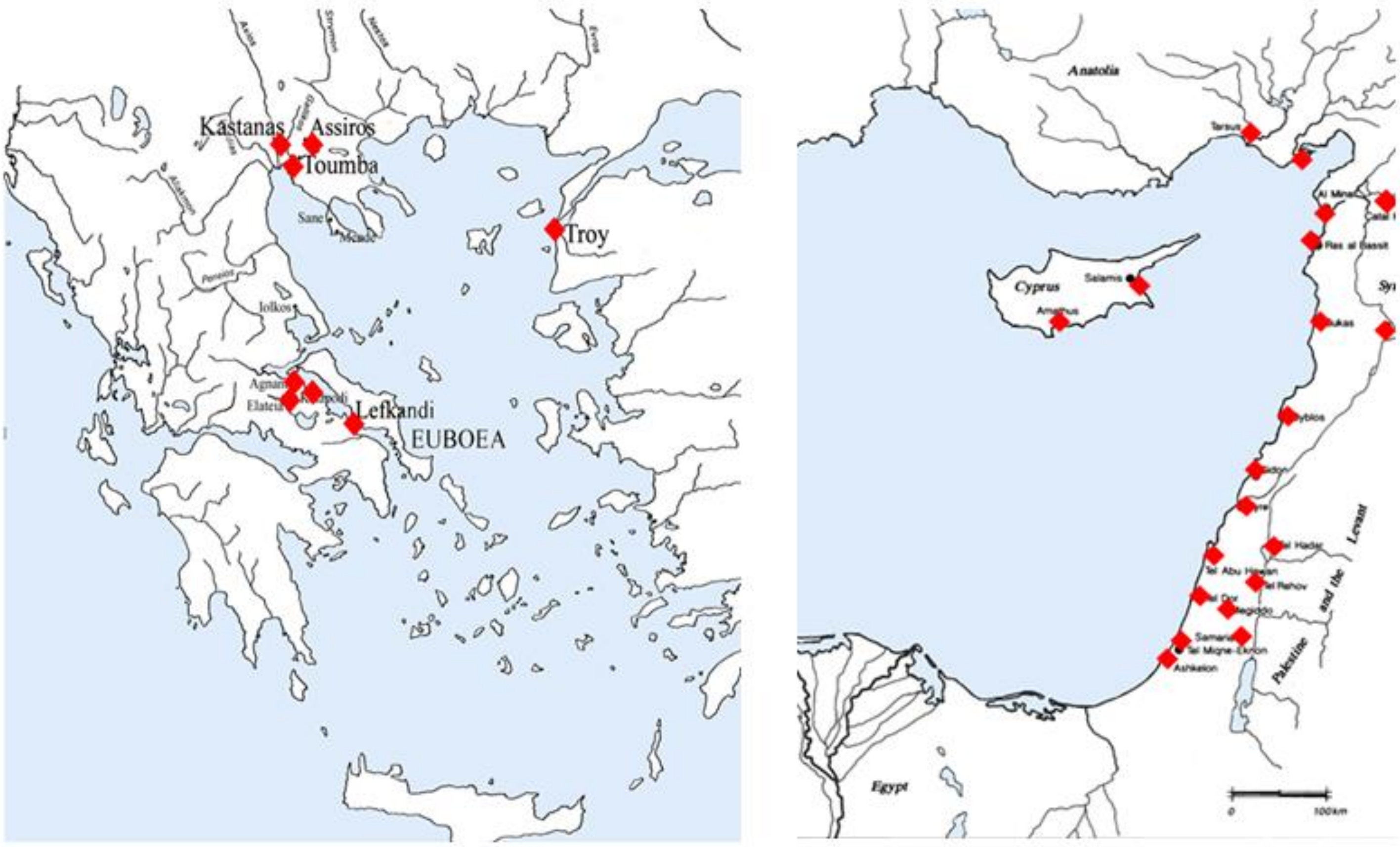


**Figure S7 *left*: Distribution of Group 1 PG amphorae *after* Catling 1998, 156: *right*. Distribution of Proto-Geometric pottery in the Levant *after* Lemos 2002, 229, Map 8**

The significance of the Assiros amphora has also been questioned on the basis that there may have been stratigraphic disturbance which resulted in the pieces of the vessel moving downwards from their original location.[38] This can be rebutted on several grounds. The first is the distribution of the find spots of the numerous and often quite large fragments (Figure S8). The greater part was found on the floor of the Phase 3 Rooms 13 and 14. One fragment was built into the E. wall of the Phase 2 Room 4. More fragments were scattered widely in Rooms 2, 3, 13, 17 of Phase 2.

The logical explanation for this distribution is that the vessel was broken during the Phase 3 destruction and partially burnt fragments ended up on the floors of two adjacent rooms. With the rebuild of the area in Phase 2 after this destruction, the underlying levels were disturbed through, for example, the pit-digging associated with new post settings, and the sherds were scattered over a wide area.

Second, it is highly unlikely that the reverse process could have happened after the Phase 2 destruction fire or that a sherd of the vessel could have become incorporated in a Phase 2 wall *after* it was built. Third, even if such a reverse taphonomic process is conceivable, the date for the use of Phase 2 provided by the animal

bone determinations for the phase is only a few years later than that for Phase 3 and the date of manufacture of the amphora remains unexpectedly early in relation to the conventional chronology.

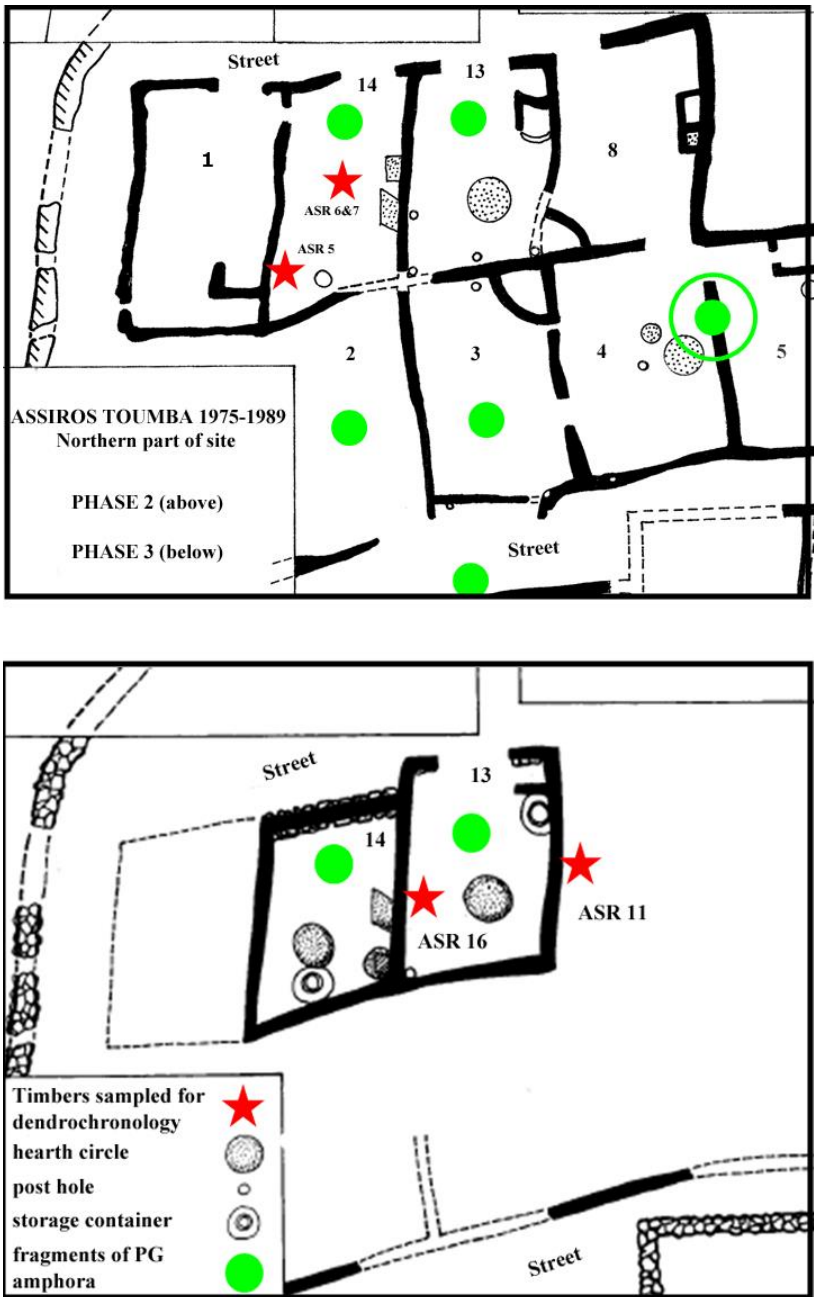


**Figure S8: Find spots diagram for pieces of the PG amphora (green) and the 4 timbers used for dendrochronology and the DWM (red).**

**Section D): Kastanas – stratigraphy and dating evidence**

Excavation and methodology

In view of the fact that $^{14}C$ dates from the settlement mound at Kastanas,[39] beside the river Axios some 35 km west of Assiros, have been used to support a different chronology which is claimed to match the conventional 'historical' chronology, we have reviewed the published data from this site to assess the

validity of these arguments. The $^{14}C$ dates from Kastanas have been modelled (Figure S11) and compared with those from Assiros, as far as is possible given the local characteristics of the pottery at each site which (*pace* Jung in his publication of the Mycenaean pottery)[40] do not allow fine distinctions between phases. There are, moreover, methodological issues arising from the excavation, recording and analytical procedures used, which belie Jung's confidence in them[41] and the over-hasty acceptance of his conclusions by other scholars.[42]

Kastanas was occupied from the Early Bronze Age until the Archaic period, and for a large part of its history has a sequence of phases which parallel those of Assiros. In the Bronze Age before the extensive alluviation in the Thermaic gulf, it appears to have been an island site in the estuary of the Axios open to influences from the southern Aegean. Excavation was directed by Bernhard Hänsel for the Deutsche Archäologisches Institut Athen and Aikaterina Rhomiopoulou for the Greek Archaeological Service between 1975 and 1979.

Excavation at Kastanas followed the then standard German practice of removing horizontal spits of earth and recording all finds by spit and by metre-square excavation units. Earth removal thus followed arbitrary rather than natural levels (unlike the practice at Assiros). Plans were made at the base of each spit to record the location of walls, pits and changes in soil colour or texture. Excavation units were then assigned to Schichten/strata, at least in part retrospectively with reference to the meticulously drawn sections. This procedure means that not all later disturbances are necessarily identified and isolated and also that a large number of units must be assigned to two or more strata where the horizontal excavation cuts across the sloping floors or surfaces.[43] Thus, unfortunately, the potential for contamination between different levels was high.

Jung does, in principle, distinguish between material which was, without question, in use at the time of the destruction of each building and that which is earlier. Ideally such material would comprise vessels on destruction floors, either complete or mendable. It is therefore important to distinguish between 'complete' vessels and those which are represented only by a sherd or two. He does not, however, define such vessels, either in terms of the proportion preserved or in the catalogue entries themselves. In fact only around a dozen vessels in Schichten 14a and 12, when Mycenaean pottery is most common, are reported as over c 1/3 complete. Of these, three were reconstructed from widely scattered sherds, suggesting a lengthy period between breakage and final deposition.

In addition, despite the apparent precision of his analysis, little attempt is made in Jung's discussion to distinguish between the sherd material which is earlier and, accordingly, residual and that which might have been in recent use. Activities such as terracing, levelling and pit-digging can all bring earlier material into later contexts, whilst a particular feature of mud-brick construction, very obvious at Mycenae and Tiryns, for example, is the use of sherds as packing material in the mortar courses. These are then released when a wall collapses or decays with the result that in some contexts, 50% or more of the pottery is residual. These

circumstances are of course easier to observe with the pottery production of the Mycenaean heartland than in the local, simply-decorated products of the periphery.

As a matter of principle, therefore, only the latest material in any stratigraphic unit can be relied on to provide secure dating evidence and, in the case of locally-produced Mycenaean pottery, this is hard to determine. In these circumstances the pottery from any level can only be a *terminus post quem* rather than a *terminus ad quem*.

*The sequence of strata and the style of the pottery from each*.

Unlike Assiros, where the streets and other essentials of the settlement plan, once established, were retained through most of the settlement's history, the structures at Kastanas vary quite considerably from stratum (Schicht) to stratum and suggest a much lower level of social stability than at Assiros. This makes questions about continuity of occupation harder to answer. We are less convinced than Hänsel about interpreting the changes of architecture to be seen in Schichten 13 and 10 as the result of new population groups but these do not affect the dating evidence.[44] Destruction by fire was even more common than at Assiros whilst, at least in the part of the mound excavated, there is no sign of the granaries or concentrated storage which has been found at Assiros. We are also less persuaded than Jung that it is possible to apply the fine divisions of Mycenaean LH IIIC pottery used in southern Greece to the local products of Macedonia.

Schichten 16-15 are characterised by post-framed mud-brick structures: Schicht 16 has a variety of separate buildings and was brought to an end by a widespread destruction fire. Schicht 15, above it, had a range of structures with the same building methods but largely unrelated in plan to their predecessors. Imported LH IIIB pottery is present in small quantities in both strata[45] while local production/imitation is minimal. Jung regards several pieces as belonging to the final stages of this period[46] which places the destruction very close to the end of that period.

The site of one of these structures - the Ellipsenhaus - was reused in the following Schicht 14b for a single apsidal building, also of mud-brick, set in a wide courtyard area with rectangular structures further east. The fire which brought this phase to an end was obvious in every part of the site and left a number of artefacts in situ on the floors. This is the period in which locally produced Mycenaean pottery first predominates and exhibits characteristics which Jung dates, on the basis of parallels with southern Greek material, to LH IIIC Early. He notes, however, the presence of one half of a small jar (catalogue number 91) decorated with a fringed spiral motif not otherwise known until LH IIIC Late. He comments that it does not match the date of the rest of the material in this stratum and even in Schicht 13 would be 'very innovative'.[47] He prefers to explain this anomaly as a local imitation of the Macedonian matt-painted style unconnected with the appearance of such motifs in southern Greece in LH IIIC Late. Accepting or rejecting this explanation has, of course, considerable implications for the relative chronology of the whole LH IIIC sequence at Kastanas.

Rebuilding followed (Schicht 14a) with the same feature of a large, probably apsidal, building in an open courtyard and traces of buildings in other areas. This phase too was brought to an end by fire leaving a relatively deep deposit of mud-brick debris on the floors. Pottery of all kinds was rare and Jung suggests a latest date of LH IIIC entwickelt (Developed).[48] Given the generally comminuted condition of the sherds both construction and destruction of the buildings in this stratum could be even later.

Reconstruction in the following phase (Schicht 13) used a very different building technique with post-framing and a lighter wattle and daub type infill rather than the solid bricks of earlier phases. It is not clear from the report how soon the reconstruction took place but Hänsel notes a number of innovations in this stratum which suggest different agricultural and animal husbandry practices.[49] He attributes these to the arrival of a new population group – though one which, on the whole, used the same pottery as their predecessors, whilst Mycenaean style pottery had gone out of fashion for the time being. Several different construction episodes were detected but it is not clear what time span these represent.

Jung defines the limited amount of Mycenaean pottery found in Schicht 13 as LH IIIC 'fortgeschritten' (Advanced)[50] but there is no way of determining how much is residual. It is never easy to establish which are the floor levels of buildings with post-framed wattle and daub walls, as opposed to the levels into which the posts were set. This is especially problematic when floor levels are not identified and followed during excavation. These circumstances indicate that the date he suggests for the pottery is the *earliest* possible *terminus post quem*.

Schicht 12 and the following phase (11) see another change in plan and building technique. Mud-bricks are again used for the walls and the posts associated with them, beside rather than in the course of the walls, seem to be subsidiary rather than a framework. The different rooms now adjoin to form larger complexes rather than being single structures. The structures of these two phases and the associated features such as storage bins against the walls, are, for the first time, comparable with those already described for Assiros Phases 6 and 2, but the room dimensions at 6m x 4m resemble those of Assiros Iron Age Phases 3 and 2, rather than those of the Bronze Age. The settlements of both 12 and 11 were brought to an end by fire-destructions.

Schicht 12 is not subdivided by the excavators although a shallow sequence of occupation levels is shown in the published sections.[51] It contains a large number of Mycenaean sherds and a few larger fragments which are defined by Jung as LH IIIC fortgeschritten-spät (Advanced-Late) whilst also noting the presence of Sub-Mycenaean types. As with the earlier levels, it may be questioned how far this material is residual and how much dates to the final use of the structures before their destruction.[52] An additional problem is raised by the Proto-Geometric date of the latest pottery which is reported throughout the deposits associated with Schicht 12. The hallmark of Proto-Geometric – concentric circles drawn with a multiple brush on a compass – is well represented at Kastanas.

The vast majority of the pottery from Schicht 12 (as from all others) is local hand-made ware but signs of the innovations which are characteristic of the *Macedonian* Iron Age, for example channelled ware and twisted handles are still limited.[53] One piece of the distinctive Macedonian wheel-made grey category is reported from Schicht 13 at Kastanas[54] whilst at Toumba Thessalonikis, this ware is already regularly attested in the final LBA Stratum 3.[55] At Assiros a handful of grey ware sherds belong to Phase 4 and this ware steadily becomes more common in Phases 3-1. These wares cannot be dated independently of the Mycenaean and Proto-Geometric pottery with which they are associated, except in the case of Assiros Phase 4 through the $^{14}$C determinations for that phase. Thus the examples of this grey ware and of channelled ware from Kastanas Schicht 12 do not *necessarily* antedate the start of the Proto-Geometric period since that is the date of the latest identifiable material in the level. Any suggestion that they are earlier relies on the evidence from Toumba Thessalonikis just mentioned. Many of these early pieces of grey ware are imitations of the Mycenaean deep bowl shape and are surely a descendant of Mycenaean-style pottery which has been fired (atypically) in a kiln with a reducing atmosphere. When the ware does become common, the greater proportion of the sherds can be identified as from mugs with a single high-swung handle.

In the scenario favoured by Hänsel and Jung, the structures located in Schicht 12 were in use over a long period – around 150 years in conventional terms - from mid LH IIIC to Proto-Geometric. The date of the destruction of the buildings in Schicht 12 is clear, provided we reject, as highly improbable, the remote possibility of the co-existence *in use* of LH IIIC Advanced pottery and the first use of Proto-Geometric – wherever invented – a possibility which Jung himself does indeed reject.[56] The construction date is much harder to determine. Jung assumes that the earliest pottery – LH IIIC Advanced – provides this date and proposes a continuous use of the structures during which the new Mycenaean pottery styles were introduced and then supplanted by others. It was, apparently, not possible, despite the best efforts of the excavators, to detect subtle stratigraphic differences arising from the passage of time and there does not seem to be any differentiation in the depth at which pieces of the successive styles were found.

Three other scenarios can be explored which do not require such an improbably long time span for the use of the structures in this stratum. The first is that all of the Proto-Geometric pottery is intrusive, as the result of the cutting of post-pits associated with the new structures of Schicht 11 and that the phase does belong to the latest LH IIIC period. The second is that all the Mycenaean pottery in Schicht 12 was residual. Indeed, Jung notes that joins were made in several vessels between widely scattered fragments.[57] Clearly the new mud-brick structures of this period required substantial foundations and the multi-roomed plan first used at this point may well have entailed considerable terracing – which would have resulted in the incorporation of quantities of earlier material in later levels. This would date the level to the Early Proto-Geometric period before the most characteristic of the EIA features came into use.

The third possible explanation is that the destruction level of Schicht 12 reflects a period when Mycenaean-style pottery – especially deep bowls coated with a dark slip inside and out – was still being produced in some quantity, and alongside these, the first vessels with the recognisable characteristics of Proto-Geometric pottery. Jung acknowledges this in noting the presence, side by side on the destruction floor, of an Early Iron Age monochrome Skyphos (deep bowl) and a late Mycenaean Krater and the 'Konservatismus der makedonischen Topfereien der Früheisenzeit'.[58] In terms of its relationship to other regions of Greece, this would also date the final use of the buildings in Schicht 12 to the Early Proto-Geometric period. There is nothing to establish the date of construction beyond the latest date attributable to the underlying stratum, Schicht 13.

By the following level (Schicht 11) at Kastanas, Iron Age features in the hand-made wares have become more frequent. In the wheel-made category, no more than a small proportion of the whole, several decorative elements of Mycenaean pottery persist alongside the innovations of the Middle Proto-Geometric style.[59] Sherds of the wheel-made grey category are now frequent.

The architectural style changes again for the following phase (Schicht 10), with a return to lightly constructed timber-framed buildings, while the majority of the pottery is local. This is predominantly hand-made but also includes a small amount of the linear-decorated wheel-made pottery of Macedonian Late Proto-Geometric – Geometric date.[60] In neither case can the stages of development yet be dated on the basis of relative chronologies. Unusually, the end of this phase is not marked by traces of fire. In summary, following Jung's definition of the latest pottery in each stratum, Schichten 14b, 14a & 13 represent a continuous development through LH IIIC Early to Advanced and Schicht 12 a long period from LH IIIC Advanced to Early Proto-Geometric, though a hiatus between 13 and 12 cannot be ruled out (Table S7).

**Table S7: Kastanas: summary of dates on basis of Jung's rejection of the LH III C Late parallels for catalogue number 91**

| Schicht | Construction:- earlier than latest pottery, but not necessarily earlier than 'residual' material | Latest use & destruction on basis of latest pottery as defined by Jung – *rejecting* evidence of cat. no 91. | Latest use on basis of pottery date of following stratum |
|---|---|---|---|
| 11 | ≥MPG | MPG | >LPG/G |
| 12 | ≥EPG | EPG | >MPG |
| 13 | ≥LH IIIC Advanced | LH IIIC Advanced | >EPG |
| 14a | ≥LH IIIC Developed | LH IIIC Developed | >LH IIIC Advanced |
| 14b | =LH IIIC Early | LH IIIC Early | >LH IIIC Developed |
| 15 | ≥LH IIIB end | LH IIIB end | >LH IIIC Early |

If, however, we assign the jar with fringed spiral (91) in Schicht 14b to the same date as its LH IIIC Late parallels, the strata become much more compressed in time so that Schichten 14a and 13 both belong to the LH IIIC late stage, and there is either a large gap in the earlier stages unrepresented by any building level or a long duration for Schicht 14b (Table S8).

**Table S8: Kastanas: summary of dates if LH IIIC Late parallels for catalogue number 91 are accepted**

| Schicht | Construction:- earlier than latest pottery, but not necessarily earlier than 'residual' material | Latest use & destruction on basis of latest pottery as defined by Jung – *accepting* evidence of cat. no 91. | Latest use on basis of pottery date of following stratum |
|---|---|---|---|
| 11 | ≥MPG | MPG | >LPG/G |
| 12 | ≥EPG | EPG | >MPG |
| 13 | LH IIIC Late | LH IIIC Late | >EPG |
| 14a | LH IIIC Late | LH IIIC Late | LH IIIC Late |
| 14b | ≥LH IIIC Late | LH IIIC Late | LH IIIC Late |
| 15 | ≥LH IIIB end | LH IIIB end | >LH IIIC Early |

*The $^{14}$C samples and the absolute dates they provide for each stratum at Kastanas*

Since the evidence provided by the pottery is, to say the least, ambiguous, it is appropriate to consider whether the evidence of the absolute dates provides any clarification. 46 $^{14}$C samples of seeds and wood charcoal were collected during the course of the excavation, analysed in Berlin, Koln and Kiel and presented by Willcomm as a supplement to the excavation report on the stratigraphy and structures.[61] No building

timbers survived in the large fragments suitable for dendrochronological wiggle matching.[62] It was already clear at this point that the $^{14}C$ dates and the expected historical-archaeological dates were offset by around 100 years in places – "müßen die archäologischen Zeiten für Mitte und Ende des SH III B um rund 100 Jahre zum Älteren vershoben werden".[63] Since then considerable efforts have been made by Jung and Weninger to explain the clear discrepancy between conventional and absolute dating as the result of generalised problems with the wood charcoal samples and an atypical diet in the case of some of the animal bones which were submitted for analysis as a check on the earlier results.[64]

Manning and Weninger had already conducted a first reassessment by using a technique they called 'archaeological wiggle-matching'. In contrast to dendrochronological wiggle-matching in which the number of tree rings between each sample can be counted and the intervals between samples are precisely defined, archaeological wiggle-matching involves estimating the duration of each of the phases (with the help of the expected duration of the different pottery styles) in order to create a framework for the determination of the 'fit' of the result to the calibration curve.[65] As already noted, the continuity of occupation at Kastanas is not sufficiently clear to rule out intervals of abandonment and, in any case, estimating the duration of the period of time represented by a specific stratigraphic unit is a matter of guesswork rather than logical deduction, especially if predetermined on pseudo-historical grounds by the expected length of a pottery phase. In addition, charcoal samples from a building with a 50 year life span could date as early as its construction or as late as the most recent repairs to the roof, unless embedded in the original structure. It is not legitimate to base any conclusions on the assumption that all dated material (charcoals, seeds or animal bone) belong to the first use of a building without independent evidence. The pottery may represent the whole span of use but only the latest provides evidence of date, as already discussed.

Jung and Weninger called into question the validity of the individual wood charcoals as representing the age of the strata in which they were found, pointing out that in most cases it was not possible to determine what point in a tree's growth-ring sequence they reflect. As a result, an arbitrary point during the life-span of any tree was suggested as a 'norm', resulting in a standard offset between the rings preserved in the charcoal sample and the bark (ie the cutting date) of around 70 years. Such a presumption is, of course, no more than speculative.

A further 8 'group' samples of animal bones[66] were measured in Köln as a check on the old wood effect and, at first sight, the results match the previous determinations from the wood charcoals satisfactorily. In principle the value of these for determining the absolute dates should have been much stronger, since there is no issue about cutting dates in animals with a short life expectancy. These results, however, were again called into question by Jung and Weninger on the basis of the $\delta^{13}C$ values which indicated, for two samples including cattle bone, a predominantly C4 plant diet resulting from grazing, perhaps, on the salt marshes which bordered the estuary of the Axios river in that period.[67] This raised the possibility of an offset based on the marine carbon $^{14}C$ uptake but the $\delta^{15}N$ values which could have been definitive, were not determined.

The unusual dietary signal interestingly matches the evidence from Assiros where the diet of most cattle sampled from Phase 5 on must have included a substantial amount of millet or other C4 plant material (Main text Figure 5). This is very unlikely in the Assiros region to be the result of free grazing. Clearly there were distinctive animal husbandry practices at both sites but it is too early to assess the wider social or historical significance of this observation. It does suggest the strong possibility, however, of an alternative explanation for the $\delta^{13}C$ values for some of the bones at Kastanas that does not invoke a marine dietary influence and a possible age offset.

The Kastanas data is summarised in two diagrams published in 2004 and repeated in 2009 (Figures S9 & S10).[68]

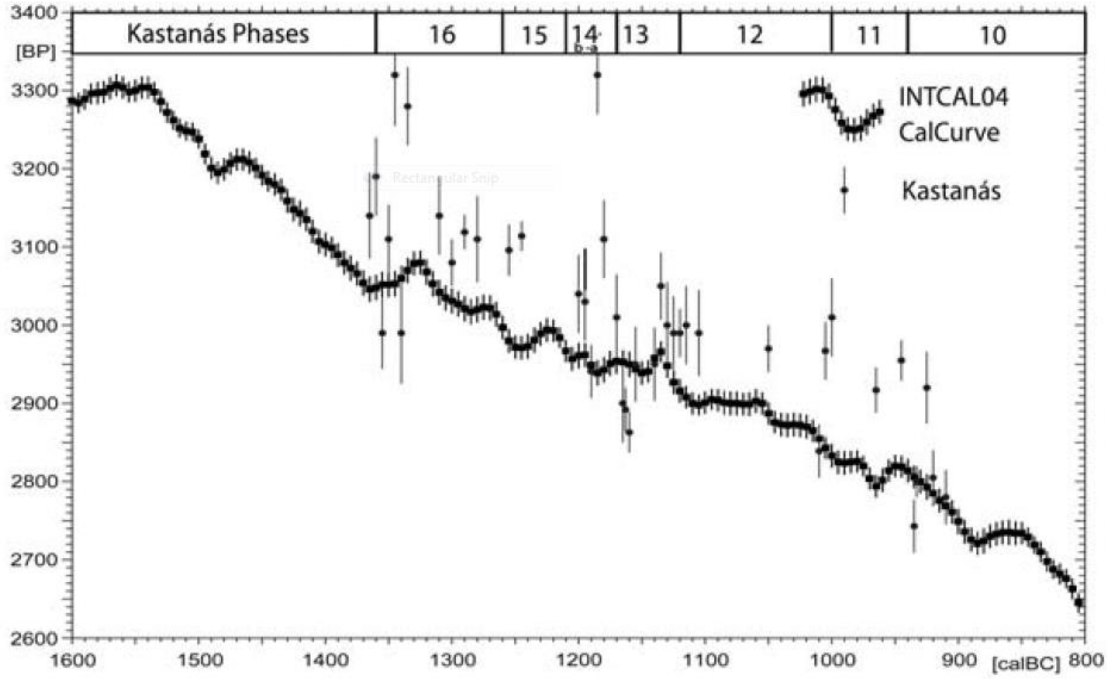


**Figure S9: In the Kastanas plot preferred by Weninger and Jung the determinations are given their expected historical ages – resulting in a systematic offset from the calibration curve of around 100 yrs.**

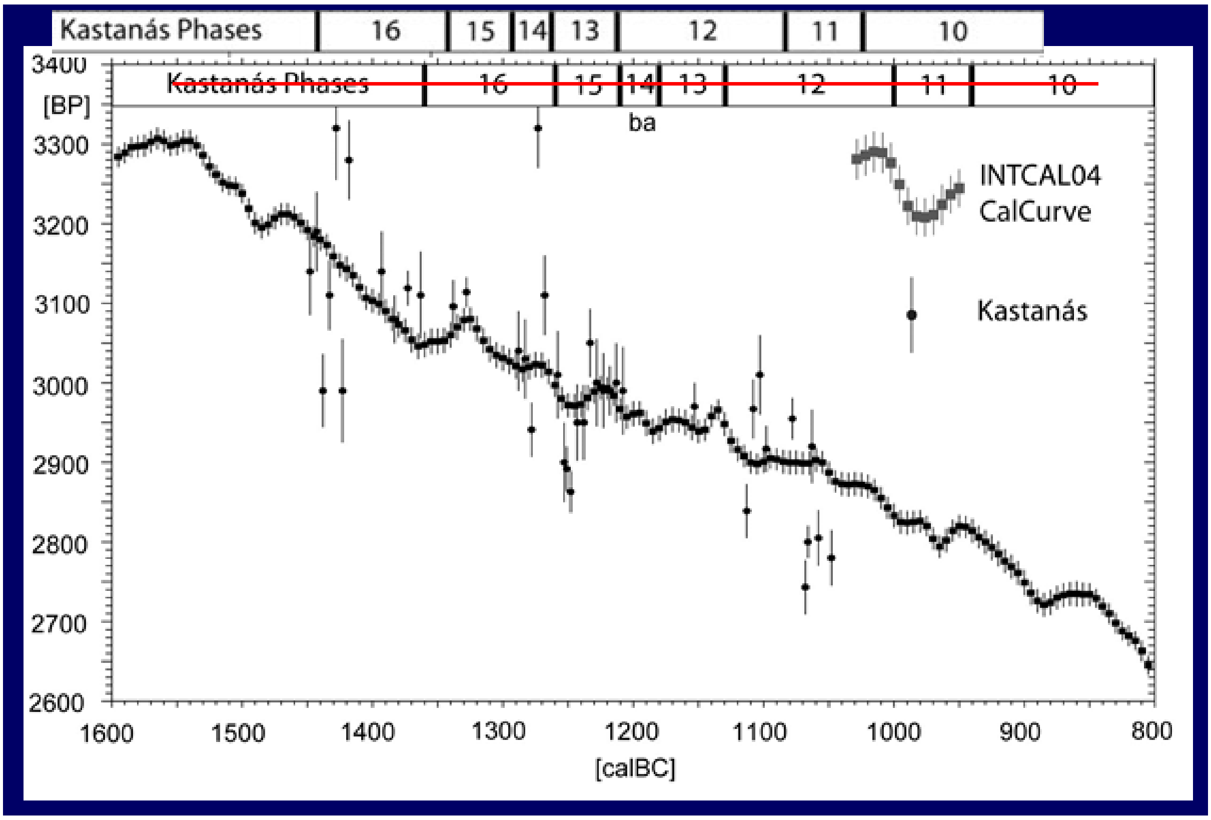


**Figure S10: In the Kastanas plot rejected by Weninger and Jung, the dates fit with reasonable conformity to the calibration curve.**

(NB we have corrected the stratum/Schicht headings to match the determinations correctly.)

In the first of these, the dates are plotted on the *x* axis according to expected historical dates and on the *y* axis in terms of uncalibrated BP determinations. The result, unsurprisingly, is that the plot shows the vast majority of the samples to be around 100 years *earlier* than the expected date. In the second diagram they are plotted in their natural positions[69] and they follow the calibration curve reasonably well. The diagrams also include determinations from the site of Toumba Thessalonikis[70] which are broadly compatible with the pattern which can be observed at Assiros and Kastanas.

Convinced that the Kastanas evidence could be made to fit the expected chronology, whereas that from Assiros could not, Weninger and Jung pointed to apparent weaknesses in the Assiros dating evidence, in order to reject it.[71] This rejection was based on the assumption that the Assiros timbers were old wood and that fragments of a single vessel could travel down through the stratigraphy as well as up. The new animal bone determinations presented above show this to be unfounded. It is unfortunate that those who wish to maintain the status quo of conventional chronology have accepted their arguments without sufficient critical appraisal.[72] We note, however, that despite their general rejection of the $^{14}C$ offset at Kastanas, Weninger and Jung do suggest raising the start date of LH IIIC to 1240/30 BC which is much closer to their own (rejected) evidence from Kastanas and that from Assiros.[73]

The Kastanas data, however, taken at face value, provide a series of determinations from a stratigraphic sequence. Their potential for analysis with Bayesian modelling is self-evident – without any prior assumptions derived from estimates of the length of time represented by any stratum or from conventional chronologies. We have therefore run a Bayesian model for the site and show the results in Figure S11.

**Figure S11: Bayesian model of Kastanas $^{14}$C data without prior assumptions about period length or assumed 'historical' date**. See Appendix 2, Table S12 for the model code.

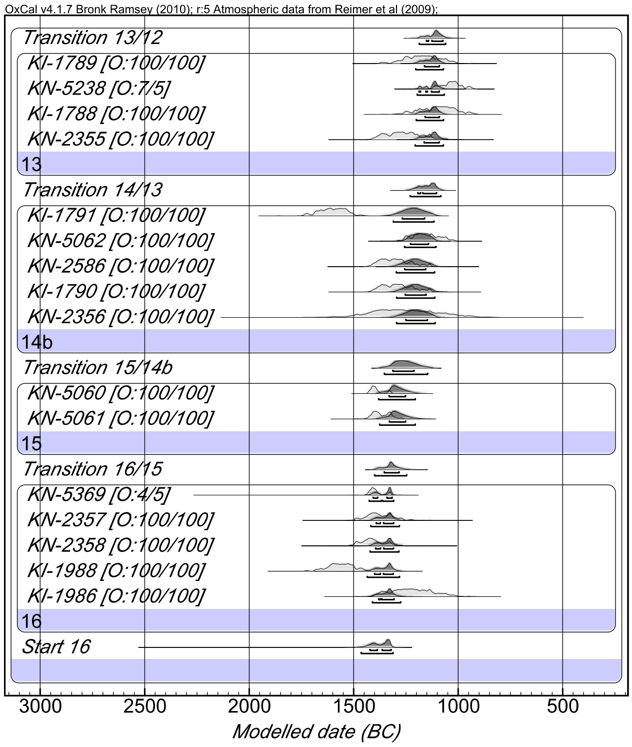


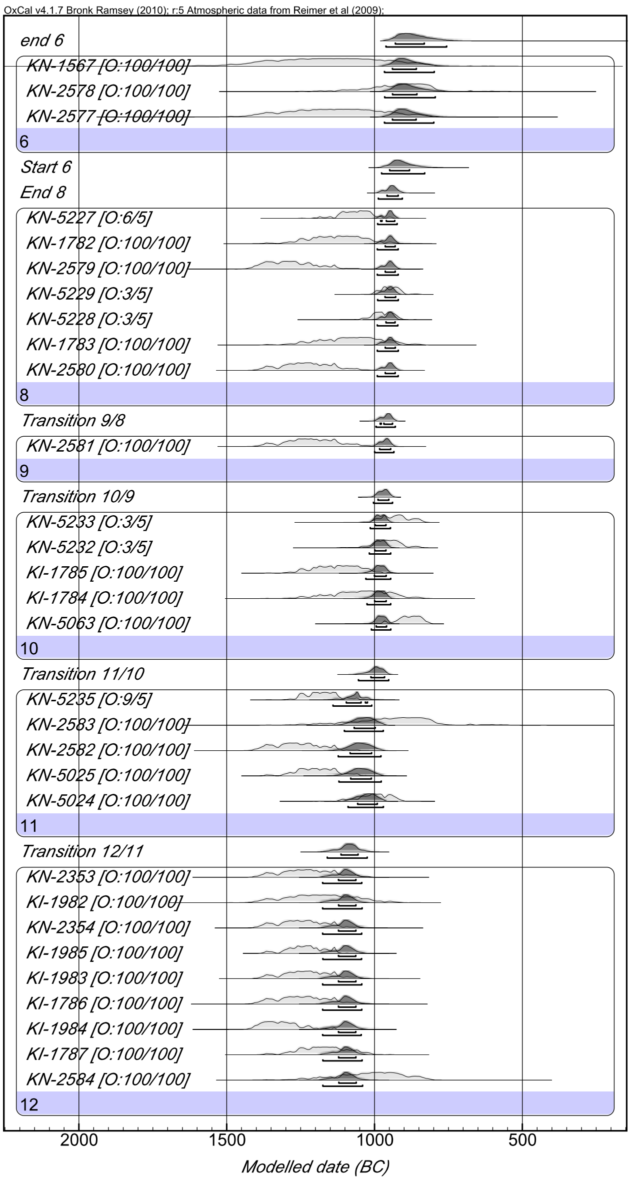


8

The results of this modelling can readily be compared with Jung's estimates of historical age on the basis of either of the interpretations of date discussed above and the comparisons for both are presented in Table S9.

**Table S9: Kastanas: The $^{14}$C derived date ranges for the samples from each stratum/Schicht plotted against the destruction dates for each level. The date ranges represent the start and end boundaries that constrain the phases of Kastanas and are presented at 2σ, 95.4% probability.**

| Latest use and destruction on basis of latest pottery as defined by Jung – *rejecting* evidence of catalogue no 91. | Conventional date | Schicht | Absolute date range (BC) of Kastanas phases at 2σ, 95.4% probability. | | Convent-ional date | Latest use and destruction on basis of latest pottery as defined by Jung – *accepting* evidence of catalogue no 91. |
|---|---|---|---|---|---|---|
| | | | Start | End | | |
| MPG | 1000-950 | 11 | 1148-1021 | 1054-952 | 1000-950 | MPG |
| EPG | 1050-1000 | 12 | 1179-1052 | 1148-1021 | 1050-1000 | EPG |
| LH IIIC Advanced | 1150-1000 | 13 | 1208-1077 | 1179-1052 | 1100-1050 | LH IIIC Late |
| LH IIIC Developed | 1190-1150 | 14a | | | 1100-1050 | LH IIIC Late |
| LH IIIC Early | 1230-1190 | 14b | 1321-1136 | 1208-1077 | 1230-1190 | LH IIIC Late |
| LH IIIB end | 1320-1230 | 15 | 1382-1232 | 1321-1136 | 1320-1230 | LH IIIB end |

Whilst the dates at the beginning and end of the sequence in both interpretations are offset by around 100 years from those conventionally used, that for those in the middle depends on the understanding of what is the latest material in each Schicht. Although Jung regards Schicht 12 as of long duration, perhaps around 150 years, this is not supported by the dates for the strata and the modelling which shows a duration of 0-68 years at 2σ, 95.4% probability. The rejection of a 150 year time span for Kastanas Schicht 12 on the grounds already discussed, is thus entirely justified by an absolute date for the preceding Schicht 13, only 30-40 years earlier than that for Schicht 12. In the same way, although he regards Schicht 14b as of short duration in early LH IIIC, the broad date range of samples within the Schicht (Fig S8) indicates a long duration (we calculate it at 38-163 yrs at 1σ, 68.2% prob. and 0-196 yrs at 2σ, 95.4%), which suggests the 'natural' dating of the pot with fringed spirals (catalogue number 91) to LH IIIC Late is correct.

*Absolute dates at Assiros and Kastanas: conformity.*

The Bayesian modelled age ranges for Kastanas conform without difficulty to those from Assiros at the beginning and end of the sequence, even allowing for such indeterminables as the relationship of charcoal samples to the buildings in the debris of which they were found. The absolute date range for Assiros Phase 8

which may, but does not certainly, represent an earlier stage of LH IIIB pottery than Kastanas Schicht 15, is 1360-1310 BC whilst that for Phase 3 with Early Proto-Geometric, similar to that in Kastanas Schicht 12, is 1094-1071 BC

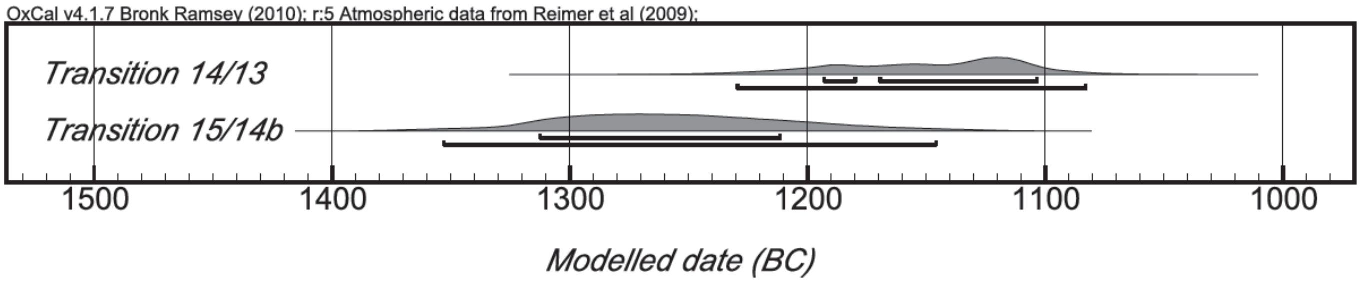


**Figure S12: Kastanas: The start and end dates for stratum/Schicht 14b as derived from the Bayesian model.**

The absolute date of the first introduction of locally made LH IIIC styles to each site seen in Assiros Phase 7 and Kastanas Schicht 14b is also significant. At Assiros the date of Phase 7 (start 1341-1282 BC and end 1312-1264 BC, at 2σ, 95.4% probability) is supported by the sequence of dates from the following Phases 6 & 5 which show a steady progression: Phase 5, start at 1265-1203 BC, end at 1232-1145 BC (at 2σ, 95.4% probability: see main text Table 1 for full sequence of start and end dates for each phase). As already noted for Kastanas, first, there is one small jar which should date to a much later stage of LH IIIC than accepted by Jung, and second the variation between the determined dates for the individual samples is very broad, as confirmed by the plot of the start and end dates for this phase (Figure S12).

As discussed in the main text, it is difficult to place Assiros Phase 4 (which has no independently datable pottery) in relation to the transition from the LBA to EIA in Southern Greece. At Toumba Thessalonikis the first local EIA features already appear in Level 3, well before the appearance of Proto-Geometric concentric circle decoration in Level 2A. Tentatively, we suggest that this phase at Assiros, the first to show the characteristic features of the local EIA pottery, is in fact contemporary with the final stages of Late Helladic IIIC. Comparison of the relative absolute dates for the start of this phase and of Kastanas Schicht 12 (Figure S13) confirms that this is likely. The relative chronology of key levels at each of the three sites is set out in Table S10.

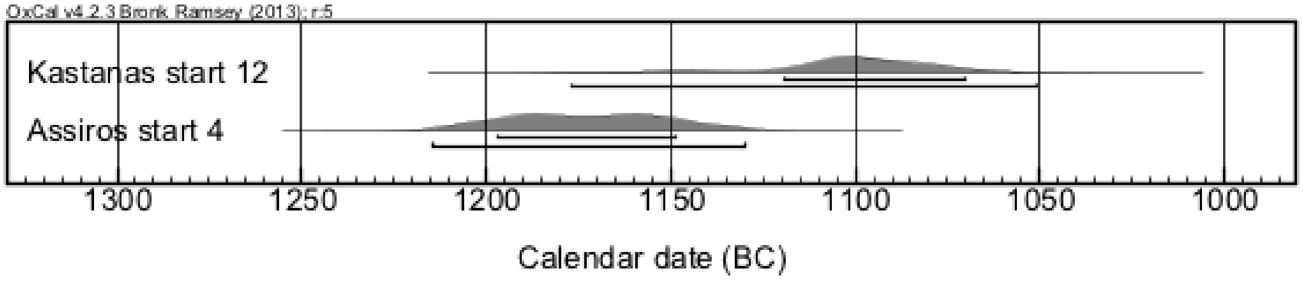


**Figure S13: The start dates of Assiros Phase 4 and Kastanas Schicht 12 compared.**

**Table S10: The relative chronology of the building levels at Assiros, Kastanas and Toumba Thessalonikis can be roughly established on the basis of the first appearance of Late Helladic IIIC, local Iron Age and Proto-Geometric pottery respectively.**

| pottery style | first appears at | | |
|---|---|---|---|
| | ASSIROS | KASTANAS | TOUMBA THESSALONIKIS |
| Early Proto-Geometric | Phase 3 | Schicht 12** | Level 2A |
| Local Iron Age | Phase 4 | Schicht 12** | Levels 3-2B |
| LHIII C | Phases 7-6-5 | Schichten 14B-14A-13 | Levels 4D-4C-4B-4A |

** Schicht 12 at Kastanas contains pottery of all stages from LH IIIC Advanced to Proto-Geometric and presents problems of interpretation as discussed above.

There can be little doubt that the absolute dates derived for Assiros are correct or that they can be related firmly to the Mycenaean pottery styles of each phase – provided only that no attempt is made to 'fine tune' the date of locally produced pottery entirely unsuited to such a process. Moreover, a careful reassessment of the Kastanas stratigraphy as set out above, combined with the date ranges for each stratum obtained from a Bayesian model without recourse to assumed historical chronologies, demonstrates clearly that the offset from the conventional chronology originally observed by Willcomm for Kastanas is a real one. There is nothing in the Kastanas dataset to contradict that derived from a careful selection of both long and short life samples at Assiros. It is also, despite the best efforts of Jung and others to prove otherwise, irreconcilable with the presently accepted quasi-historically based chronologies.

**Section E): the high-low debate in the Mediterranean region.**

Unsurprisingly, given the relative scarcity of high quality sequences of $^{14}C$ data from around the Mediterranean, the first Assiros results from the building timbers have been invoked in support of those who favour a 'high chronology'[74] for the Bronze Age/Iron Age transition in Italy, Spain and North Africa, rejected by those who favour a low one[75] or discussed from a neutral standpoint.[76] They are also relevant to the discussion of the stratified sequences from Israel and other parts of the Levant and their relationship to the historical chronology of the kingdom of Judah where they are welcomed or rejected in equal measure in the pages of recent editions of Radiocarbon.

Van der Plicht, Bruins and Nijboer use the Bayesian analysis of dates from Tel Dan and Tel Rehov, among others in Israel, from Latium Vetus in Central Italy, from Huelva in Spain and from Carthage to support a high chronology – also offset by around 50-70 years.

Fantalkin, Finkelstein and Piasetzky counter Bruins *et al.* with a re-examination of the $^{14}C$ data from Israel in favour of a low chronology but tellingly, in their introduction, they introduce Greek pottery as a valuable part of the discussion:

"*We argue that a reliable way to provide absolute dates for the Iron Age in the central and western Mediterranean is by employing a combination of well-identified Greek pottery found in well-stratified sites and radiometric results from short-lived samples. For the time being, this combination exists only in the Levant, and provides an anchor for Greek chronology, which supports the Conventional Chronology for the Aegean Basin, which corresponds to the Low Chronology in the Levant*."

Jung and Weninger take a similar view on the basis of their reading of the Kastanas dates and argue that their conventional chronology for Kastanas fits well with the north-Alpine dendrochronology dates.[77] They present the associations between pottery of Sub-Mycenaean type and Italian LBA metal types at Roccavecchia in Apulia, and between the metal types and Hallstatt B1 in south Germany as a reliable chain of evidence, though metal types are notoriously even more difficult than pottery types to date precisely.

The new results from Assiros suggest that this 'anchor for Greek chronology' is not where Fantalkin *et al.* perceive it to be and that it provides no support for their interpretation. In their reply van der Plicht *et al.* reject the low chronology and note:

"*Our main conclusions are that $^{14}C$ dating supports an Iron Age High Chronology for the above sites, though more $^{14}C$ dating is required, particularly in Greece and the central and western Mediterranean region, to substantiate and refine the current state of knowledge*."

Assiros has provided a significant contribution to precisely this issue.[78] It remains to be seen how far colleagues can be persuaded of the contribution of independent Bayesian analysis and the creation of chronologies which are truly independent of the *conventional* chronologies.[79] The precise strength of the

Bayesian method is in its ability to incorporate all data – rather than picking and choosing between individual elements of the story according to how well they match scholarly preconceptions.

**Figures and Tables**

Figure S1: Intervals calculated for each Assiros phase.

Figure S2: Bayesian model for Assiros; *left* Phases 9-6, *right* Phases 5-1.

Figure S3: Assiros Phase 9 with granaries.

Figure S4: Assiros Phase 6 plan.

Figure S5: Plan of village in Phase 2.

Figure S6: Dendro wiggle match diagram for Phase 9 timber.

Figure S7: *Left:* Distribution of Group 1 Proto-Geometric amphorae *after* Catling 1998, 156. *Right:* Distribution of Proto-Geometric pottery in the Levant *after* Lemos 2002, 229, Map 8.

Figure S8: Find spots diagram for pieces of the PG amphora (green) and the 4 timbers used for dendrochronology and the DWM (red).

Figure S9: In the Kastanas plot preferred by Weninger and Jung, the determinations are given their expected historical ages – resulting in a systematic offset from the calibration curve of around 100 years.

Figure S10: In the Kastanas plot rejected by Weninger and Jung, the dates fit with reasonable conformity to the calibration curve.

Figure S11: Bayesian model of Kastanas $^{14}C$ data, without prior assumptions about period length or assumed 'historical' date.

Figure S12: Kastanas: The start and end dates for stratum/Schicht 14b as derived from the Bayesian model.

Figure S13: The start dates of Assiros Phase 4 and Kastanas Schicht 12 compared.

Table S1: Radiocarbon AMS dates from Assiros obtained from bones with associated analytical and context data.

Table S2: Oak timbers used for dendrochronology.

Table S3: Oak timbers used for dendrochronological wiggle-matching (DWM).

Table S4: Crop seeds from Phase 9 Granary Room 9, and pithos in Phase 6 Room 20.

Table S5: Results of the Bayesian modelling.

Table S6: Results of the Bayesian outlier modelling.

Table S7: Kastanas: summary of date on basis of Jung's rejection of the LH IIIC Late parallels for catalogue number 91.

Table S8: Kastanas: summary of date if LH IIIC Late parallels for catalogue number 91 are accepted.

Table S9: Kastanas: The $^{14}C$ derived date ranges for the samples from each stratum/Schicht plotted against the destruction dates for each level.

Table S10: The relative chronology of key building levels at Assiros, Kastanas and Toumba Thessalonikis.

Table S11 (Appendix 1): Model code for the Bayesian age model of Assiros.

Table S12 (Appendix 2): Model code for the Bayesian age model of Kastanas.

## End notes

[1] Brock *et al*., 2007.
[2] Kromer and Münnich 1992.
[3] Funding for excavation and study was generously provided by the Alexander von Humboldt Stiftung, British Academy, British School at Athens, Cambridge Classics Faculty, Institute for Aegean Prehistory, Russell Trust and University of Birmingham as well a number of private sponsors.
[4] Wardle 1980, 1987, 1988, 1989, Wardle and Wardle 2007. For a full list of Assiros publications see www.artsweb.bham.ac.uk/aha/kaw/assiros/pubs.html
[5] Jones *et al*., 1986, 96-103. Wardle 1989, 460–2.
[6] Wardle 1993, 126–7.
[7] Wardle 1988, 385, pl.57e.
[8] Wardle 1993, 130-133; Buxeda I Garrigos J. *et al.* 2003
[9] See the discussion of LH IIIB/LH IIIC chronology in Warren and Hankey 1989, considerably augmented by the series of conference volumes published by the Mykenischen Kommission Wien – Deger-Jalkotzy and Zavadil 2003, Deger-Jalkotzy and Zavadil 2007; Deger Jalkotzy and Bächle 2009.
[10] In contrast to Jung's definitions for the pottery from Kastanas which seem to us to be over-optimistic.
[11] Wardle 1999, 443-460
[12] The relationship between the Iron Age pottery at Assiros, in Thrace and as far afield as Troy have been discussed in Wardle *et al*. 2007.
[13] '…. in Phases 4B and 4A, Perhaps the most important innovation was the introduction, although in very small numbers, of a new wheel made ware, the Fine Gray Ware…', Andreou 2009, 20.
[14] An extended discussion with details of the finds can be found in Wardle and Wardle, 2000, 653-673. In earlier reports our best guesstimate of a date for this phase was during or at the end of the 8$^{th}$ C BC on the basis of two small fragments of pottery identified by J.N. Coldstream as 'Geometric or Archaic.' The new $^{14}$C dating sequence indicates a much earlier date for the occupation in this level.
[15] Fagerström 1988, Mazarakis-Ainian, 1977.
[16] Jones 1981, 1987.
[17] Brown *et al*. 1999
[18] Heaton *et al*. 2009.
[19] Marianne Newton at Cornell was responsible for the selection of the samples from this and other suitable timbers sent to Heidelberg for $^{14}$C determination.
[20] Heaton *et al*. 2009.
[21] Newton *et al.* 2005.
[22] We regard it as very unlikely that all four of the timbers cross-matched by dendrochronology with the same growth period and cutting dates were ones that had been reused.-
[23] Desborough 1952, 294.
[24] Weninger and Jung 2009, 392-393.
[25] Monchambert 1996; Yon *et al*., 2000, 69.
[26] Desborough 1964, 240-241.
[27] Warren and Hankey 1989, 164.
[28] Lemos 2002, 26.
[29] Coldstream 2003, 253-254. Coldstream and Mazar 2003.
[30] Mazar 2005; Mazar and Bronk Ramsey, 2008; Finkelstein & Piatsezky 2010, etc.
[31] Sherratt 2005, 117.
[32] Jacob Felsch 1988.
[33] Maier *et al.* 2009, 71.
[34] Jung 2002.
[35] Andreou 2009.
[36] Andreou 2009, 24.
[37] Jung does not entertain this possibility in his detailed study of the material from Schicht 12, 2002, 226.
[38] Weninger and Jung, 2009, 388: 'To sum up the evidence from Ássiros, the redating of the start of the Greek Early Iron Age at this site is based on one single, partially preserved PG vessel scattered through two consecutive settlement phases, which are dated by four timbers that could have been reused from earlier buildings. This does not, however, imply that the dendro-dates from Ássiros are not useful. If the dated timbers were reused construction material from Phase 4 and the PG amphora is EPG in date, from these results it follows that the end of Sub-Mycenaean must be sought sometime during the 11th century BC – clearly much later than assumed by Kenneth Wardle.'
[39] Stratigraphy: Hänsel 1989, Mycenaean pottery: Jung 2002, local pottery: Hochstetter 1984. A convenient overview of the changes in settlement plan at Kastanas is provided in Hänsel 2002, 16-17 whilst Jung's assessment of the date of each Schicht is tabulated in Jung 2002, 228 abb. 80.
[40] Although Jung discusses the difficulties arising from the predominantly local character of the pottery (2002, 218) and the lack of comparable material, even from central Greece, he nevertheless uses the fine distinctions established primarily in the Argolid for the Mycenaean pottery from Kastanas.

[41] It is true as Weninger and Jung note (2009, 378) that Newton, Wardle and Kuniholm (2005, 185-187) did not take 'adequately into consideration the stratigraphical evidence underlying these [Kastanas] dates'. If we had, we would have shown at that time that it is *far weaker* than Jung believes.
[42] Horejs (2012, 144), without discussion remarks 'scheint diese postulierte Hochdatierung schlüssig zu widerlegen', a phrase which is taken up by Rutter (2013) 'She persuasively argues that attempts to raise the date for the beginning of the Protogeometric era (the beginning of the Iron Age) by up to 40-50 years on the basis of dendrochronological samples excavated at Assiros must be rejected.' There is no *argument* in the passage to which he refers, simply a reference to Weninger and Jung's discussion (2009).
[43] With the Mycenaean pottery alone, three plates of illustrated sherds (Taf. 20-22) are assigned to Schichten 12/13 and no less than eight to Schichten 11/12 (Taf. 42-49).
[44] Hänsel 2002, 15, 18.
[45] Jung 2002, 221, Abb. 74, reports only 8 fragments of small open vessels from Schicht 15.
[46] Catalogue numbers: 19 ('Er mußt sogar schon ans Ende dieser Periode gehören'), 20, 39, 46-47: Jung 2002, 221.
[47] '…widerspricht dies nicht nur der Datierung der übrigen Funde der Schicht 14b, sondern auch der der Funde aus der nächstjüngeren Schicht 14a. Selbst in der Schicht 13 würde das Gefäss einen sehr innovativen Eindruck machen': Jung 2002, 222.
[48] Jung 2002, 223.
[49] Hänsel in Jung 2002.
[50] Jung 2002, 224.
[51] Jung cites Hänsel 1989, 171, 188 for the depth of the deposits (Schichtenmachtigkeit) of this stratum. The sections show, however, that the majority of this depth is fallen mud-brick debris rather than *occupation* debris.
[52] At the most, only five registered vessels from Schicht 12 (249, 264, 272, 312 and 283) have more than 1/3 preservation and, of these, the last was found scattered over a wide area: Jung 2002.
[53] Hochstetter 1984. If it is accepted, as Jung does, that the PG pottery in Schicht 12 postdates much of the Mycenaean in the same level, there can be no argument for regarding the channelled wares and twisted handles recorded from this stratum as any earlier than the PG period. The assumption that both types belong to an earlier part of the LH III C period thus relies on two sherds of each recorded from Phase 13. Cf. Weninger and Jung 2009, 338.
[54] Jung 2002, pl. 65.530: Jung 2007.
[55] Andreou, Kotsakis and Chourmouziadis 1990, 390; Krachtopoulou and Touloumis 1993, 292.
[56] Jung 2002, 226.
[57] Jung 2002, 225: 'das nur ein Teil dieser besser erhaltenen Stucke in situ gefunden warden, bei einen ganzen Reihe liessen sich nämlich Anpassungen an weit entfernt gefundene Scherben finden.'
[58] Jung 2002, 227.
[59] Jung 2002, 227-228.
[60] Hänsel 2002, 17.
[61] Willkomm 1989.
[62] Weninger and Jung 2009, 375.
[63] Willkomm 1989, 410.
[64] Tentatively in Jung & Weninger 2004, 221-222; and more definitively in Weninger and Jung 2009, 409, fig. 4.
[65] Manning and Weninger, 1992.
[66] Because of the small size of available bone samples, several fragments from different species were used for all but one of these samples: Weninger and Jung 2009, 403, Tab. 3.
[67] Jung 'Viewing the entire set of bone dates (tab. 2), we cannot exclude a relation between higher $^{13}$C-levels and higher $^{14}$C-ages.'
[68] Weninger and Jung 2009, 408.
[69] The 'Schicht' headings in Weninger and Jung 2009, were not realigned for this diagram but remain in the position 'chosen' for them on the basis of the conventional chronology. We have superimposed a set of headings which represent the correct relationship between strata and samples.
[70] Set out in Andreou 2009, 29 Table 2 and discussed in Jung, Weninger and Andreou 2009, 191-193.
[71] Weninger and Jung, 2009, 388.
[72] Fantalkin *et al.* 2011, 186. See also note 51.
[73] Jung and Weninger 2002; 2004, 221
[74] Van der Plicht *et al.* 2009, Bruins *et al.* 2011. The discussion of these issues is complicated but not vitiated by the different dates at which each regional 'Iron Age' is customarily started, and indeed by the fact the term itself is a very loose one and certainly does not imply a sudden and wholesale replacement of bronze by iron for tools, weapons etc.
[75] Maier *et al.* 2009; Finkelstein and Piasetzky, 2010a; Fantalkin *et al.* 2011.
[76] Hagens 2006; Fantuzzi T. (undated) http://www.academia.edu/899838/The_debate_on_the_Absolute_Chronology_for_the_End_of_the_Late_Bronze_Age_and_the_Beginning_of_the_Early_Iron_Age_in_Greece_in_its_Mediterranean_Context.
[77] Weninger and Jung 2009, 389-392.
[78] As noted by Bruins *et al.*, 2011, 213.
[79] We note that Finkelstein and Piasetzky (2010b, 376, fig 3) have employed 'historical constraints' in creating a Bayesian model for the southern Levant which inevitably undermine its independence.

**Bibliography**

Andreou St, Kotsakis K, Chourmouziadis G (1990) Ανασκαφή στην Τούμπα της Θεσσαλονίκης 1989, *Egnatia* 2, 381–404.

Andreou S (2009) Stratified wheel made pottery deposits and absolute chronology of the LBA to the EIA transition at Thessaloniki Toumba, in. Deger-Jalkotzy and Bächle eds (2009) 15-40.

Brock F Higham, TFG, Ditchfield P, Bronk Ramsey C (2010) Current Pretreatment Methods for AMS Radiocarbon Dating at the Oxford Radiocarbon Accelerator Unit (ORAU), *Radiocarbon* 52(1), 103-112.

Bronk Ramsey C (2001) Development of the radiocarbon calibration program OxCal, *Radiocarbon* 43, 355-363.

Bronk Ramsey C (2009a) Bayesian analysis of radiocarbon dates, *Radiocarbon* 51(1), 337-360.

Bronk Ramsey C (2009b) Dealing with outliers and offsets in radiocarbon dating, *Radiocarbon* 51(3), 1023-1045.

Brown TA, Nelson DE, Vogel JS, Southon JR (1988) Improved collagen extraction by modified Longin method, *Radiocarbon* 30(2), 171-7.

Brown TA, Allaby RG, Sallares R, Jones G (1999) Ancient DNA in charred wheats: taxonomic identification of mixed and single grains, *Ancient Biomolecules* 2, 185-93.

Bruins HJ, Nijboer A and Van der Plicht J (2011) Iron Age Mediterranean Chronology: A Reply, *Radiocarbon* **53(1)**, 199-220.

Buxeda i Garrigos J, Jones RE, Kilikoglou V, Levi ST, Maniatis Y, Mitchell J, Vagnetti L, Wardle KA and Andreou S (2003) Technology transfer at the periphery of the Mycenaean World: the cases of Mycenaean pottery found in Central Macedonia (Greece) and the Plain of Sybaris (Italy), *Archaeometry* 45:2, 263-284.

Catling RWV (1998) The Typology of the Protogeometric and Subgeometric pottery from Troia and its Aegean context, *Studia Troica* 8, 153-164.

Coldstream JN (2003) Some Aegean Reactions to the Chronological debate in the Southern Levant, *Tel Aviv* 30:2.

Coldstream JN, Mazar A (2003) Greek Pottery from Tel Rehov and Iron Age Chronology, *Israel Exploration Journal* 53/1, 29-48.

Deger-Jalkotzy S and Bächle A eds. (2009) *LH IIIC Chronology and Synchronisms III: LH IIIC Late and the Transition to the Early Iron Age. Proceedings of the International Workshop at the Austrian Academy of Sciences at Vienna, February 23rd and 24th, 2007*, Veröffentlichungen der Mykenischen Kommission, Band: 30 Vienna.

Deger-Jalkotzy S and Zavadil M eds. (2003) *LH III C Chronology and Synchronisms: Proceedings of the international workshop held at the Austrian Academy of Sciences at Vienna, May 7th and 8th, 2001*, Veröffentlichungen der Mykenischen Kommission, Band: 20, Vienna

Deger-Jalkotzy S and Zavadil M (2007) *LH III C Chronology and Synchronisms II LH III C Middle: Proceedings of the international workshop held at the Austrian Academy of Sciences at Vienna, October 29th and 30th, 2004*. Veröffentlichungen der Mykenischen Kommission, Band: 28, Vienna.

Desborough VRd'A (1952) *Protogeometric Pottery,* Oxford.

Desborough VRd'A (1964) *The Last Mycenaeans and Their Successors,* Oxford.

Fagerström K (1988) *Greek Iron Age architecture: developments through changing times, SIMA* 81, Göteborg, 106-110.

Fantalkin A, Finkelstein I, Piasetzky E (2011) Iron Age Mediterranean Chronology: A Rejoinder, *Radiocarbon* 53(1), 179-198.

Fantuzzi (undated) http://www.academia.edu/899838/The_debate_on_the_Absolute_Chronology_for_the_End_of_the_Late_Bronze_Age_and_the_Beginning_of_the_Early_Iron_Age_in_Greece_in_its_Mediterranean_Context.

Finkelstein I, Piasetzky E (2010a) The Iron Age I/IIA transition in the Levant: a reply to Mazar and Bronk Ramsey and a new perspective, *Radiocarbon* 52(4), 1667–1680.

Finkelstein I, Piasetzky E (2010b) Radiocarbon dating the Iron Age in the Levant: a Bayesian model for six ceramic phases and six transitions, *Antiquity* 84(324), 374–385.

Hagens G (2006) Testing the limits; radiocarbon dating and the end of the Late Bronze Age *Radiocarbon* 48(1), 83–100.

Hänsel B (1989) *Kastanas. Ausgrabungen in einem Siedlungshügel der Bronze- und Eisenzeit Makedoniens 1975–1979. Die Grabung und der Baubefund*, Prähist. Arch. Südosteuropa 7, Berlin.

Hänsel B (2002) Zusammenfassende Bemerkungen zum Siedlungsverlauf in Kastanas – Kontinuität und Bevölkerungswandel, in Jung 2002, 11–27.

Heaton TH, Jones G, Halstead P, Tsipropoulos T (2009) Variations in the 13C/12C ratios of modern wheat grain, and implications for interpreting data from Bronze Age Assiros Toumba, Greece, *Journal of Archaeological Science* 36, 2224-33.

Higham T, Bronk Ramsey Ch, Owen C (2004) *Radiocarbon and Archaeology. Proceedings of the 4$^{th}$ Symposium, Oxford 2002,* Oxford.

Hochstetter A (1984) *Kastanas. Ausgrabungen in einem Siedlunghügel der Bronze- und Eisenzeit Makedoniens 1975-1979. Die handgemachte Keramik. Schichten 19 bis 1*, Prähist. Arch. Südosteuropa 3. Berlin.

Horejs B (2012) 6. Zur Chronologie der makedonischen Spätbronzezeit in Pavuk P and Horejs B, *Mittel- und spätbronzezeitliche Keramik Griechenlands. Sammlung Fritz Schachermeyr*, Faszikel III. Denkschriften der philosophisch-historischen Klasse, 439; Veröffentlichungen der Mykenischen Kommission, 31. Wien: Verlag der Österreichischen Akademie der Wissenschaften, 144-145.

Jacob-Felsch M (1988) Compass–drawn Concentric Circles in Vase Painting: A Problem of Relative Chronology at the End of the Bronze Age, in Eds. French, E.B., Wardle, K.A., *Problems in Greek Prehistory*, Bristol, 193-199.

Jones G (1981) Crop processing at Assiros Toumba - a taphonomic study, *Zeitschrift für Archäologie* 15, 105-11.

Jones G (1987) Agricultural practice in Greek Prehistory, *Annual of the British School at Athens* 82, 115-23.

Jones G, Wardle K, Halstead P and Wardle D (1986) Crop Storage at Assiros Toumba, *Scientific American*, March 1986, 96-103.

Jung R (2002) *Kastanas. Ausgrabungen in einem Siedlungshügel der Bronze- und Eisenzeit Makedoniens 1975–1979. Die Drehscheibenkeramik der Schichten 19 bis 11*, Prähist. Arch. Südosteuropa 18. Kiel.

Jung R, Weninger B (2002), Appendix: Zur Realität der Diskrepanzen zwischen den kalibrierten $^{14}$C-Daten und der historisch-archäologischen Datierung in Kastanas, in Jung (2002) 281–298.

Jung R  Weninger B (2004) Kastanás and the Chronology of the Aegean Late Bronze and Early Iron Age, in Higham T, Bronk Ramsey C, Owen C, eds. (2004) 209–228.

Jung R, Andreou S, Weninger B (2009) Thessaloniki Toumba at the end of the Bronze and Beginning of the Iron Age, in Deger-Jalkotzy and Bächle eds. (2009) 183-202.

Jung R (2007) On the origin of the wheel-made Grey Ware found in Central Macedonia, http://www.aegeobalkanprehistory.net/article.php?id_art=4

Krachtopoulou A and Touloumis K (1993) Ανασκαφή Τούμπας 1990. Οι εργασίες στην κορυφή της Τούμπας, *Archaiologikon Ergon Makedonias kai Thrakis* 4, 1990, 289–297.

Kromer B, Münnich K.-O (1992) CO2 gas proportional counting in radiocarbon dating - review and perspective, in  Taylor RE, Long A, Kra RS eds., *Radiocarbon after Four Decades,* New York, Springer, 184-197.

Lemos IS (2002) *The Protogeometric Aegean. The Archaeology of the Late Eleventh and Tenth Centuries BC*, Oxford.

Levy TE and Higham T eds. (2005) *The Bible and Radiocarbon Dating: Archaeology, Text and Science*, London.

Maier AM, Fantalkin A, Zukerman A (2009) The earliest Greek import in the Iron Age Levant: new evidence from Tell Es-Safi/Gath, Israel, *Ancient West and East* 8, 57-80.

Manning S, Weninger B (1992) A Light in the Dark: Archaeological Wiggle Matching and the Absolute Chronology of the Close of the Aegean Late Bronze Age, *Antiquity* 66, 1992, 636–663.

Mazar A (2005) The Debate over the Chronology of the Iron Age in the Southern Levant: its history, the current situation and a suggested resolution, in Levy and Higham eds. (2005) 302-309.

Mazar A, Bronk Ramsey C (2008) 14C dates and the Iron Age chronology of Israel: a response, *Radiocarbon* 50(2), 159–80.

Mazarakis-Ainian A (1977) From rulers' dwellings to temples, *SIMA* 121, Göteborg, 43-113.

Monchambert J-Y (1996) Du Mycénien IIIC à Ougarit, *Orient-Express* 1996/2, 45.

Newton MW, Wardle KA, Kuniholm PI (2005a) A Dendrochronological $^{14}C$ Wiggle-Match for the Early Iron Age of north Greece: A contribution to the debate about this period in the Southern Levant, in Levy and Higham eds. (2005) 104-113.

Newton MW, Wardle KA, Kuniholm PI (2005a) Dendrochronology and Radiocarbon determinations from Assiros and the beginning of the Greek Iron Age, *Archaiologikon Ergon Makedonias kai Thrakis* 17(2003), 173-190. (Available at

http://dendro.cornell.edu/articles/newton2005.pdf)

Reimer PJ, Baillie MGL, Bard E, Bayliss A, Beck JW, Blackwell PG, Bronk Ramsey C, Buck CE, Burr G, Edwards RL, Friedrich M, Grootes PM, Guilderson TP, Hajdas I, Heaton TJ, Hogg AG, Hughen KA, Kaiser KF, Kromer B, McCormac FG, Manning, SW, Reimer RW, Richards DA, Southon JR, Talamo S, Turney CSM, van der Plicht J and Weyhenmeyer CE (2009) Intcal09 and Marine09 radiocarbon age calibration curves, 0–50,000 years cal BP, *Radiocarbon*, 51(4), 1111-1150.

Rutter JR (2013) review of Horejs (2012) in *Bryn Mawr Classical Review* 2013.05.36

Sherratt S (2005) High precision Dating and Archaeological Chronologies: revisiting an old problem, in Levy and Higham eds. (2005), 114-128.

Toffolo MB, Fantalkin A, Lemos IS, Felsch RCS, Niemeier W-D, Sanders GDR, Finkelstein I, Boaretto E (2013) Towards an Absolute Chronology for the Aegean Iron Age: New Radiocarbon Dates from Lefkandi, Kalapodi and Corinth, www.plosone.org/article/authors/info%3Adoi%2F10.1371%2Fjournal.pone.0083117;

Van der Plicht J, Bruins HJ, Nijboer A (2009) The Iron Age Around the Mediterranean: A High Chronology Perspective from the Groningen Radiocarbon Database, *Radiocarbon* 51(1), 213–242.

Wardle KA (1980) Excavations at Assiros Toumba 1975-1980, *Annual of the British School at Athens* 76, 229-260.

Wardle KA (1987) Excavation at Assiros Toumba 1986: A preliminary report, *Annual of the British School at Athens* 82, 313-329.

Wardle KA (1988) Excavation at Assiros Toumba 1987: A preliminary report, *Annual of the British School at Athens* 83, 375-387.

Wardle K.A (1989) Excavation at Assiros Toumba 1988: A preliminary report, *Annual of the British School at Athens* 84, 447-463.

Wardle KA (1993) Mycenaean Trade and Contact in Northern Greece Zerner C *et al.*, eds. (1993) *Wace and Blegen: Pottery as Evidence for Trade and Contact*, 117-141, Gieben.

Wardle KA (1999) Change or Continuity: Assiros Toumba at the transition from Bronze to Iron Age, *Archaiologikon Ergon Makedonias kai Thrakis* 10 1997, 443-460.

Wardle KA, Newton MW, Kuniholm PI (2007) Troy VIIB2 Revisited: The Date of the Transition from Bronze to Iron Age in the Northern Aegean, in eds. Todorova, H., Stefanovich, M., Ivanov, G., *The Struma/Strymon River Valley in Prehistory. Proceedings of the International Symposium Strymon Praehistoricus, Kjustendil–Blagoevgrad (Bulgaria), Serres–Amphipolis (Greece) 27.09–01.10.2004. (In The Steps of James Harvey Gaul Volume 2)* Sofia, 481-497.

Wardle KA and Wardle D (2007) Assiros Toumba: A brief history of the settlement, in Todorova, H., Stefanovich, M., Ivanov, G., eds. (2007) *The Struma/Strymon River Valley in Prehistory. Proceedings of the International Symposium Strymon Praehistoricus, Kjustendil–Blagoevgrad (Bulgaria), Serres–Amphipolis (Greece) 27.09–01.10.2004. (In The Steps of James Harvey Gaul Volume 2)* Sofia, 451-479.

Wardle KA and Wardle D (2000) Assiros: The Geometric Settlement in Adam Veleni P, ed. *Μνήμη Ιουλίας Βοκοτοπούλου*, Thessaloniki, 653-673.

Warren PM, Hankey V (1989) *Aegean Bronze Age Chronology*, Bristol.

Weninger B and Jung R (2009) Absolute chronology of the end of the Aegean Bronze Age in Deger-Jalkotzy and Bächle eds. (2009) 373–416.

Willkomm H (1989) Radiokohlenstoffdatierungen des Siedlungshügels Kastanas, in Hänsel 1989, 395–411.

Yon M, Karageorgis V, Hirschfeld N (2000) *Céramique Mycénienne d'Ougarit*. *Ras Shamra Ougarit* 13, Paris

**Appendix 1: Table S11: Model code for the Bayesian age model of Assiros.**

```
Plot()
{
 Outlier_Model("General",T(5),U(0,4),"t");
 Outlier_Model("SSimple",N(0,2),0,"s");
 Outlier_Model("RScaled",T(5),U(0,4),"r");
 Sequence()
 {
  Boundary("Start 9",U(-1800,-1200));
  D_Sequence()
  {
   R_Date("HD-24539",3109,22)
   {
    Outlier("RScaled", 0.05);
   };
   Gap(40);
   R_Date("HD-24538",3063,15)
   {
    Outlier("RScaled", 0.05);
   };
   Gap (6.5);
   Date( "Cutting Estimate dwm9")
   {
   };
  };
  Phase("Use 9")
  {
   R_Date("OxA-25069", 3096, 30)
   {
    Outlier("General", 0.05);
   };
   Interval("9");
  };
  Boundary("End use 9");
  Phase("Destruction by fire")
  {
   R_Date("HD-25508", 3027, 14)
   {
    Outlier("General", 0.05);
   };
```

```
 R_Date("HD-25509", 3071, 12)
 {
  Outlier("General", 0.05);
 };
 R_Date("HD-25512", 3022, 12)
 {
  Outlier("General", 0.05);
 };
 R_Date("HD-25514", 3050, 13)
 {
  Outlier("General", 0.05);
 };
 R_Date("HD-25522", 3057, 14)
 {
  Outlier("General", 0.05);
 };
 R_Date("HD-25523", 3043, 14)
 {
  Outlier("General", 0.05);
 };
 R_Date("HD-25524", 3023, 13)
 {
  Outlier("General", 0.05);
 };
 Interval("destruction event");
};
Boundary("Transition 9/8",U(-1700,-1200));
Phase("Recons 8")
{
};
Boundary();
Phase("Use 8")
{
 R_Date("OxA-25064", 3071, 30)
 {
  Outlier("General", 0.05);
 };
 R_Date("OxA-25065", 3098, 30)
 {
  Outlier("General", 0.05);
```

```
 };
 R_Date("OxA-25066", 3053, 30)
 {
  Outlier("General", 0.05);
 };
 R_Date("OxA-25067", 3094, 30)
 {
  Outlier("General", 0.05);
 };
 R_Date("OxA-25068", 3048, 29)
 {
  Outlier("General", 0.05);
 };
 Interval("8");
};
Boundary();
Phase("Destruction 8")
{
};
Boundary("Transition 8/7",U(-1600,-1150));
Phase("Construction 7");
Boundary();
Phase("Use 7")
{
 R_Date("OxA-25060", 3032, 29)
 {
  Outlier("General", 0.05);
 };
 R_Date("OxA-25061", 3048, 30)
 {
  Outlier("General", 0.05);
 };
 R_Date("OxA-25062", 3087, 31)
 {
  Outlier("General", 0.05);
 };
 R_Date("OxA-25063", 3070, 31)
 {
  Outlier("General", 0.05);
 };
```

```
 R_Date("BM-1429", 2940, 80)
 {
  Outlier("General", 0.05);
 };
 Interval("7");
};
Boundary("End use 7 to EQ",U(-1500,-1150));
Phase("Reconstruction 6")
{
 D_Sequence("dwm6")
 {
  R_Date("HD-23274",3045,17)
  {
   Outlier("RScaled", 0.05);
  };
  Gap(20.0);
  R_Date("HD-23273",3071,15)
  {
   Outlier("RScaled", 0.05);
  };
  Gap(24.5);
  R_Date("HD-21378",3085,37)
  {
   Outlier("RScaled", 0.05);
  };
  Gap(1.5);
  R_Date("HD-23275",3074,19)
  {
   Outlier("RScaled", 0.05);
  };
  Gap(10);
  R_Date("HD-23276",3030,22)
  {
   Outlier("RScaled", 0.05);
  };
  Gap (45);
  Date( "Cutting Estimate dwm6")
  {
  };
 };
```

```
};
Boundary("start use 6",U(-1400,-1100));
Phase("Use 6")
{
 R_Date("BM-1428", 2900, 70)
 {
  Outlier("General", 0.05);
 };
 R_Date("OxA-25056", 3074, 30)
 {
  Outlier("General", 0.05);
 };
 R_Date("OxA-25057", 3015, 30)
 {
  Outlier("General", 0.05);
 };
 R_Date("OxA-25058", 2976, 31)
 {
  Outlier("General", 0.05);
 };
 R_Date("OxA-25059", 3052, 30)
 {
  Outlier("General", 0.05);
 };
 Interval("6");
};
Boundary();
Phase("Destruction Fire")
{
 R_Date("HD-25517", 2922, 23)
 {
  Outlier("General", 0.05);
 };
};
Boundary("Transition 6/5",U(-1400,-1050));
Phase("Construction 5")
{
};
Boundary();
Phase("Use 5")
```

```
{
 R_Date("BM 1437", 2920, 75)
 {
  Outlier("General", 0.05);
 };
 R_Date("OxA-25051", 2974, 29)
 {
  Outlier("General", 0.05);
 };
 R_Date("OxA-25052", 2971, 30)
 {
  Outlier("General", 0.05);
 };
 R_Date("OxA-25053", 3027, 30)
 {
  Outlier("General", 0.05);
 };
 R_Date("OxA-25054", 2995, 30)
 {
  Outlier("General", 0.05);
 };
 R_Date("OxA-25055", 2969, 30)
 {
  Outlier("General", 0.05);
 };
 Interval("5");
};
Boundary();
Phase("Destruction Fire 5")
{
};
Boundary("Transition 5/4",U(-1350,-1050));
Phase("Construction 4")
{
};
Boundary();
Phase("Use 4")
{
 R_Date("OxA-24958", 3094, 33)
 {
```

```
  Outlier("General", 0.05);
 };
 R_Date("OxA-24955", 3010, 32)
 {
  Outlier("General", 0.05);
 };
 R_Date("OxA-24956", 2976, 31)
 {
  Outlier("General", 0.05);
 };
 R_Date("OxA-24954", 2933, 32)
 {
  Outlier("General", 0.05);
 };
 R_Date("OxA-24957", 2920, 31)
 {
  Outlier("General", 0.05);
 };
 R_Date("OxA-24959", 2911, 31)
 {
  Outlier("General", 0.05);
 };
 Interval("4");
};
Boundary();
Phase("Destruction 4 Earthquake?")
{
};
Boundary("Transition 4/3",U(-1300,-950));
Phase("Rebuilding 3")
{
 C_Date("Dendro C14 WM", -1080, 5.5);
};
Boundary();
Phase("Use 3")
{
 R_Date("OxA-24952", 3018, 30)
 {
  Outlier("General", 0.05);
 };
```

```
 R_Date("OxA-24953", 2995, 32)
 {
  Outlier("General", 0.05);
 };
 R_Date("OxA-24951", 2989, 31)
 {
  Outlier("General", 0.05);
 };
 R_Date("OxA-24950", 2916, 31)
 {
  Outlier("General", 0.05);
 };
 R_Combine("Room 00303 sus from floor")
 {
  Outlier("General", 0.05);
  R_Date("OxA-24949", 2982, 30)
  {
   Outlier("SSimple", 0.05);
  };
  R_Date("OxA-24948", 2924, 31)
  {
   Outlier("SSimple", 0.05);
  };
 };
 Interval("3");
 Date("Protogeometric amphora");
};
Boundary();
Phase("Destruction 3 Fire")
{
};
Boundary("Transition 3/2",U(-1200,-950));
Phase("Recons 2")
{
 D_Sequence("dwm3")
 {
  R_Date("HD-23249",2935,14)
  {
   Outlier("RScaled", 0.05);
  };
```

```
  Gap(20.5);
  R_Date("HD-23250",2962,16)
  {
   Outlier("RScaled", 0.05);
  };
  Gap(7);
  R_Date("HD-21076",2925,29)
  {
   Outlier("RScaled", 0.05);
  };
  Gap(10);
  R_Date("HD-23438",3009,22)
  {
   Outlier("RScaled", 0.05);
  };
  Gap(13);
  R_Date("HD-23254",2927,16)
  {
   Outlier("RScaled", 0.05);
  };
  Gap(20);
  R_Date("HD-23251",2929,16)
  {
   Outlier("RScaled", 0.05);
  };
  Gap(20);
  R_Date("HD-21077",2906,23)
  {
   Outlier("RScaled", 0.05);
  };
  Gap (12);
  Date( "Cutting Estimate dwm3")
  {
  };
 };
};
Boundary();
Phase("Use 2")
{
 R_Date("BM-1426", 2800, 75)
```

```
 {
  Outlier("General", 0.05);
 };
 R_Date("OxA-24947", 2928, 30)
 {
  Outlier("General", 0.05);
 };
 R_Date("OxA-24946", 2924, 29)
 {
  Outlier("General", 0.05);
 };
 R_Date("OxA-25073", 2969, 32)
 {
  Outlier("General", 0.05);
 };
 Interval("2");
};
Boundary();
Phase("Destruction Fire 2")
{
};
Boundary("Hiatus 2/1",U(-1200,-900));
Phase("Use 1")
{
 R_Date("OxA-25070", 2920, 29)
 {
  Outlier("General", 0.05);
 };
 R_Date("OxA-25071", 2900, 31)
 {
  Outlier("General", 0.05);
 };
 R_Date("OxA-25072", 2855, 30)
 {
  Outlier("General", 0.05);
 };
 Interval("1");
};
Boundary("End 1",U(-1150,-850));
};
```

```
};
```

**Appendix 2: Table S12: Model code for the Bayesian age model of Kastanas.**

Note that charcoal determinations are analysed using the Charcoal outlier model which places an outlier of 1.00 on each result.

```
Plot()
 {
  Outlier_Model("Charcoal",Exp(1,-10,0),U(0,3),"t");
  Outlier_Model("General",T(5),U(0,4),"t");
  Sequence()
  {
   Boundary("Start 16");
   Phase("16")
   {
    R_Date("KI-1986", 2990, 65)
    {
     Outlier("Charcoal", 1.00);
    };
    R_Date("KI-1988", 3280, 50)
    {
     Outlier("Charcoal", 1.00);
    };
    R_Date("KN-2358", 3140, 50)
    {
     Outlier("Charcoal", 1.00);
    };
    R_Date("KN-2357", 3110, 55)
    {
     Outlier("Charcoal", 1.00);
    };
    R_Date("KN-5369", 3119, 22)
    {
     Outlier("General", 0.05);
    };
```

```
};
Boundary("Transition 16/15");
Phase("15")
{
 R_Date("KN-5061", 3096, 33)
 {
  Outlier("Charcoal", 1.00);
 };
 R_Date("KN-5060", 3114, 19)
 {
  Outlier("Charcoal", 1.00);
 };
};
Boundary("Transition 15/14b");
Phase("14b")
{
 R_Date("KN-2356", 3030, 120)
 {
  Outlier("Charcoal", 1.00);
 };
 R_Date("KI-1790", 3030, 50)
 {
  Outlier("Charcoal", 1.00);
 };
 R_Date("KN-2586", 3040, 50)
 {
  Outlier("Charcoal", 1.00);
 };
 R_Date("KN-5062", 2941, 34)
 {
  Outlier("Charcoal", 1.00);
 };
 R_Date("KI-1791", 3320, 50)
```

```
 {
  Outlier("Charcoal", 1.00);
 };
};
Boundary("Transition 14/13");
Phase("13")
{
 R_Date("KN-2355", 3010, 55)
 {
  Outlier("Charcoal", 1.00);
 };
 R_Date("KI-1788", 2900, 50)
 {
  Outlier("Charcoal", 1.00);
 };
 R_Date("KN-5238", 2863, 26)
 {
  Outlier("General", 0.05);
 };
 R_Date("KI-1789", 2950, 48)
 {
  Outlier("Charcoal", 1.00);
 };
};
Boundary("Transition 13/12");
Phase("12")
{
 R_Date("KN-2584", 2830, 80)
 {
  Outlier("Charcoal", 1.00);
 };
 R_Date("KI-1787", 2950, 47)
 {
  Outlier("Charcoal", 1.00);
 };
 R_Date("KI-1984", 3050, 43)
```

```
 {
  Outlier("Charcoal", 1.00);
 };
 R_Date("KI-1786", 3000, 55)
 {
  Outlier("Charcoal", 1.00);
 };
 R_Date("KI-1983", 2990, 47)
 {
  Outlier("Charcoal", 1.00);
 };
 R_Date("KI-1985", 2990, 31)
 {
  Outlier("Charcoal", 1.00);
 };
 R_Date("KN-2354", 3000, 50)
 {
  Outlier("Charcoal", 1.00);
 };
 R_Date("KI-1982", 2960, 75)
 {
  Outlier("Charcoal", 1.00);
 };
 R_Date("KN-2353", 2990, 55)
 {
  Outlier("Charcoal", 1.00);
 };
};
Boundary("Transition 12/11");
Phase("11")
{
 R_Date("KN-5024", 2839, 34)
 {
  Outlier("Charcoal", 1.00);
 };
 R_Date("KN-5025", 2967, 37)
 {
  Outlier("Charcoal", 1.00);
 };
 R_Date("KN-2582", 3010, 50)
```

```
 {
  Outlier("Charcoal", 1.00);
 };
 R_Date("KN-2583", 2750, 110)
 {
  Outlier("Charcoal", 1.00);
 };
 R_Date("KN-5235", 2955, 26)
 {
  Outlier("General", 0.05);
 };
};
Boundary("Transition 11/10");
Phase("10")
{
 R_Date("KN-5063", 2743, 34)
 {
  Outlier("Charcoal", 1.00);
 };
 R_Date("KI-1784", 2860, 65)
 {
  Outlier("Charcoal", 1.00);
 };
 R_Date("KI-1785", 2920, 46)
 {
  Outlier("Charcoal", 1.00);
 };
 R_Date("KN-5232", 2805, 35)
 {
  Outlier("General", 0.05);
 };
 R_Date("KN-5233", 2780, 35)
 {
  Outlier("General", 0.05);
 };
};
Boundary("Transition 10/9");
Phase("9")
{
 R_Date("KN-2581", 2980, 50)
```

```
 {
  Outlier("Charcoal", 1.00);
 };
};
Boundary("Transition 9/8");
Phase("8")
{
 R_Date("KN-2580", 2990, 50)
 {
  Outlier("Charcoal", 1.00);
 };
 R_Date("KN-1783", 2880, 70)
 {
  Outlier("Charcoal", 1.00);
 };
 R_Date("KN-5228", 2822, 24)
 {
  Outlier("General", 0.05);
 };
 R_Date("KN-5229", 2795, 20)
 {
  Outlier("General", 0.05);
 };
 R_Date("KN-2579", 3030, 55)
 {
  Outlier("Charcoal", 1.00);
 };
 R_Date("KN-1782", 2920, 55)
 {
  Outlier("Charcoal", 1.00);
 };
 R_Date("KN-5227", 2881, 29)
 {
  Outlier("General", 0.05);
 };
};
Boundary("End 8");
Boundary("Start 6");
Phase("6")
{
```

```
  R_Date("KN-2577", 2920, 120)
  {
   Outlier("Charcoal", 1.00);
  };
  R_Date("KN-2578", 2730, 95)
  {
   Outlier("Charcoal", 1.00);
  };
  R_Date("KN-1567", 2930, 160)
  {
   Outlier("Charcoal", 1.00);
  };
 };
 Boundary("end 6");
};
};
```
